# Supplementary material for: Impact of violence on HIV outcomes among female sex workers: A global systematic review and meta-analysis
Source: PLoS Med. 2026 Oct 21;23(10):e1004977. doi: 10.1371/journal.pmed.1004977 (PMC13712678; doi:10.1371/journal.pmed.1004977)
Supplement: Supplementary file 1 [file pmed.1004977.s001.docx]

# Supplementary material

Supplement to: Dawe J, Mazhar KA, J, Khan SA, et al. Impact of violence on HIV outcomes among female sex workers: A global systematic review and meta-analysis

# Table of Contents

Table of Contents	2

Table of Tables	3

Table of Figures	4

Appendix A. Study Protocol	7

Appendix B. PRISMA checklist	12

Appendix C. Search Strategy	14

Appendix D. Studies excluded because of duplicate data	16

Appendix F. Variables included in data extraction	18

Appendix G. Study quality domains	19

Appendix H. Definitions of forms of violence	20

Appendix I. Definitions of HIV outcomes	20

Appendix J. Variables extracted for subgroup analyses	21

Appendix K. Characteristics of included studies (n=94)	22

Appendix L. Estimates of the association between sexual violence and HIV outcomes	32

Appendix M. Estimates of the association between physical violence and HIV outcomes	37

Appendix N. Estimates of the association between mixed violence and HIV outcomes	41

Appendix O. Estimates of the association between other forms of violence and HIV outcomes	47

Appendix P. Study quality scores of included studies	49

Appendix Q. Forest plots of all forms of violence estimates and prevalent HIV infection	53

Appendix R. Forest plots of physical violence estimates and prevalent HIV infection	58

Appendix S. Forest plots of sexual violence estimates and prevalent HIV infection	60

Appendix T. Forest plots of mixed violence estimates and prevalent HIV infection	63

Appendix U. Forest plots of other forms of violence estimates and prevalent HIV infection	65

Appendix V. Forest plots of violence and HIV testing	66

Appendix W. Forest plots of violence and ART outcomes	68

Appendix X. Forest plots of violence and HIV viral suppression	71

Appendix Y. Subgroup analyses	73

Appendix Z. Publication asymmetry	75

Appendix AA. Sensitivity analyses of RHO	83

Reference list	84

# Table of Tables

Table 1. PRISMA checklist	9

Table 2. Studies excluded because of duplicate data	13

Table 3. Studies identified through reference list searching	14

Table 4. Study quality domains	16

Table 5. Definitions of forms of violence	17

Table 6. Definitions of HIV outcomes	17

Table 7. Variables extracted for subgroup analyses	18

Table 8. Geographic and design characteristics of included studies	19

Table 9. Sex work and HIV characteristics of included studies	25

Table 10. Estimates of the association between sexual violence and HIV outcomes	29

Table 11. Estimates of the association between physical violence and HIV outcomes	34

Table 12. Estimates of the association between mixed violence and HIV outcomes	38

Table 13. Estimates of the association between other forms of violence and HIV outcomes	44

Table 14. Study quality scores of included studies	46

Table 15. Trim and fill analyses of experiences of violence and prevalent HIV infection	78

Table 16. Sensitivity analyses of all forms of violence and HIV outcomes for RHO=0.4, RHO=0.6 and RHO=0.8	79

# Table of Figures

Figure 1. Pooled unadjusted estimates of recent exposure to all forms of violence and prevalent HIV infection among female sex workers	53

Figure 2. Pooled unadjusted estimates of lifetime exposure to all forms of violence and prevalent HIV infection among female sex workers	54

Figure 3. Pooled adjusted estimates of recent exposure to all forms of violence and prevalent HIV infection among female sex workers	55

Figure 4. Pooled adjusted estimates of lifetime exposure to all forms of violence and prevalent HIV infection among female sex workers	55

Figure 5. Pooled combined unadjusted and adjusted estimates of recent exposure to all forms of violence and prevalent HIV infection among female sex workers	56

Figure 6. Pooled combined unadjusted and adjusted estimates of lifetime exposure to all forms of violence and prevalent HIV infection among female sex workers	57

Figure 7. Pooled unadjusted estimates of recent exposure to physical violence and prevalent HIV infection among female sex workers	58

Figure 8. Pooled unadjusted estimates of lifetime exposure to physical violence and prevalent HIV infection among female sex workers	58

Figure 9. Pooled adjusted estimates of recent exposure to physical violence and prevalent HIV infection among female sex workers	58

Figure 10. Pooled adjusted estimates of lifetime exposure to physical violence and prevalent HIV infection among female sex workers	59

Figure 11. Pooled combined unadjusted and adjusted estimates of recent exposure to physical violence and prevalent HIV infection among female sex workers	59

Figure 12. Pooled combined unadjusted and adjusted estimates of lifetime exposure to physical violence and prevalent HIV infection among female sex workers	59

Figure 13. Pooled unadjusted estimates of recent exposure to sexual violence and prevalent HIV infection among female sex workers	60

Figure 14. Pooled unadjusted estimates of lifetime exposure to sexual violence and prevalent HIV infection among female sex workers	60

Figure 15. Pooled adjusted estimates of recent exposure to sexual violence and prevalent HIV infection among female sex workers	61

Figure 16. Pooled adjusted estimates of lifetime exposure to sexual violence and prevalent HIV infection among female sex workers	61

Figure 17. Pooled combined unadjusted and adjusted estimates of recent exposure to sexual violence and prevalent HIV infection among female sex workers	62

Figure 18. Pooled combined unadjusted and adjusted estimates of lifetime exposure to sexual violence and prevalent HIV infection among female sex workers	62

Figure 19. Pooled unadjusted estimates of recent exposure to mixed violence and prevalent HIV infection among female sex workers	63

Figure 20. Pooled unadjusted estimates of lifetime exposure to mixed violence and prevalent HIV infection among female sex workers	63

Figure 21. Pooled adjusted estimates of recent exposure to mixed violence and prevalent HIV infection among female sex workers	63

Figure 22. Pooled adjusted estimates of lifetime exposure to mixed violence and prevalent HIV infection among female sex workers	64

Figure 23. Pooled combined unadjusted and adjusted estimates of recent exposure to mixed violence and prevalent HIV infection among female sex workers	64

Figure 24. Pooled combined unadjusted and adjusted estimates of lifetime exposure to mixed violence and prevalent HIV infection among female sex workers	64

Figure 25. Pooled unadjusted estimates of recent exposure to other forms of violence and prevalent HIV infection among female sex workers	65

Figure 26. Pooled adjusted estimates of recent exposure to other forms of violence and prevalent HIV infection among female sex workers	65

Figure 27. Pooled combined unadjusted and adjusted estimates of recent exposure to other forms of violence and prevalent HIV infection among female sex workers	65

Figure 28. Pooled unadjusted estimates of recent exposure to all forms of violence and HIV testing among female sex workers	66

Figure 29. Pooled unadjusted estimates of lifetime exposure to all forms of violence and HIV testing among female sex workers	66

Figure 30. Pooled adjusted estimates of recent exposure to all forms of violence and HIV testing among female sex workers	66

Figure 31. Pooled adjusted estimates of lifetime exposure to all forms of violence and HIV testing among female sex workers	67

Figure 32. Pooled combined unadjusted and adjusted estimates of recent exposure to all forms of violence and HIV testing among female sex workers	67

Figure 33. Pooled combined unadjusted and adjusted estimates of lifetime exposure to all forms of violence and HIV testing among female sex workers	67

Figure 34. Pooled unadjusted estimates of recent exposure to all forms of violence and ART use among female sex workers	68

Figure 35. Pooled unadjusted estimates of lifetime exposure to all forms of violence and ART use among female sex workers	68

Figure 36. Pooled adjusted estimates of recent exposure to all forms of violence and ART use among female sex workers	68

Figure 37. Pooled adjusted estimates of lifetime exposure to all forms of violence and ART use among female sex workers	69

Figure 38. Pooled combined unadjusted and adjusted estimates of recent exposure to all forms of violence and ART use among female sex workers	69

Figure 39. Pooled combined unadjusted and adjusted estimates of lifetime exposure to all forms of violence and ART use among female sex workers	69

Figure 40. Pooled unadjusted estimates of recent exposure to all forms of violence and ART adherence among female sex workers	70

Figure 41. Pooled adjusted estimates of recent exposure to all forms of violence and ART adherence among female sex workers	70

Figure 42. Pooled combined unadjusted and adjusted estimates of recent exposure to all forms of violence and ART adherence among female sex workers	70

Figure 43. Pooled unadjusted estimates of recent exposure to all forms of violence and HIV viral suppression among female sex workers	71

Figure 44. Pooled unadjusted estimates of lifetime exposure to all forms of violence and HIV viral suppression among female sex workers	71

Figure 45. Pooled adjusted estimates of lifetime exposure to all forms of violence and HIV viral suppression among female sex workers	71

Figure 46. Pooled combined unadjusted and adjusted estimates of recent exposure to all forms of violence and HIV viral suppression among female sex workers	71

Figure 47. Pooled combined unadjusted and adjusted estimates of lifetime exposure to all forms of violence and HIV viral suppression among female sex workers	72

Figure 48. Subgroup analyses of recent exposure to all forms of violence and HIV infection among female sex workers	73

Figure 49. Subgroup analyses of lifetime exposure to all forms of violence and HIV infection among female sex workers	74

Figure 50. Funnel plots for recent and lifetime exposure to all forms of violence and prevalent HIV infection	75

Figure 51. Funnel plots for recent and lifetime experiences of physical violence and prevalent HIV infection	76

Figure 52. Funnel plots for recent and lifetime experiences of sexual violence and prevalent HIV infection	76

Figure 53. Funnel plots for recent and lifetime experiences of physical and/or sexual violence and prevalent HIV infection	77

Figure 54. Funnel plots for recent and lifetime experiences of all forms of violence and HIV testing	78

Figure 55. Funnel plots for recent and lifetime experiences of all forms of violence and ART use	79

Figure 56. Funnel plots for recent experiences of all forms of violence and ART adherence	79

Figure 57. Funnel plots for recent and lifetime experiences of all forms of violence and HIV viral suppression	80

Figure 58. Recent and lifetime exposure to all forms of violence and prevalent HIV infection (Trim and Fill plots)	80

Figure 59. Recent and lifetime exposure to physical violence and prevalent HIV infection (Trim and Fill plots)	81

Figure 60. Recent and lifetime exposure to sexual violence and prevalent HIV infection (Trim and Fill plots)	81

Figure 61. Recent and lifetime exposure to mixed violence and prevalent HIV infection (Trim and Fill plots)	82

Figure 62. Pooled combined unadjusted and adjusted estimates of recent exposure to mixed violence and prevalent HIV infection among female sex workers excluding study with less than 100% FSWs	84

# Appendix A. Study Protocol

## The impact of societal enablers and barriers on HIV outcomes among the key populations of people who inject drugs, men who have sex with men, transgender women and female sex workers: a protocol for a systematic review and meta-analysis

### Citation

Joshua Dawe, Khadimul Mazhar, Saher Aijaz Khan, Adelina Artenie, Jack Stone, Matthew Hickman, Peter Vickerman. The impact of societal enablers and barriers on HIV outcomes among the key populations of people who inject drugs, men who have sex with men, transgender women and female sex workers: a protocol for a systematic review and meta-analysis. PROSPERO 2024 CRD42024531539 Available from: https://www.crd.york.ac.uk/prospero/display_record.php?ID=CRD42024531539

### Review question

Our overall aim is to summarise the impact of societal enablers and barriers on HIV outcomes among four key populations: people who inject drugs (PWID), men who have sex with men (MSM), transgender women (TGW) and female sex workers (FSW). Our review aims to focus on four HIV outcomes: (1) HIV prevalence, (2) HIV incidence, (3) ART coverage and (4) viral suppression. Three societal enablers and barriers will be reviewed: (1) stigma, (2) violence, and (3) legal and policy environments. For each key population, we have assessed the quality of existing global systematic reviews or large-scale multi-country studies to identify evidence gaps for each societal enabler.

Specifically, we have identified the following gaps in the evidence base for each key population:

1. People who inject drugs (PWID): Stigma and violence
2. Men who have sex with men (MSM): Stigma and violence
3. Sex workers (SW): Legal and Policy environment and stigma
4. Transgender people (TG): Legal and Policy environment, stigma and violence

Our specific aims (SA) are to:

- SA1: synthesise evidence from research which estimates the association between societal enablers and barriers and HIV outcomes among each key population;
- SA2: Explore sources of heterogeneity in pooled associations from SA1.

### Searches

Searches will be conducted in MEDLINE, Embase and PsycINFO.

The search string will be built using derivations and synonyms to describe each key population, societal enablers and barriers, and epidemiological studies. We will not apply language restrictions to the initial search. The search will be limited to studies published in 2010 and after.

### Types of study to be included

Studies that could be used to estimate the association between societal enablers and barriers and HIV outcomes among each key population empirically will be included, including observational studies (case-control/cohort/cross-sectional) and intervention trials.

### Inclusion criteria:

- Studies which report on the selected key populations (PWID/FSW/MSM/TG).
- Studies which report one of the societal enablers (stigma, violence, legal and policy environment);
- Studies which report data on HIV outcomes of interest.

### Exclusion criteria:

- Case studies, systematic reviews, qualitative studies, animal studies, commentaries, editorials, and modelling studies.
- Studies with samples sizes under n=40.
- Studies where a key population is not the primary population of study (i.e. PWID/FSW/MSM/TG) will be excluded. Specifically, we will exclude studies where less than 70% of the sample includes a key population, or those which do not present data for a key population specifically.
- Studies which do not include a HIV outcome of interest

### Condition or domain being studied

Studies that could be used to estimate the association between societal enablers and barriers and HIV outcomes among key populations at-risk of HIV.

### Participants/population

We will focus on four key populations:

1. People who inject drugs.
2. Men who have sex with men.
3. Transgender people.
4. Female sex workers.

### Intervention(s), exposure(s)

We will be exploring the impact of the following societal enablers and barriers on HIV outcomes:

- Stigma and discrimination
- Violence
- Legal and policy environment

### Comparator(s)/control

Comparisons: these will be defined according to the strata listed in the exposure section.

### Main outcome(s)

We will be studying four main HIV outcomes (HIV prevalence, HIV incidence, ART coverage and viral suppression).

### Measures of effect

We are interested in measure of effects (i.e., odds ratio, prevalence ratio, risk ratio, hazard ratio) which estimate the association between societal enablers and barriers and the main HIV outcomes, for each population. See aims SA1–SA2.

### Additional outcome(s)

None

### Data extraction (selection and coding)

Records identified through database searches (MEDLINE, Embase and PsycINFO) will be assessed for duplicates. Title and abstract screening will be conducted in Rayyan AI software.

During title and abstract screening, three authors (JD, KM and SK) will double-screen titles and abstracts and all disagreements will be resolved by mutual agreement after discussion. For any unresolved disagreements, a third author (AA) will be included.

All three authors will double screen all the studies included for full-text screening. We will identify studies that include any of the four exposures of interest. The screening and selection processes will be documented, in line with PRISMA guidelines.

Study data will be double-extracted to ensure consistency. The data to be extracted will include:

1. Study characteristics (e.g., lead author, journal name, year of publication, start/end dates, design, city/country,
1. sampling strategy, recruitment site, inclusion/exclusion criteria, attrition (where appropriate));
2. Descriptive characteristics of study sample (e.g., age, proportion young, proportion female, proportion with exposure
3. of interest) and the proportion of study samples experiencing societal enablers and barriers;
4. Characteristics of the exposure (e.g., societal enablers and barriers definition, time frame of measurement) and measures of association between structural risk factors and the outcomes included in SA1–SA2.

### Risk of bias (quality) assessment

Risk of bias assessment will be carried out independently by researchers and will be double screened/assessed to ensure consistency throughout, with disagreements resolved by discussion. We will use an adapted version of the Newcastle-Ottawa Scale (NOS) to assess risk of bias.

### Strategy for data synthesis

A minimum of three estimates will be required to generate a pooled effect between societal enablers and barriers and HIV outcomes. We will provide descriptive summaries of effect estimates (magnitude and range of effects) where a pooled effect cannot be calculated. Cross-sectional and case-control studies will be pooled together, where appropriate we will pool cross-sectional and longitudinal effects together.

### Descriptive data of each included study will be displayed in a summary table.

For each societal enabler and barrier, we will use random-effects meta-analyses to pool effect estimates comparing differences in outcomes between individuals exposed to a structural factor and individuals not exposed to a structural risk factor. Heterogeneity will be tested using the χ² test and quantified using the I²-statistic.

Where possible, we will perform sub-group and meta-regression analyses to investigate potential sources of heterogeneity. Publication bias will be explored using funnel plots and Egger's test.

### Analysis of subgroups or subsets

We plan to report findings according to global geographic and economic regions.

### Contact details for further information

Joshua Dawe

joshua.dawe@bristol.ac.uk

### Organisational affiliation of the review

University of Bristol

https://www.bristol.ac.uk/medical-school/

### Review team members and their organisational affiliations

Mr Joshua Dawe. Population Health Sciences, Bristol Medical School, University of Bristol

Mr Khadimul Mazhar. Population Health Sciences, Bristol Medical School, University of Bristol

Ms Saher Aijaz Khan. Population Health Sciences, Bristol Medical School, University of Bristol

Dr Adelina Artenie. Population Health Sciences, Bristol Medical School, University of Bristol

Dr Jack Stone. Population Health Sciences, Bristol Medical School, University of Bristol

Professor Matthew Hickman. Population Health Sciences, Bristol Medical School, University of Bristol

Professor Peter Vickerman. Population Health Sciences, Bristol Medical School, University of Bristol

### Type and method of review

Epidemiologic, Meta-analysis, Systematic review

### Anticipated or actual start date

26 March 2024

### Anticipated completion date

31 May 2024

### Funding sources/sponsors

The Joint United Nations Programme on HIV/AIDS (UNAIDS)

### Conflicts of interest

Language

English

### Country

England

### Stage of review

Review Ongoing

### Subject index terms status

Subject indexing assigned by CRD

### Subject index terms

Drug Users; Evidence Gaps; Female; HIV Infections; Homosexuality, Male; Humans; Incidence; Male; Meta-Analysis as Topic; Policy; Prevalence; Review Literature as Topic; Sex Workers; Sexual and Gender Minorities; Substance Abuse, Intravenous; Systematic Reviews as Topic; Transgender Persons; Violence

### Date of registration in PROSPERO

04 April 2024

### Date of first submission

02 April 2024

### Stage of review at time of this submission

| Stage | Started | Completed |
| --- | --- | --- |
| Preliminary searches | Yes | Yes |
| Piloting of the study selection process | Yes | Yes |
| Formal screening of search results against eligibility criteria | Yes | No |
| Data extraction | No | No |
| Risk of bias (quality) assessment | No | No |
| Data analysis | No | No |

# Appendix B. PRISMA checklist

Table 1. PRISMA checklist

| **Section and Topic** | **Item #** | **Checklist item** | **Location where item is reported** |
| --- | --- | --- | --- |
| **TITLE** |  |  |  |
| Title | 1 | Identify the report as a systematic review. | Title |
| **ABSTRACT** |  |  |  |
| Abstract | 2 | See the PRISMA 2020 for Abstracts checklist. | Abstract |
| **INTRODUCTION** |  |  |  |
| Rationale | 3 | Describe the rationale for the review in the context of existing knowledge. | Intro |
| Objectives | 4 | Provide an explicit statement of the objective(s) or question(s) the review addresses. | Intro |
| **METHODS** |  |  |  |
| Eligibility criteria | 5 | Specify the inclusion and exclusion criteria for the review and how studies were grouped for the syntheses. | Methods |
| Information sources | 6 | Specify all databases, registers, websites, organisations, reference lists and other sources searched or consulted to identify studies. Specify the date when each source was last searched or consulted. | Methods |
| Search strategy | 7 | Present the full search strategies for all databases, registers and websites, including any filters and limits used. | Appendices |
| Selection process | 8 | Specify the methods used to decide whether a study met the inclusion criteria of the review, including how many reviewers screened each record and each report retrieved, whether they worked independently, and if applicable, details of automation tools used in the process. | Methods |
| Data collection process | 9 | Specify the methods used to collect data from reports, including how many reviewers collected data from each report, whether they worked independently, any processes for obtaining or confirming data from study investigators, and if applicable, details of automation tools used in the process. | Methods |
| Data items | 10a | List and define all outcomes for which data were sought. Specify whether all results that were compatible with each outcome domain in each study were sought (e.g. for all measures, time points, analyses), and if not, the methods used to decide which results to collect. | Methods |
|  | 10b | List and define all other variables for which data were sought (e.g. participant and intervention characteristics, funding sources). Describe any assumptions made about any missing or unclear information. | Methods |
| Study risk of bias assessment | 11 | Specify the methods used to assess risk of bias in the included studies, including details of the tool(s) used, how many reviewers assessed each study and whether they worked independently, and if applicable, details of automation tools used in the process. | Methods |
| Effect measures | 12 | Specify for each outcome the effect measure(s) (e.g. risk ratio, mean difference) used in the synthesis or presentation of results. | Methods |
| Synthesis methods | 13a | Describe the processes used to decide which studies were eligible for each synthesis (e.g. tabulating the study intervention characteristics and comparing against the planned groups for each synthesis (item #5)). | Methods |
|  | 13b | Describe any methods required to prepare the data for presentation or synthesis, such as handling of missing summary statistics, or data conversions. | Methods |
|  | 13c | Describe any methods used to tabulate or visually display results of individual studies and syntheses. | Methods |
|  | 13d | Describe any methods used to synthesize results and provide a rationale for the choice(s). If meta-analysis was performed, describe the model(s), method(s) to identify the presence and extent of statistical heterogeneity, and software package(s) used. | Methods |
|  | 13e | Describe any methods used to explore possible causes of heterogeneity among study results (e.g. subgroup analysis, meta-regression). | Methods |
|  | 13f | Describe any sensitivity analyses conducted to assess robustness of the synthesized results. | Methods |
| Reporting bias assessment | 14 | Describe any methods used to assess risk of bias due to missing results in a synthesis (arising from reporting biases). | Methods |
| Certainty assessment | 15 | Describe any methods used to assess certainty (or confidence) in the body of evidence for an outcome. | Methods |
| **RESULTS** |  |  |  |
| Study selection | 16a | Describe the results of the search and selection process, from the number of records identified in the search to the number of studies included in the review, ideally using a flow diagram. | Results |
|  | 16b | Cite studies that might appear to meet the inclusion criteria, but which were excluded, and explain why they were excluded. | Results |
| Study characteristics | 17 | Cite each included study and present its characteristics. | Appendices |
| Risk of bias in studies | 18 | Present assessments of risk of bias for each included study. | Appendices |
| Results of individual studies | 19 | For all outcomes, present, for each study: (a) summary statistics for each group (where appropriate) and (b) an effect estimate and its precision (e.g. confidence/credible interval), ideally using structured tables or plots. | Appendices |
| Results of syntheses | 20a | For each synthesis, briefly summarise the characteristics and risk of bias among contributing studies. | Results |
|  | 20b | Present results of all statistical syntheses conducted. If meta-analysis was done, present for each the summary estimate and its precision (e.g. confidence/credible interval) and measures of statistical heterogeneity. If comparing groups, describe the direction of the effect. | Results and appendices |
|  | 20c | Present results of all investigations of possible causes of heterogeneity among study results. | Results and appendices |
|  | 20d | Present results of all sensitivity analyses conducted to assess the robustness of the synthesized results. | Results and appendices |
| Reporting biases | 21 | Present assessments of risk of bias due to missing results (arising from reporting biases) for each synthesis assessed. | Results and appendices |
| Certainty of evidence | 22 | Present assessments of certainty (or confidence) in the body of evidence for each outcome assessed. | Results |
| **DISCUSSION** |  |  |  |
| Discussion | 23a | Provide a general interpretation of the results in the context of other evidence. | Discussion |
|  | 23b | Discuss any limitations of the evidence included in the review. | Discussion |
|  | 23c | Discuss any limitations of the review processes used. | Discussion |
|  | 23d | Discuss implications of the results for practice, policy, and future research. | Discussion |
| **OTHER INFORMATION** | |  |  |
| Registration and protocol | 24a | Provide registration information for the review, including register name and registration number, or state that the review was not registered. | Methods |
|  | 24b | Indicate where the review protocol can be accessed, or state that a protocol was not prepared. | Methods |
|  | 24c | Describe and explain any amendments to information provided at registration or in the protocol. | NA |
| Support | 25 | Describe sources of financial or non-financial support for the review, and the role of the funders or sponsors in the review. | Title page |
| Competing interests | 26 | Declare any competing interests of review authors. | Title page |
| Availability of data, code, and other materials | 27 | Report which of the following are publicly available and where they can be found: template data collection forms; data extracted from included studies; data used for all analyses; analytic code; any other materials used in the review. | Methods |

# Appendix C. Search Strategy

Database search strategy to identify relevant studies

1. exp social stigma/
2. exp Stigma/
3. exp drug discrimination/
4. exp perceived discrimination/
5. exp prejudice/
6. exp Stereotyping/
7. exp social discrimination/
8. exp perceptive discrimination/
9. (stigma or discrimin* or "social stigma" or (social adj3 stigma) or stigm* or prejudice or stigma or discrimination or bias or intolerance or harass*).mp.
10. 1 or 2 or 3 or 4 or 5 or 6 or 7 or 8 or 9
11. exp obstetric violence/
12. exp workplace violence/
13. exp family violence/
14. exp partner violence/
15. exp sexual violence/
16. exp exposure to violence/
17. exp police violence/
18. exp physical violence/
19. exp domestic violence/
20. exp Gender-Based Violence/
21. exp gender based violence/
22. exp dating violence/
23. exp violence/
24. exp gun violence/
25. exp Ethnic Violence/
26. exp Intimate Partner Violence/
27. violence.mp.
28. 11 or 12 or 13 or 14 or 15 or 16 or 17 or 18 or 19 or 20 or 21 or 22 or 23 or 24 or 25 or 26 or 27
29. exp criminal law/
30. exp policy/
31. exp government regulation/
32. exp legal services/
33. exp liability, legal/
34. exp law enforcement/
35. exp legislation, drug/
36. exp social control, informal/
37. exp "health care quality, access, and evaluation"/
38. exp Police/
39. exp Law enforcement/
40. exp Legislation/
41. exp Crime/
42. exp Prisons/
43. (criminalis* or criminaliz* or prohibit* or decriminalis* or decriminaliz* or legalisation or legalization or legislat* or law* or legal* or illegal* or policing or police* or enforce* or criminal justice or prison* or penalt* or fine* or raid* or police operation* or arrest* or detention* or detain* or sanction* or dispersal order* or zoning restriction* or zon* or convict* or police record* or criminal record*).mp.
44. (anti-social behaviour* or (police adj3 harrass*) or (police adj3 repress*) or (police adj3 warning) or (police adj3 evict*) or (police adj3 engage$) or (police adj3 caution) or civil order* or administrative offense or (police adj3 coercion) or confiscat* or crime prevention or brothel closure* or confiscat* or loiter* or solicit* or support order or rehabilit* or (vagrancy adj3 act) or (municipal adj3 order) or by-law or police repress* or licens* or regulation or mandatory test* or registration or forced test* or condoms as evidence).mp.
45. 29 or 30 or 31 or 32 or 33 or 34 or 35 or 36 or 37 or 38 or 39 or 40 or 41 or 42 or 43 or 44
46. exp HIV Seroprevalence/
47. exp HIV-2/
48. exp HIV Infections/
49. exp HIV-1/
50. exp HIV Seropositivity/
51. exp HIV Testing/
52. exp HIV/
53. exp HIV Antibodies/
54. (HIV or "acquired immunodeficiency syndrome" or "AIDS" or "aids" or "hiv").mp.
55. 46 or 47 or 48 or 49 or 50 or 51 or 52 or 53 or 54
56. exp Sex Work/
57. ("sex work*" or prostitut* or "street walker*" or escort* or rent boy* or "sell sex" or "sold sex" or "selling sex" or "exchanged sex" or "exchange sex" or "exchanged sex" or "sex trade" or "commercial sex" or "sex industry" or "transactional sex" or "sexual favour" or "bar hostess" or "red light district" or "escort*").mp.
58. 56 or 57
59. exp Data Collection/ or Longitudinal Studies/ or longitudinal stud*.mp. or regression analysis/ or linear models/ or logistic models/ or proportional hazards models/ or spatial regression/ or survival analysis/ or epidemiologic studies/ or case-control studies/ or case control stud*.mp. or retrospective studies/ or cohort studies/ or follow-up studies/ or longitudinal studies/ or prospective studies/ or controlled before-after studies/ or cross-sectional studies/ or cross?sectional stud*.mp. or historically controlled study/ or interrupted time series analysis/ or seroepidemiologic studies/ or seroprevalence stud*.mp. or hiv seroprevalence/ or feasibility studies/ or multicenter studies as topic/ or pilot projects/ or sampling studies/ or sentinel surveillance/ or seroprevalence stud*.mp. or bio behavio?ral stud*.mp. or bio?behavio?ral stud*.mp. or health survey.mp. or exp Health Surveys/ or sampling studies.mp. or exp Sampling Studies/
60. 10 or 28 or 45
61. 55 and 58
62. 60 and 61
63. 59 and 62
64. limit 63 to yr="2010 -Current"
65. remove duplicates from 64

# Appendix D. Studies excluded because of duplicate data

Table 2. Studies excluded because of duplicate data

| **Lead author (year)** | **Title** | **Journal** |
| --- | --- | --- |
| Braunstein (2012) | Risk factor detection as a metric of STARHS performance for HIV incidence surveillance among female sex workers in Kigali, Rwanda | The open AIDS journal |
| Kerrigan (2019) | Project Shikamana: Community empowerment-based Combination HIV Prevention Significantly Impacts HIV Incidence and Care Continuum Outcomes among Female Sex Workers in Iringa, Tanzania | JAIDS Journal of Acquired Immune Deficiency Syndromes |
| Pickles (2024) | Exploratory analysis of the potential impact of violence on HIV among female sex workers in Mombasa, Kenya: a mathematical modelling study | BMC Medicine |
| Tounkara (2013) | Relationship between violence and HIV infection among female sex workers in Benin | Sexually Transmitted Infections |
| Mountain (2018) | Experience of violence is associated with HIV prevention uptake and HIV infection among young women who self-identify as sex workers in Mombasa, Kenya | HIV Research for Prevention 2018 |

# Appendix E. Studies identified through reference list searching

Table 3. Studies identified through reference list searching

| **Lead author (year)** | **Title** | **Journal** |
| --- | --- | --- |
| Amogne (2019) | Prevalence and correlates of physical violence and rape among female sex workers in Ethiopia: a Cross-sectional study with Respondent-driven sampling from 11 major towns | BMJ Open |
| Comins (2024) | ART coverage and viral suppression among female sex workers living with HIV in eThekwini, South Africa: Baseline findings from the Siyaphambili study | PLOS Global Public Health |
| Lima (2017) | Factors associated with violence against female sex workers in ten Brazilian cities | Cadernos de Saúde Pública |
| Mountain (2018) | Associations between violence, HIV prevention and HIV infection among young women who self-identify as sex workers in Mombasa, Kenya | PhD Dissertation |
| Prangnell (2018) | Workplace violence among female sex workers who use drugs in Vancouver, Canada: does client-targeted policing increase safety? | Journal of public health policy |
| Vu (2017) | High Burden of HIV, Syphilis and HSVNA2 and Factors Associated with HIV Infection Among Female Sex Workers in Tanzania: Implications for Early Treatment of HIV and Pre-exposure Prophylaxis (PrEP) | AIDS and Behavior |
| West (2022) | Typologies and Correlates of Police Violence Against Female Sex Workers Who Inject Drugs at the Mexico-United States Border: Limits of De Jure Decriminalization in Advancing Health and Human Rights | Journal of interpersonal violence |
| Wilson (2016b) | Prevalence and correlates of intimate partner violence in HIV-positive women engaged in transactional sex in Mombasa, Kenya | International journal of STD & AIDS |
| Wirth (2013) | How Does Sex Trafficking Increase the Risk of HIV Infection? An Observational Study From Southern India | American Journal of Epidemiology |

# Appendix F. Variables included in data extraction

Data was extracted on:

- Lead author, review title or unique identifier and date
- Publication type
- Study design
- Recruitment strategy
- Study location
- Study setting
- Study period (years in which recruitment occurred)
- Study sample size
- Study inclusion and exclusion criteria (including definition of PWID, e.g. injecting in the last 30 days)
- Definition of exposure
- Time frame of exposure
- Perpetrator of exposure
- Number and proportion of exposed participants
- Definition of outcome
- Unadjusted and adjusted effect size (e.g. incidence rate ratio (IRR); odds ratio (OR); hazard ratio (HR); and precision (e.g. 95% confidence interval (CI))
- Covariates included in adjusted models

# Appendix G. Study quality domains

Table 4. Study quality domains

| **Domain** | **Description** |
| --- | --- |
| Domain 1: Sample representativeness of the target population | This domain assessed the extent to which the study sample was likely to approximate the target population. One point was assigned if the study used recruitment approaches intended to enhance representativeness among KPs, including RDS, time-location or venue-based sampling, or recruitment through multiple, diverse sources (e.g., NSPs, shelters, street outreach and OAT clinics for the PWID review). Probability-based sampling, while rarely used, was also considered indicative of high representativeness. To receive one point, studies could not be restricted to narrowly defined subgroups (e.g., specific age groups, gender identities, or sexual orientations), unless such restrictions were intrinsic to the study population (e.g., all women in studies of FSW). Studies that did not meet these criteria were assigned zero points. |
| Domain 2: Temporality of exposure and outcome | This domain assessed whether stigma or violence exposure clearly preceded the HIV-related outcome of interest. One point was assigned to longitudinal studies in which exposure was measured prior to outcome assessment. Longitudinal studies that assessed exposure and outcome over the same time period, as well as cross-sectional studies, were assigned zero points. |
| Domain Three: Control for confounding | This domain assessed whether estimates were adjusted for potential confounders. One point was assigned if estimates were adjusted; unadjusted estimates were assigned zero points. |

# Appendix H. Definitions of forms of violence

Table 5. Definitions of forms of violence

| Societal barrier | Description |
| --- | --- |
| Physical violence | Acts of physical violence perpetrated against study participants, and experiences in which participants reported feeling physically threatened or endangered. |
| Sexual violence | Any sexual act, attempt to obtain a sexual act, or other act directed against a study participant sexuality using coercion, by any person regardless of their relationship, in any setting. It includes rape, defined as the physically forced or otherwise coerced penetration of the vulva or anus with a penis, other body part or object. |
| Physical and/or sexual violence | Acts of violence perpetrated against study participants, where the nature of the violence is not specified, or sexual violence and physical violence have been combined. |
| Other violence | Other forms of violence that do not constitute physical violence or sexual violence (for example, emotional violence). |

# Appendix I. Definitions of HIV outcomes

Table 6. Definitions of HIV outcomes

| HIV Outcome | Numerator | Denominator | Description |
| --- | --- | --- | --- |
| HIV infection | FSWs living with HIV | All FSWs | Measures the proportion of all FSWs in the community who are living with HIV. |
| HIV incidence | Incident HIV infections | Person-time at-risk of HIV or recency assay | Longitudinally measured using tests subsequent to an initial negative HIV test. Recency assays use biomarkers to detect new and longstanding HIV infections. |
| ART initiation | FSWs prescribed ART | FSWs newly diagnosed with HIV | FSWs who were recently diagnosed with HIV who received a prescription for ART and initiated treatment |
| ART use | FSWs living with HIV on ART | FSWs living with diagnosed HIV | FSWs who are living with HIV and are currently prescribed ART. |
| ART adherence | FSWs adhering to ART regimen | FSWs living with diagnosed HIV with ART prescription | The proportion of ART doses taken on time within a given time period. |
| HIV viral suppression | Virally suppressed FSWs living with HIV | FSWs living with diagnosed HIV or FSWs living with diagnosed HIV with ART prescription | A measure of HIV viral load, including both dichotomous indicators of viral suppression (viral load above or below a particular threshold), and continuous measures of viral load. |

# Appendix J. Variables extracted for subgroup analyses

Table 7. Variables extracted for subgroup analyses

| Variable | Description |
| --- | --- |
| WHO region | The WHO regions are divided into six geographic areas: Africa, Americas, South-East Asia, Europe, Eastern Mediterranean, and Western Pacific. |
| Country income levels | Dichotomised into two categories (lower-income and lower-middle income, upper-middle income and upper income) |
| Year of publication | Dichotomised into two categories (published between 2010–2016 and published between 2017–2025). |
| Perpetrator | Perpetrator of violence. Includes clients, employers, intimate partners, family or friends, police, and healthcare workers. |
| Recruitment method | Strategy used to identify, contact, and enrol participants. Includes respondent-driven sampling, time-space sampling, convenience sampling, and probabilistic sampling. |
| Study quality | Study quality was assessed on three criteria: representativeness, study design and confounder adjustment |

# Appendix K. Characteristics of included studies (n=94)

Table 8. Geographic and design characteristics of included studies

| **Author (year)** | **Publication type** | **Study design** | **Recruitment method** | **Country** | **WHO region** |
| --- | --- | --- | --- | --- | --- |
| Abdella (2022) (1) | Ethiopia | Manuscript | Cross-sectional | Respondent-driven | African Region |
| Aho (2013) (2) | Guinea | Manuscript | Cross-sectional | Convenience | African Region |
| Alaei (2021) (3) | Tajikistan | Manuscript | Cross-sectional | Respondent-driven | European Region |
| Alary (2014) (4) | India | Manuscript | Cross-sectional | Respondent-driven, cluster and time-space sampling | South-East Asia Region |
| Alemu (2022) (5) | Ethiopia | Manuscript | Cross-sectional | Probabilistic | African Region |
| Amogne (2019) (6) | Ethiopia | Manuscript | Cross-sectional | Respondent-driven | African Region |
| Arimide (2022) (7) | Ethiopia | Manuscript | Cross-sectional | Respondent-driven | African Region |
| Arumugam (2022) (8) | India | Manuscript | Cross-sectional | Probabilistic | South-East Asia Region |
| Ayamah (2023) (9) | Ghana | Manuscript | Cross-sectional | Time-space sampling | African Region |
| Beattie (2010) (10) | India | Manuscript | Cross-sectional | Time-space sampling | South-East Asia Region |
| Beattie (2015) (11) | India | Manuscript | Cross-sectional | Time-space sampling | South-East Asia Region |
| Beksinska (2018) (12) | India | Manuscript | Cross-sectional | Time-space sampling | South-East Asia Region |
| Berger (2018) (13) | Eswatini | Manuscript | Cross-sectional | Respondent-driven | African Region |
| Bhardwaj (2023) (14) | South Africa | Manuscript | Cross-sectional | Convenience | African Region |
| Bowring (2019) (15) | Cameroon | Manuscript | Cross-sectional | Respondent-driven | African Region |
| Braunstein (2011) (16) | Rwanda | Manuscript | Cross-sectional | Convenience | African Region |
| Budhwani (2017) (17) | Jamaica | Manuscript | Cross-sectional | Convenience | Region of the Americas |
| Budhwani (2021) (18) | Dominican Republic | Manuscript | Cross-sectional | Convenience | Region of the Americas |
| Bugssa (2015) (19) | Ethiopia | Manuscript | Cross-sectional | Probabilistic | African Region |
| Chabata (2019) (20) | Zimbabwe | Manuscript | Cross-sectional | Respondent-driven | African Region |
| Chirombo (2025) (21) | Malawi | Manuscript | Cross-sectional | Respondent-driven | African Region |
| Coetzee (2017) (22) | South Africa | Manuscript | Cross-sectional | Respondent-driven | African Region |
| Comins (2024) (23) | South Africa | Manuscript | Cross-sectional | Convenience | African Region |
| Damacena (2011) (24) | Brazil | Manuscript | Cross-sectional | Respondent-driven | Region of the Americas |
| Davey (2020) (25) | Zimbabwe | Manuscript | Cross-sectional | Respondent-driven | African Region |
| Decker (2012) (26) | Russia | Manuscript | Cross-sectional | Respondent-driven | European Region |
| Decker (2014) (27) | Russia | Manuscript | Cross-sectional | Respondent-driven | European Region |
| Decker (2016) (28) | Cameroon | Manuscript | Cross-sectional | Respondent-driven | African Region |
| Doshi (2018) (29) | Uganda | Manuscript | Cross-sectional | Respondent-driven | African Region |
| Duff (2016) (30) | Canada | Manuscript | Prospective cohort | Time-space sampling | Region of the Americas |
| Emmanuel (2021) (31) | Pakistan | Manuscript | Cross-sectional | Probabilistic | Eastern Mediterranean Region |
| Faini (2022) (32) | Tanzania | Manuscript | Cross-sectional | Respondent-driven | African Region |
| Goldenberg (2013) (33) | Mexico | Manuscript | Cross-sectional | Respondent-driven | Region of the Americas |
| Goldenberg (2016a) (34) | Uganda | Manuscript | Cross-sectional | Time-space sampling | African Region |
| Goldenberg (2016b) (35) | Canada | Manuscript | Prospective cohort | Time-space sampling | Region of the Americas |
| Goldenberg (2019) (36) | Uganda | Manuscript | Cross-sectional | Time-space sampling | African Region |
| Guure (2023) (37) | Ghana | Manuscript | Cross-sectional | Time-space sampling | African Region |
| Hendrickson (2018) (38) | Tanzania | Manuscript | Cross-sectional | Time-space sampling | African Region |
| Hensen (2019) (39) | Zimbabwe | Manuscript | Cross-sectional | Respondent-driven | African Region |
| Hladik (2017) (40) | Uganda | Manuscript | Cross-sectional | Respondent-driven | African Region |
| Iakunchykova (2017) (41) | Ukraine | Manuscript | Cross-sectional | Time-space sampling | European Region |
| Izadi (2023) (42) | Iran | Manuscript | Cross-sectional | Respondent-driven | Eastern Mediterranean Region |
| Jennings Mayo-Wilson (2023) (43) | Uganda | Manuscript | Cross-sectional | Time-space sampling | African Region |
| Jones (2023) (44) | Zimbabwe | Manuscript | Retrospective cohort | Convenience | African Region |
| Johnston (2025) (45) | Morocco | Manuscript | Cross-sectional | Respondent-driven | Eastern Mediterranean Region |
| Kakisingi (2020) (46) | Democratic Republic of the Congo | Manuscript | Cross-sectional | Convenience | African Region |
| Kassanjee (2022) (47) | South Africa | Manuscript | Cross-sectional | Convenience | African Region |
| Kelly-Hanku (2020) (48) | Papua New Guinea | Manuscript | Cross-sectional | Respondent-driven | Western Pacific Region |
| Kerrigan (2017) (49) | Tanzania | Manuscript | Cross-sectional | Time-space sampling | African Region |
| Kumar (2014) (50) | India | Manuscript | Cross-sectional | Respondent-driven | South-East Asia Region |
| Leddy (2018) (51) | Tanzania | Manuscript | Cross-sectional | Time-space sampling | African Region |
| Lima (2017) (52) | Brazil | Manuscript | Cross-sectional | Respondent-driven | Region of the Americas |
| Luiz (2025) (53) | Mozambique | Manuscript | Cross-sectional | Respondent-driven | African Region |
| Lyons (2017) (54) | Côte d’Ivoire | Manuscript | Cross-sectional | Respondent-driven | African Region |
| Lyons (2020) (55) | Multi-country | Manuscript | Cross-sectional | Respondent-driven | African Region |
| MacLin (2023) (56) | Dominican Republic | Manuscript | Cross-sectional | Convenience | Region of the Americas |
| Mendoza (2017) (57) | Dominican Republic | Manuscript | Cross-sectional | Snowball and peer-led | Region of the Americas |
| Mizinduko (2020) (58) | Tanzania | Manuscript | Cross-sectional | Respondent-driven | African Region |
| Mountain (2018) (59) | Kenya | PhD dissertation | Cross-sectional | Time-space sampling and cluster sampling | African Region |
| Mukherjee (2022) (60) | Kazakhstan | Manuscript | Cross-sectional | Convenience | European Region |
| Mukuku (2024) (61) | Democratic Republic of the Congo | Manuscript | Cross-sectional | Respondent-driven | African Region |
| Mulholland (2022) (62) | multi-country | Manuscript | Cross-sectional | Probabilistic | African Region |
| Mutagoma (2017) (63) | Rwanda | Manuscript | Cross-sectional | Time-space sampling | African Region |
| Mutagoma (2019) (64) | Rwanda | Manuscript | Cross-sectional | Time-space sampling | African Region |
| Nabayinda (2023) (65) | Uganda | Manuscript | Cross-sectional | Time-space sampling | African Region |
| Namale (2019) (66) | Uganda | Manuscript | Cross-sectional | Convenience | African Region |
| Oldenburg (2018) (67) | Zambia | Manuscript | Cross-sectional | Respondent-driven | African Region |
| Ouma (2021) (68) | Uganda | Manuscript | Cross-sectional | Convenience | African Region |
| Pando (2013) (69) | Argentina | Manuscript | Cross-sectional | Convenience | Region of the Americas |
| Prangnell (2018) (70) | Canada | Manuscript | Cross-sectional | Convenience | Region of the Americas |
| Ramesh (2012) (71) | India | Manuscript | Cross-sectional | Time-space sampling and cluster sampling | South-East Asia Region |
| Rice (2022) (72) | Zimbabwe | Manuscript | Cross-sectional | Respondent-driven | African Region |
| Schwartz (2016) (73) | South Africa | Manuscript | Cross-sectional | Respondent-driven | African Region |
| Sherman (2019) (74) | USA | Manuscript | Cross-sectional | Time-space sampling | Region of the Americas |
| Shokoohi (2017) (75) | Iran | Manuscript | Cross-sectional | Convenience | Eastern Mediterranean Region |
| Shrestha (2017) (76) | Nepal | Manuscript | Cross-sectional | Probabilistic | South-East Asia Region |
| Sileo (2018) (77) | Uganda | Manuscript | Cross-sectional | Respondent-driven | African Region |
| Strathdee (2011) (78) | Mexico | Manuscript | Cross-sectional | Respondent-driven | Region of the Americas |
| Szwarcwald (2017) (79) | Brazil | Manuscript | Cross-sectional | Respondent-driven | Region of the Americas |
| Tokar (2013) (80) | Ukraine | Conference abstract | Cross-sectional | Respondent-driven and time-space sampling | European Region |
| Tounkara (2014) (81) | Benin | Manuscript | Cross-sectional | Probabilistic | African Region |
| Tuot (2020) (82) | Cambodia | Manuscript | Cross-sectional | Respondent-driven | South-East Asia Region |
| Ulibarri (2011) (83) | Mexico | Manuscript | Cross-sectional | Respondent-driven | Region of the Americas |
| Vandenhoudt (2013) (84) | Kenya | Manuscript | Cross-sectional | Respondent-driven | African Region |
| Vu (2017) (85) | Tanzania | Manuscript | Cross-sectional | Respondent-driven | African Region |
| Wand (2015) (86) | Papua New Guinea | Manuscript | Cross-sectional | Respondent-driven | South-East Asia Region |
| Wang (2021) (87) | Malaysia | Manuscript | Cross-sectional | Respondent-driven | South-East Asia Region |
| West (2022) (88) | Mexico | Manuscript | Cross-sectional | Convenience | Region of the Americas |
| Wilson (2016a) (89) | Kenya | Manuscript | Prospective cohort | Convenience | African Region |
| Wilson (2016b) (90) | Kenya | Manuscript | Cross-sectional | Convenience | African Region |
| Wirth (2013) (91) | India | Manuscript | Cross-sectional | Convenience | South-East Asia Region |
| Xie (2023) (92) | China | Manuscript | Cross-sectional | Convenience | South-East Asia Region |
| Zalla (2019) (93) | Haiti | Manuscript | Cross-sectional | Probabilistic | Region of the Americas |
| Zhang (2012) (94) | China | Manuscript | Cross-sectional | Convenience | South-East Asia Region |

Table 9. Sex work and HIV characteristics of included studies

| **Author (year)** | **Country** | **Overall sample size** | **Analytic sample size** | **Number HIV+** | **Percent HIV+** |
| --- | --- | --- | --- | --- | --- |
| Abdella (2022) (1) | Ethiopia | 6085 | 6085 | 1138 | 19% |
| Aho (2013) (2) | Guinea | 223 | 221 | 78 | 35% |
| Alaei (2021) (3) | Tajikistan | 2174 | 2174 | 64 | 3% |
| Alary (2014) (4) | India | 23176 | 21707 | 3385 | 16% |
| Alemu (2022) (5) | Ethiopia | 381 | 381 | 76 | 20% |
| Amogne (2019) (6) | Ethiopia | 4900 | 4900 | 1173 | 24% |
| Arimide (2022) (7) | Ethiopia | 4900 | 239 | 239 | 100% |
| Arumugam (2022) (8) | India | 27007 | 27007 | 548 | 2% |
| Ayamah (2023) (9) | Ghana | 4279 | 3493 | 222 | 6% |
| Beattie (2010) (10) | India | 3852 | 3852 | 634 | 16% |
| Beattie (2015) (11) | India | 5792 | 3909 | 495 | 13% |
| Beksinska (2018) (12) | India | 1111 | 1111 | 91 | 8% |
| Berger (2018) (13) | Eswatini | 325 | 325 | 222 | 70% |
| Bhardwaj (2023) (14) | South Africa | 1384 | 1384 | 1384 | 100% |
| Bowring (2019) (15) | Cameroon | 2255 | 2255 | 550 | 24% |
| Braunstein (2011) (16) | Rwanda | 800 | 629 | 192 | 24% |
| Budhwani (2017) (17) | Jamaica | 459 | 459 | 6 | 1% |
| Budhwani (2021) (18) | Dominican Republic | 307 | 307 | 110 | 36% |
| Bugssa (2015) (19) | Ethiopia | 319 | 319 | 38 | 12% |
| Chabata (2019) (20) | Zimbabwe | 503 | 502 | 173 | 34% |
| Chirombo (2025) (21) | Malawi | 223 | 223 | 161 | 74% |
| Coetzee (2017) (22) | South Africa | 508 | 508 | 280 | 55% |
| Comins (2024) (23) | South Africa | 1373 | 1373 | 1373 | 100% |
| Damacena (2011) (24) | Brazil | 2523 | 2523 | 121 | 5% |
| Davey (2020) (25) | Zimbabwe | 2839 | 2839 | 1671 | 59% |
| Decker (2012) (26) | Russia | 147 | 143 | 7 | 5% |
| Decker (2014) (27) | Russia | 754 | 754 | 29 | 4% |
| Decker (2016) (28) | Cameroon | 1817 | 81 | 81 | 100% |
| Doshi (2018) (29) | Uganda | 1497 | 1497 | 485 | 31% |
| Duff (2016) (30) | Canada | 72 | 72 | 72 | 100% |
| Emmanuel (2021) (31) | Pakistan | 5660 | 5660 | 121 | 2% |
| Faini (2022) (32) | Tanzania | 773 | 773 | 59 | 8% |
| Goldenberg (2013) (33) | Mexico | 214 | 214 | 9 | 4% |
| Goldenberg (2016a) (34) | Uganda | 400 | 400 | 135 | 34% |
| Goldenberg (2016b) (35) | Canada | 74 | 74 | 74 | 100% |
| Goldenberg (2019) (36) | Uganda | 400 | 89 | 89 | 100% |
| Guure (2023) (37) | Ghana | 5990 | 5679 | 264 | 5% |
| Hendrickson (2018) (38) | Tanzania | 496 | 496 | 203 | 41% |
| Hensen (2019) (39) | Zimbabwe | 2387 | 2387 | 543 | 24% |
| Hladik (2017) (40) | Uganda | 942 | 942 | 323 | 33% |
| Iakunchykova (2017) (41) | Ukraine | 4806 | 4806 | 270 | 6% |
| Izadi (2023) (42) | Iran | 1515 | 1515 | 24 | 2% |
| Jennings Mayo-Wilson (2023) (43) | Uganda | 542 | 542 | 192 | 35% |
| Johnston (2025) (45) | Morocco | 4530 | 4098 | 78 | 2% |
| Jones (2023) (44) | Zimbabwe | 6665 | 6665 | NR | NR |
| Kakisingi (2020) (46) | Democratic Republic of the Congo | 1555 | 1555 | 127 | 8% |
| Kassanjee (2022) (47) | South Africa | 3005 | 2999 | 1862 | 62% |
| Kelly-Hanku (2020) (48) | Papua New Guinea | 2091 | 848 | 83 | 10% |
| Kerrigan (2017) (49) | Tanzania | 496 | 62 | 62 | 100% |
| Kumar (2014) (50) | India | 4074 | 3873 | 551 | 14% |
| Leddy (2018) (51) | Tanzania | 496 | 496 | 203 | 41% |
| Lima (2017) (52) | Brazil | 2523 | 2523 | NR | NR |
| Luiz (2025) (53) | Mozambique | 2567 | 2565 | 676 | 26% |
| Lyons (2017) (54) | Côte d’Ivoire | 466 | 453 | 50 | 11% |
| Lyons (2020) (55) | Multi-country | 7259 | 7259 | 2070 | 29% |
| MacLin (2023) (56) | Dominican Republic | 311 | 211 | 211 | 100% |
| Mendoza (2017) (57) | Dominican Republic | 268 | 268 | 268 | 100% |
| Mizinduko (2020) (58) | Tanzania | 958 | 958 | 169 | 18% |
| Mountain (2018) (59) | Kenya | 408 | 346 | 34 | 10% |
| Mukherjee (2022) (60) | Kazakhstan | 255 | 255 | 64 | 25% |
| Mukuku (2024) (61) | Democratic Republic of the Congo | 572 | 572 | 19 | 3% |
| Mulholland (2022) (62) | multi-country | 786 | 715 | 85 | 12% |
| Mutagoma (2017) (63) | Rwanda | 1338 | 1112 | 565 | 51% |
| Mutagoma (2019) (64) | Rwanda | 1978 | 1978 | 819 | 42% |
| Nabayinda (2023) (65) | Uganda | 542 | 542 | 220 | 41% |
| Namale (2019) (66) | Uganda | 603 | 432 | 432 | 100% |
| Oldenburg (2018) (67) | Zambia | 964 | 898 | 234 | 24% |
| Ouma (2021) (68) | Uganda | 300 | 300 | 126 | 42% |
| Pando (2013) (69) | Argentina | 1255 | 870 | NR | NR |
| Prangnell (2018) (70) | Canada | 259 | 259 | 89 | 34% |
| Ramesh (2012) (71) | India | 2042 | 2042 | 371 | 18% |
| Rice (2022) (72) | Zimbabwe | 605 | 605 | 122 | 20% |
| Schwartz (2016) (73) | South Africa | 410 | 163 | 163 | 100% |
| Sherman (2019) (74) | USA | 262 | 249 | 13 | 5% |
| Shokoohi (2017) (75) | Iran | 1337 | 1295 | 0 | 0% |
| Shrestha (2017) (76) | Nepal | 610 | 610 | 6 | 1% |
| Sileo (2018) (77) | Uganda | 115 | 115 | NR | NR |
| Strathdee (2011) (78) | Mexico | 620 | 620 | 33 | 5% |
| Szwarcwald (2017) (79) | Brazil | 4245 | 4245 | 225 | 5% |
| Tokar (2013) (80) | Ukraine | 5023 | 5023 | NR | NR |
| Tounkara (2014) (81) | Benin | 981 | 981 | 200 | 20% |
| Tuot (2020) (82) | Cambodia | 3149 | 3149 | 72 | 3% |
| Ulibarri (2011) (83) | Mexico | 924 | 924 | 55 | 6% |
| Vandenhoudt (2013) (84) | Kenya | 481 | 481 | 277 | 58% |
| Vu (2017) (85) | Tanzania | 1914 | 1906 | 535 | 28% |
| Wand (2015) (86) | Papua New Guinea | 523 | 203 | 14 | 7% |
| Wang (2021) (87) | Malaysia | 299 | 283 | 17 | 6% |
| West (2022) (88) | Mexico | 584 | 584 | 0 | 0% |
| Wilson (2016a) (89) | Kenya | 195 | 195 | 195 | 100% |
| Wilson (2016b) (90) | Kenya | 357 | 356 | 357 | 100% |
| Wirth (2013) (91) | India | 1814 | 1814 | 310 | 18% |
| Xie (2023) (92) | China | 480 | 480 | NR | NR |
| Zalla (2019) (93) | Haiti | 990 | 958 | 75 | 8% |
| Zhang (2012) (94) | China | 1022 | 937 | NR | NR |

# Appendix L. Estimates of the association between sexual violence and HIV outcomes

Table 10. Estimates of the association between sexual violence and HIV outcomes

| **Author (year)** | **Violence definition** | **Violence time frame** | **Exposed, n (%)** | **Perpetrator** | **HIV outcome** | **Effect size (95% CI)** | **Variables adjusted for** |
| --- | --- | --- | --- | --- | --- | --- | --- |
| Alemu (2022) | Sexual assault | Ever | 44 (12%) | Unspecified | HIV prevalence | OR: 1.60 (0.78–3.28); aOR: 1.59 (0.76–3.33) | Age, condom use, condom breakage, sex during menstrual cycle, place of sex |
| Amogne (2019) | Rape | Ever | 742 (15%) | Client or sexual partner | HIV prevalence | OR: 0.88 (0.73–1.07) | NA |
| Arimide (2022) | Forced into selling sex | Ever | 26 (11%) | Unspecified | Viral suppression | OR: 0.33 (0.14–0.76); aOR: 0.36 (0.14–0.93) | Number sexual partners per month, CD4 count, age |
| Arumugam (2022) | Forced sexual intercourse | Past 12 months | 4847 (18%) | Unspecified | HIV prevalence | OR: 0.96 (0.77–1.20) | NA |
| Ayamah (2023) | Sexual violence | Past 12 months | 1059 (25%) | Unspecified | HIV prevalence | OR: 0.94 (0.69–1.29) | NA |
| Ayamah (2023) | Sexual violence | Past 12 months | 1059 (25%) | Unspecified | HIV testing | OR: 0.91 (0.77–1.07) | NA |
| Bowring (2019) | Forced to have sex against will | Past 6 months | 202 (10%) | Unspecified | HIV prevalence | OR: 1.06 (0.69–1.61) | NA |
| Braunstein (2011) | History of forced sex | Ever | 202 (25%) | Unspecified | HIV incidence | OR: 2.26 (0.92–5.58); aOR: 2.30 (0.90–5.80) | Age and district of residence |
| Braunstein (2011) | History of forced sex | Ever | 202 (25%) | Unspecified | HIV prevalence | OR: 2.21 (1.55–3.13); aOR: 2.20 (1.50–3.20) | Age and district of residence |
| Budhwani (2017) | Sexually abused | Ever | 82 (24%) | Unspecified | HIV prevalence | OR: 1.07 (0.11–10.43) | NR |
| Bugssa (2015) | Sexually abused | Ever | 44 (14%) | Unspecified | HIV prevalence | OR: 3.10 (1.40–6.70); aOR: 2.20 (1.40–6.30) | NA |
| Chabata (2019) | Forced to have sexual intercourse | Ever | 44 (9%) | Unspecified | HIV prevalence | OR: 1.32 (0.57–3.06) | NA |
| Coetzee (2017) | Sexual violence by non-intimate partner | Ever | 277 (55%) | Unspecified | HIV prevalence | OR: 1.64 (1.15–2.33) | NA |
| Comins (2024) | Forced to have sex | Ever | 515 (38%) | Unspecified | Viral suppression | OR: 0.71 (0.59–0.85); aOR: 0.87 (0.71–1.08) | NA |
| Damacena (2011) | Physically forced sex | Ever | NR | Unspecified | HIV prevalence | OR: 1.74 (1.00–3.03) | NA |
| Decker (2014) | Forced or coerced vaginal sex | Past 6 months | 86 (11%) | Client | HIV prevalence | OR: 3.79 (1.67–8.61); aOR: 3.77 (1.73–8.22) | Length of time in sex work, age, site stratification survey design accommodated through complex survey analyses |
| Decker (2014) | Sex to avoid incarceration or arrest | Past 6 months | 23 (3%) | Police | HIV prevalence | OR: 4.07 (1.14–14.56); aOR: 3.45 (0.92–12.93) | Length of time in sex work, age, site stratification survey design accommodated through complex survey analyses |
| Decker (2014) | Forced or coerced anal sex | Past 6 months | 47 (6%) | Client | HIV prevalence | OR: 5.45 (2.20–13.51); aOR: 4.80 (1.89–12.19) | Length of time in sex work, age, site stratification survey design accommodated through complex survey analyses |
| Decker (2012) | Sex for leniency from police | Past 3 months | 52 (37%) | Police | HIV prevalence | OR: 2.42 (0.52–11.25) | Sociodemographic factors |
| Decker (2012) | Sexual violence | Past 12 months | 5 (4%) | Pimp | HIV prevalence | OR: 1.84 (0.09–36.95) | NA |
| Doshi (2018) | Forced into sex work | Ever | 71 (33%) | Unspecified | Current ART | OR: 0.98 (0.40–2.43) | NA |
| Doshi (2018) | Forced into sex work | Ever | 222 (65%) | Unspecified | Viral suppression | OR: 0.67 (0.38–1.20) | NA |
| Faini (2022) | Rape | Past 3 months | 142 (18%) | Unspecified | HIV prevalence | OR: 2.19 (1.21–3.95) | NA |
| Goldenberg (2013) | Involuntary sex exchange | Past month | 31 (14%) | Client | HIV prevalence | OR: 1.73 (0.34–8.76) | NA |
| Guure (2023) | Forced sex | Ever | 986 (15%) | Unspecified | HIV prevalence | OR: 1.26 (0.91–1.74); aOR: 1.38 (1.02–1.89) | Adjusted for variables that were statistically significant on bivariate analysis (not reported) |
| Hensen (2019) | Experienced sexual violence | Past 12 months | 390 (16%) | Unspecified | HIV prevalence | OR: 1.20 (0.86–1.68) | NA |
| Hladik (2017) | Raped | Past month | 243 (26%) | Client | HIV prevalence | OR: 1.03 (0.76–1.41) | NA |
| Hladik (2017) | Raped | Past month | 308 (33%) | Unspecified | HIV prevalence | OR: 1.10 (0.82–1.46) | NA |
| Izadi (2023) | Violent sex | Ever | 600 (40%) | Unspecified | HIV prevalence | OR: 1.81 (0.80–4.06) | NA |
| Kakisingi (2020) | Sexual violence due to selling sex | Past 3 months | 32 (2%) | Unspecified | HIV prevalence | OR: 1.17 (0.35–3.89); aOR: 0.74 (0.21–2.62) | Age, number of partners, sex work as main income, years selling sex, condom use, number of sexual intercourses, alcohol use, STIs, type of sexual intercourse |
| Kassanjee (2022) | Sexual violence | Past 12 months | 1447 (48%) | Client | HIV incidence | OR: 1.42 (0.04–52.04) | NA |
| Kelly-Hanku (2020) | Sexual violence | Past 12 months | 109 (16%) | Unspecified | HIV prevalence | OR: 2.80 (1.30–6.10) | NA |
| Luiz (2025) | Sexual violence related to their profession | Past 6 months | 975 (38%) | Unspecified | HIV prevalence | OR: 1.00 (0.83–1.20) | NA |
| Lyons (2017) | Forced sex | Ever | 195 (43%) | Unspecified | HIV prevalence | OR: 0.99 (0.54–1.80) | NA |
| Lyons (2017) | Forced sex | Ever | 200 (43%) | Unspecified | HIV testing | OR: 1.27 (0.78–2.06) | NA |
| Lyons (2020) | Forced sex | Ever | 2207 (30%) | Unspecified | HIV prevalence | OR: 1.29 (1.16–1.44); aOR: 1.32 (1.13–1.54) | Age, education level, marital status, years in sex work, country-level epidemic, and clustered by site and by country. Adjusted for disclosure of sex work to family of healthcare provider |
| Mizinduko (2020) | Forced sex | Past 12 months | 312 (33%) | Unspecified | HIV prevalence | OR: 1.45 (0.98–2.15); aOR: 1.94 (1.34–2.82) | Age, education, marital status, current living status, age at first sold sex, payment received when last sold sex, forced sex status in the past one year, primary meeting point with clients, and reason for first sold sex. |
| Mountain (2018) | Forced sex | Ever | 119 (30%) | Unspecified | HIV prevalence | OR: 1.56 (0.89–2.72); aOR: 1.44 (0.81–2.56) | Age, regular income |
| Mountain (2018) | Forced sex | Ever | 119 (30%) | Unspecified | HIV testing | OR: 0.81 (0.66–1.00); aOR: 0.82 (0.66–1.01) | Age, regular income |
| Mukuku (2024) | Forced sex | Past 12 months | 29 (5%) | Unspecified | HIV prevalence | OR: 10.60 (3.70–30.50); aOR: 12.20 (3.20–46.40) | Age, duration in sex work, number of clients, anal sex, number of sexual intercourses, condom use, history of STI, alcohol consumption |
| Mulholland (2022) | Forced sex | Ever | 158 (20%) | Unspecified | HIV prevalence | OR: 1.13 (0.64–2.00) | NA |
| Mutagoma (2019) | Sexual violence | Ever | 361 (18%) | Unspecified | HIV prevalence | OR: 1.40 (1.28–1.57) | NA |
| Oldenburg (2018) | Sexual intimate partner violence | Past 12 months | 30 (47%) | Intimate partner | ART initiation | aOR: 0.42 (0.22–0.77) | Age, literacy, income, primary partner, site, study arm, education, mobile phone ownership, age at sexual debut, time since HIV diagnosis, age at initiation of sex work, number of clients on an average night |
| Pando (2013) | Sexual abuse during lifetime | Ever | 182 (20%) | Unspecified | HIV prevalence | OR: 2.40 (0.90–6.60); aOR: 1.50 (0.90–2.50) | Nationality (Argentinean vs. other), formal education level (primary or less vs. high school or more), age and place of work (street vs. other). |
| Pando (2013) | Forced first sex or sexual abuse in lifetime | Ever | 219 (24%) | Unspecified | HIV prevalence | OR: 2.10 (0.80–5.80); aOR: 1.40 (0.80–2.40) | Nationality (Argentinean vs. other), formal education level (primary or less vs. high school or more), age and place of work (street vs. other). |
| Rice (2022) | Sexual violence/abuse | Past 12 months | 124 (20%) | Unspecified | HIV prevalence | OR: 1.40 (0.80–2.45) | NA |
| Sherman (2019) | Childhood sexual abuse (CGW) | Ever | NR | Family or friend | HIV prevalence | OR: 0.57 (0.18–1.80) | NA |
| Sherman (2019) | Childhood sexual abuse (TGW) | Ever | NR | Family or friend | HIV prevalence | OR: 3.39 (2.73–4.21); aOR: 4.56 (1.20–17.32) | Age, drug use, schooling, employment, risk environment |
| Shokoohi (2017) | Sexual violence | Ever | 512 (40%) | Unspecified | HIV testing | OR: 1.09 (0.97–1.22); aOR: 1.02 (0.91–1.14) | Type of sample, incarceration history, HIV knowledge, number of partners, condom use, harm reduction use, health service utilisation, knowing a site for HIV testing |
| Shrestha (2017) | Forced sex | Past 12 months | 125 (20%) | Unspecified | HIV testing | OR: 1.99 (1.32–3.01); aOR: 1.10 (1.00–1.30) | Having dependents, injecting drugs, participated in HIV programme, depression |
| Sileo (2018) | Sexual violence | Ever | 71 (62%) | Intimate partner | HIV prevalence | OR: 3.33 (1.11–10.03); aOR: 3.94 (1.22–12.66) | NA |
| Sileo (2018) | Sexual violence | Past 12 months | 62 (54%) | Intimate partner | HIV prevalence | OR: 1.67 (0.66–4.21) | NA |
| Strathdee (2011) | Sexually abused as a child | Ever | 205 (33%) | Family or friend | HIV prevalence | OR: 0.77 (0.35–1.69) | NA |
| Strathdee (2011) | Sexually abused/raped | Past month | 34 (6%) | Intimate partner | HIV prevalence | OR: 1.13 (0.26–4.95) | NA |
| Strathdee (2011) | Sexually abused/raped | Past month | 138 (23%) | Client | HIV prevalence | OR: 1.10 (0.48–2.49) | NA |
| Szwarcwald (2017) | Sexual violence | Ever | NR | Unspecified | HIV prevalence | OR: 1.54 (1.05–2.26) | NA |
| Tounkara (2014) | Forced sex | Past month | 132 (14%) | Unspecified | HIV prevalence | OR: 1.56 (1.03–2.37); aOR: 1.42 (1.02–1.98) | Experienced violence, sex work typology, duration of sex work, literacy, number of clients, regular partners, gonorrhoea infection, antecedent for STI, condom use |
| Tuot (2020) | Gang rape | Past 3 months | 40 (1%) | Unspecified | HIV prevalence | OR: 4.48 (1.72–11.70) | NA |
| Tuot (2020) | Forced sex | Past 3 months | 42 (1%) | Unspecified | HIV prevalence | OR: 5.28 (2.17–12.84) | NA |
| Ulibarri (2011) | Sexual abuse | Past 6 months | 88 (10%) | Client | HIV prevalence | OR: 3.50 (1.66–7.36); aOR: 3.00 (1.30–6.92) | Injecting drug use |
| Vandenhoudt (2013) | Raped as FSW | Ever | 193 (40%) | Unspecified | HIV prevalence | OR: 0.91 (0.63–1.32); aOR: 0.90 (0.60–1.30) | Age adjusted OR |
| Vu (2017) | Sexually abused | Past 12 months | 485 (25%) | Client | HIV prevalence | OR: 1.10 (0.80–1.50) | NA |
| Wand (2015) | Raped | Past 12 months | 74 (14%) | Unspecified | HIV prevalence | OR: 3.48 (1.08–11.21); aOR: 3.42 (1.06–11.06) | Age, education, age at sexual debut, Cohabitation status, total client volume, alcohol use, anal/oral sex, condom use, HIV knowledge, STI diagnosis ever and current, syphilis diagnosis |
| Wang (2021) | Childhood sexual assault (CWSW) | Ever | 77 (27%) | Unspecified | HIV prevalence | OR: 1.12 (0.38–3.28) | NA |
| Wang (2021) | Adulthood sexual assault (TGWSW) | Ever | 44 (24%) | Unspecified | HIV prevalence | OR: 3.26 (1.17–9.05); aOR: 4.06 (0.68–24.19) | NA |
| Wang (2021) | Childhood sexual assault (TGWSW) | Ever | 89 (48%) | Unspecified | HIV prevalence | OR: 3.98 (1.25–12.17); aOR: 0.92 (0.12–6.75) | NA |
| West (2022) | Material violence and sexual violence | Past 6 months | 92 (16%) | Police | HIV testing | OR: 1.68 (1.02–2.74) | NA |
| Wirth (2013) | Forced prostitution | Ever | 107 (6%) | Unspecified | HIV prevalence | OR: 2.74 (1.41–5.31); aOR: 2.30 (1.08–4.90) | NA |
| Zalla (2019) | Forced to have sex | Ever | 384 (40%) | Unspecified | HIV prevalence | OR: 2.07 (0.32–13.45) | NA |
| Zhang (2012) | Unwanted sexual intercourse | Ever | 135 (14%) | Client | HIV testing | OR: 0.65 (0.45–0.95) | NA |
| Zhang (2012) | Unwanted sexual intercourse | Ever | 120 (16%) | Intimate partner | HIV testing | OR: 0.59 (0.39–0.88) | NA |

# Appendix M. Estimates of the association between physical violence and HIV outcomes

Table 11. Estimates of the association between physical violence and HIV outcomes

| **Author (year)** | **Violence definition** | **Violence time frame** | **Exposed, n (%)** | **Perpetrator** | **HIV outcome** | **Effect size (95% CI)** | **Variables adjusted for** |
| --- | --- | --- | --- | --- | --- | --- | --- |
| Abdella (2022) | Physical violence | Past 12 months | 1356 (22%) | Unspecified | HIV prevalence | OR: 1.06 (0.91–1.24); aOR: 1.00 (0.80–1.10) | Age, age at first sex, marital status, education, income, condom breakage, lubricant use, history of STI, Treponema Pallidum test, alcohol use |
| Amogne (2019) | Physical beating | Past 12 months | 855 (18%) | Client or sexual partner | HIV prevalence | OR: 1.04 (0.88–1.24) | NA |
| Arimide (2022) | Physically beaten | Past 12 months | 30 (13%) | Unspecified | Viral suppression | OR: 0.88 (0.37–2.13) | NA |
| Arumugam (2022) | Physically beaten | Past 12 months | 6500 (24%) | Unspecified | HIV prevalence | OR: 1.01 (0.83–1.23) | NA |
| Ayamah (2023) | Physical violence | Past 12 months | 433 (10%) | Unspecified | HIV prevalence | OR: 1.00 (0.64–1.54) | NA |
| Ayamah (2023) | Physical violence | Past 12 months | 433 (10%) | Unspecified | HIV testing | OR: 0.91 (0.72–1.14) | NA |
| Bowring (2019) | Physical harassment/hurt due to sex work | Past 6 months | 232 (12%) | Unspecified | HIV prevalence | OR: 0.65 (0.41–1.03) | NA |
| Chabata (2019) | Physical violence | Ever | 146 (29%) | Client | HIV prevalence | OR: 1.08 (0.54–1.77) | NA |
| Chabata (2019) | Physical violence | Ever | 233 (46%) | Intimate partner | HIV prevalence | OR: 0.97 (0.58–1.63) | NA |
| Comins (2024) | Physical violence | Ever | 740 (54%) | Unspecified | Viral suppression | OR: 0.79 (0.66–0.93); aOR: 0.99 (0.82–1.20) | NA |
| Decker (2014) | Physical violence | Ever | 74 (18%) | Pimp | HIV prevalence | OR: 9.97 (2.92–34.07); aOR: 6.32 (1.85–21.63) | Length of time in sex work, age, site stratification survey design accommodated through complex survey analyses |
| Decker (2014) | Physical violence | Past 6 months | 77 (10%) | Client | HIV prevalence | OR: 2.98 (1.23–7.22); aOR: 2.52 (1.41–4.51) | Length of time in sex work, age, site stratification survey design accommodated through complex survey analyses |
| Decker (2012) | Threats of physical violence | Past 12 months | 14 (10%) | Pimp | HIV prevalence | OR: 16.36 (2.47–108.62) | Sociodemographic factors |
| Decker (2012) | Physical violence | Past 12 months | 107 (76%) | Client | HIV prevalence | OR: 0.78 (0.15–4.24) | Sociodemographic factors |
| Decker (2012) | Physical violence | Past 12 months | 12 (8%) | Pimp | HIV prevalence | OR: 0.68 (0.04–12.62) | NA |
| Goldenberg (2019) | Intimate partner violence | Past 6 months | 47 (53%) | Intimate partner | Current ART | OR: 0.80 (0.35–1.84) | NA |
| Hensen (2019) | Physical violence | Ever | 972 (39%) | Intimate partner | HIV prevalence | OR: 1.44 (1.12–1.86); aOR: 1.37 (1.05–1.78) | Age, education, marital status, sex work history, recent sexual behaviours |
| Kelly-Hanku (2020) | Physical violence | Past 12 months | 146 (17%) | Unspecified | HIV prevalence | OR: 2.30 (1.20–4.20); aOR: 2.50 (1.20–4.90) | Sex work as source of income, anal sex, years selling sex, used internet to meet clients, main male partner, contact with outreach worker, can count on other sex workers to borrow money, given free condoms, residence, casual male partner, STI symptoms |
| Lima (2017) | Physical violence | Past 12 months | 295 (12%) | Clients | HIV prevalence | OR: 1.79 (1.04–3.08) | NA |
| Lima (2017) | Physical violence | Past 12 months | 419 (17%) | Family member | HIV prevalence | OR: 1.41 (0.86–2.29) | NA |
| Lima (2017) | Physical violence | Past 12 months | 636 (25%) | Intimate partner | HIV prevalence | OR: 1.36 (0.94–1.97) | NA |
| Lima (2017) | Physical violence | Past 12 months | 961 (38%) | Any perpetrator | HIV prevalence | OR: 1.26 (0.97–1.64) | NA |
| Lima (2017) | Physical violence | Past 12 months | 199 (8%) | Police | HIV prevalence | OR: 1.82 (1.05–3.16) | NA |
| Luiz (2025) | Physical violence related to their profession | Past 6 months | 1341 (52%) | Unspecified | HIV prevalence | OR: 1.15 (0.97–1.37) | NA |
| Lyons (2017) | Physically hurt | Ever | 240 (53%) | Unspecified | HIV prevalence | OR: 1.83 (1.00–3.41) | NA |
| Lyons (2017) | Physically hurt | Ever | 247 (53%) | Unspecified | HIV testing | OR: 1.02 (0.54–1.65) | NA |
| Lyons (2020) | Physical violence | Ever | 2359 (33%) | Unspecified | HIV prevalence | OR: 1.58 (1.42–1.76); aOR: 1.23 (1.02–1.49) | Age, education level, marital status, years in sex work, country-level epidemic, and clustered by site and by country. Adjusted for disclosure of sex work to family of healthcare provider |
| Mountain (2018) | Physical hurt by sex partner | Ever | 123 (30%) | Sexual partner | HIV prevalence | OR: 2.04 (1.27–4.27); aOR: 1.89 (1.15–4.10) | Age, regular income |
| Mountain (2018) | Police assault or arrest | Ever | 183 (45%) | Police | HIV prevalence | OR: 1.61 (0.81–4.20); aOR: 1.61 (0.80–4.21) | Age, ever married, duration in sex work |
| Mountain (2018) | Physical hurt by sex partner | Ever | 123 (30%) | Sexual partner | HIV testing | OR: 0.83 (0.68–1.01); aOR: 0.84 (0.68–1.03) | Age, regular income |
| Mountain (2018) | Police assault or arrest | Ever | 183 (45%) | Police | HIV testing | OR: 0.85 (0.69–1.04); aOR: 0.84 (0.68–1.04) | Age, ever married, duration in sex work |
| Mulholland (2022) | Physical violence | Ever | 250 (32%) | Intimate partner | HIV prevalence | OR: 1.03 (0.62–1.71) | NA |
| Mutagoma (2019) | Physical violence | Ever | 702 (36%) | Unspecified | HIV prevalence | OR: 1.50 (1.11–2.05) | NA |
| Namale (2019) | Physical violence from sexual partner(s) | Past 3 months | 148 (34%) | Intimate partner | Viral suppression | OR: 0.80 (0.40–1.60) | NA |
| Oldenburg (2018) | Physical intimate partner violence | Past 12 months | 25 (50%) | Intimate partner | ART initiation | aOR: 0.65 (0.35–1.21) | Age, literacy, income, primary partner, site, study arm, education, mobile phone ownership, age at sexual debut, time since HIV diagnosis, age at initiation of sex work, number of clients on an average night |
| Pando (2013) | Beaten because of sex work | Ever | 267 (22%) | Unspecified | HIV prevalence | OR: 1.90 (0.80–4.50); aOR: 1.20 (0.80–1.90) | Nationality (Argentinean vs. other), formal education level (primary or less vs. high school or more), age and place of work (street vs. other). |
| Rice (2022) | Physical violence/abuse | Past 12 months | 133 (22%) | Client | HIV prevalence | OR: 2.53 (1.48–4.32) | NA |
| Rice (2022) | Physical violence/abuse | Past 12 months | 151 (25%) | Intimate partner | HIV prevalence | OR: 1.51 (0.89–2.58); aOR: 1.97 (1.05–3.67) | Age, site, in school, education level, marital status, place of residence, sex work debut |
| Rice (2022) | Physical violence | Past 12 months | 50 (8%) | Police | HIV prevalence | OR: 1.23 (0.52–2.92) | NA |
| Schwartz (2016) | Physical abuse/violence | Past 12 months | NR | Unspecified | Current ART | OR: 0.84 (0.64–1.10); aOR: 0.87 (0.67–1.13) | Adjusted model controls for variables statistically significant at p<0.20 in univariate analyses and time since HIV diagnosis. |
| Sherman (2019) | Childhood physical abuse | Ever | NR | Family or friend | HIV prevalence | OR: 1.17 (0.39–3.53) | NA |
| Shrestha (2017) | Physical assault | Past 12 months | 81 (13%) | Unspecified | HIV testing | OR: 1.56 (0.98–2.50); aOR: 0.80 (0.60–1.00) | Having dependents, injecting drugs, participated in HIV programme, depression |
| Sileo (2018) | Physical violence | Ever | 59 (51%) | Intimate partner | HIV prevalence | OR: 1.16 (0.47–2.87) | NA |
| Sileo (2018) | Physical violence | Past 12 months | 46 (40%) | Intimate partner | HIV prevalence | OR: 1.05 (0.43–2.56) | NA |
| Strathdee (2011) | Physically abused as a child | Ever | 150 (25%) | Family or friend | HIV prevalence | OR: 0.85 (0.36–2.02) | NA |
| Szwarcwald (2017) | Physical violence | Ever | NR | Unspecified | HIV prevalence | OR: 1.08 (0.73–1.61) | NA |
| Tounkara (2014) | Physical violence | Past month | 168 (17%) | Unspecified | HIV prevalence | OR: 1.32 (0.89–1.96); aOR: 1.45 (1.05–2.00) | Experienced violence, sex work typology, duration of sex work, literacy, number of clients, regular partners, gonorrhoea infection, antecedent for STI, condom use |
| Vu (2017) | Physically abused | Past 12 months | 656 (34%) | Client | HIV prevalence | OR: 0.80 (0.60–1.10) | NA |
| Wang (2021) | Adulthood physical assault (CWSW) | Ever | 99 (35%) | Unspecified | HIV prevalence | OR: 2.22 (0.83–5.96) | NA |
| Wang (2021) | Childhood physical assault (CWSW) | Ever | 101 (36%) | Unspecified | HIV prevalence | OR: 1.68 (0.63–4.50) | NA |
| Wang (2021) | Physical assault because of sex work (CWSW) | Ever | 26 (9%) | Unspecified | HIV prevalence | OR: 4.80 (1.54–14.93); aOR: 2.59 (0.67–10.19) | NA |
| Wang (2021) | Physical assault because of sex work (TGWSW) | Ever | 37 (20%) | Unspecified | HIV prevalence | OR: 1.27 (0.39–4.14) | NA |
| Wang (2021) | Adulthood physical assault (TGWSW) | Ever | 61 (33%) | Unspecified | HIV prevalence | OR: 0.61 (0.19–1.94) | NA |
| Wang (2021) | Childhood physical assault (TGWSW) | Ever | 74 (40%) | Unspecified | HIV prevalence | OR: 1.80 (0.66–4.90) | NA |
| Zhang (2012) | Physically hurt | Ever | 126 (17%) | Client | HIV testing | OR: 1.13 (0.77–1.65) | NA |
| Zhang (2012) | Physically hurt | Ever | 148 (20%) | Intimate partner | HIV testing | OR: 0.80 (0.56–1.15) | NA |

# Appendix N. Estimates of the association between mixed violence and HIV outcomes

Table 12. Estimates of the association between mixed violence and HIV outcomes

| **Author (year)** | **Violence definition** | **Violence time frame** | **Exposed, n (%)** | **Perpetrator** | **HIV outcome** | **Effect size (95% CI)** | **Variables adjusted for** |
| --- | --- | --- | --- | --- | --- | --- | --- |
| Aho (2013) | Violence episode | Past 3 months | 53 (24%) | Client | HIV prevalence | OR: 1.17 (0.62–2.22) | NA |
| Alary (2014) | Experienced violence | Ever | 2190 (10%) | Unspecified | HIV prevalence | aOR: 1.35 (1.19–1.53) | IBBA round, age, literacy, income source, marital status, age at first sex, duration in sex work, condom use, sex work typology, partner status, STI status, baseline HIV prevalence |
| Beattie (2015) | Beaten or forced to have sex | Past 12 months | 416 (11%) | Unspecified | HIV prevalence | OR: 1.87 (1.40–2.49); aOR: 1.59 (1.18–2.15) | District, IBBA round, place where sex worker has sex with clients, age, marital status, additional source of income to sex work and ever sold sex outside the district (migrant sex worker) |
| Beattie (2015) | Beaten or forced to have sex | Past 12 months | 416 (11%) | Unspecified | HIV testing | OR: 1.02 (0.78–1.33); aOR: 1.09 (0.81–1.48) | District, IBBA round, place where sex worker has sex with clients, age, marital status, additional source of income to sex work and ever sold sex outside the district (migrant sex worker) |
| Beattie (2010) | Beaten or forced to have sex | Past 12 months | 413 (11%) | Unspecified | HIV prevalence | OR: 1.10 (0.80–1.49); aOR: 0.96 (0.70–1.32) | Survey round, age, marital status, residency, migrant, duration sex work, place entertain clients, clients per week and regular partner. |
| Beksinska (2018) | Domestic violence | Past 12 months | 216 (19%) | Intimate partner | HIV prevalence | OR: 0.28 (0.11–0.73); aOR: 0.40 (0.15–1.09) | Age, district, marital status, migrant sex work, place of selling sex and having an income other than sex work |
| Beksinska (2018) | Domestic and workplace/community violence | Past 12 months | 69 (6%) | Unspecified | HIV prevalence | OR: 1.11 (0.40–3.38); aOR: 1.32 (0.41–4.29) | Age, district, marital status, migrant sex work, place of selling sex and having an income other than sex work |
| Beksinska (2018) | Workplace or community violence | Past 12 months | 80 (7%) | Unspecified | HIV prevalence | OR: 1.75 (0.88–3.50); aOR: 1.16 (0.55–2.44) | Age, district, marital status, migrant sex work, place of selling sex and having an income other than sex work |
| Beksinska (2018) | Any violence | Past 12 months | 365 (33%) | Unspecified | HIV prevalence | OR: 0.73 (0.42–1.27); aOR: 0.82 (0.44–1.53) | Age, district, marital status, migrant sex work, place of selling sex and having an income other than sex work |
| Berger (2018) | Beaten or forced to have sex | Ever | 156.816 (48%) | Unspecified | HIV prevalence | OR: 1.09 (0.67–1.78) | NA |
| Berger (2018) | Beaten or forced to have sex | Ever | 133 (41%) | Unspecified | HIV testing | OR: 1.15 (0.70–1.91) | NA |
| Bhardwaj (2023) | Experienced physical or sexual violence and moderate to severe drug use risk | Ever | 638 (46%) | Unspecified | Viral suppression | aOR: 0.88 (0.79–0.99) | Age (as a cubic term), homelessness, relationship status, and anticipated stigma were used to estimate adjusted prevalence ratios (aPRs) |
| Budhwani (2021) | Beaten or raped in past year | Past 12 months | 64 (22%) | Unspecified | HIV prevalence | aOR: 3.15 (1.19–8.34) | NA |
| Chiromobo (2025) | Verbal, sexual, and physical abuse | Ever | 118 (54%) | Client or police | HIV prevalence | OR: 0.78 (0.42–1.44) | NA |
| Coetzee (2017) | Intimate partner physical/sexual violence | Ever | 288 (57%) | Intimate partner | HIV prevalence | OR: 0.95 (0.66–1.35) | NA |
| Davey (2020) | Physically hurt or raped | Past month | 637 (23%) | Any | Current ART | OR: 0.80 (0.55–1.15); aOR: 0.96 (0.90–1.03) | NA |
| Davey (2020) | Physically hurt | Past month | 637 (23%) | Unspecified | HIV testing | OR: 1.07 (0.80–1.43); aOR: 0.99 (0.87–1.12) | NA |
| Davey (2020) | Physically hurt or raped | Past month | 637 (23%) | Unspecified | Viral suppression | OR: 0.87 (0.59–1.28); aOR: 1.00 (0.93–1.07) | NA |
| Decker (2016) | Beaten up, physically hurt or forced to have sex | Ever | 1098 (60%) | Unspecified | Current ART | OR: 0.59 (0.20–1.72); aOR: 0.49 (0.13–1.82) | Age, marital status, education, city, income other than sex work and number of clients in the past month. |
| Decker (2016) | Beaten or forced to have sex | Ever | 1098 (60%) | Unspecified | HIV testing | OR: 0.83 (0.66–1.05); aOR: 0.92 (0.71–1.19) | Age, marital status, education, city, income other than sex work and number of clients in the past month. |
| Duff (2016) | Physical/sexual violence | Past 6 months | 13 (18%) | Client | Viral suppression | OR: 0.50 (0.20–1.23) | NA |
| Emmanuel (2021) | Faced violence | Past 12 months | NR | Unspecified | HIV prevalence | OR: 1.10 (0.70–1.50) | NA |
| Goldenberg (2019) | Sexual/physical abuse | Past 6 months | 39 (44%) | Client | Current ART | OR: 0.66 (0.28–1.53) | NA |
| Goldenberg (2016) | Physical/sexual violence | Past 6 months | 10 (14%) | Client | ART adherence | OR: 0.71 (0.17–3.03) | NA |
| Goldenberg (2016) | Verbal, physical, or sexual violence | Past 6 months | 37 (9%) | Police | HIV prevalence | OR: 1.07 (0.53–2.18) | NA |
| Goldenberg (2016) | Verbal, physical, or sexual violence | Past 6 months | 66 (16%) | Intimate partner | HIV prevalence | OR: 0.83 (0.47–1.47) | NA |
| Goldenberg (2016) | Physical or sexual violence | Past 6 months | 314 (78%) | Client | HIV prevalence | OR: 0.82 (0.50–1.35) | NA |
| Hendrickson (2018) | Physical or sexual GBV | Past 6 months | 197 (40%) | Unspecified | HIV prevalence | OR: 1.15 (0.82–1.61); aOR: 1.37 (0.92–2.04) | Work-related mobility, general mobility, age, income, number of clients, sex work-related stigma, alcohol use, community (Mafinga vs Ilula) |
| Hladik (2017) | Violence due to selling sex | Ever | 433 (46%) | Unspecified | HIV prevalence | OR: 1.34 (1.02–1.75) | NA |
| Iakunchykova (2017) | Any violence during sex work | Ever | NR | Unspecified | HIV prevalence | OR: 1.91 (1.48–2.47); aOR: 1.45 (1.09–1.93) | Place of solicitation, age, region, number of clients, alcohol use, injection drug use, income |
| Johnston (2025) | Forced first sex | Ever | NR | Unspecified | HIV testing | aOR: 1.39 (1.13–2.41) | City, year, age, marital status, education, number of partners, condom use, injection drug use, arrest, HIV knowledge |
| Johnston (2025) | Forced first sex | Ever | NR | Unspecified | HIV testing | aOR: 1.43 (1.16–2.37) | City, year, age, marital status, education, number of partners, condom use, injection drug use, arrest, HIV knowledge |
| Jones (2023) | NA | Ever | 1292 (20%) | Any perpretator | HIV incidence | OR: 1.02 (0.81–1.27); aOR: 1.08 (0.87–1.35) | Age, mean time between HIV test |
| Kerrigan (2017) | Gender-based violence | Ever | 252 (51%) | Unspecified | HIV prevalence | OR: 1.18 (0.80–1.73) | NA |
| Kerrigan (2017) | Gender-based violence | Ever | 39 (68%) | Unspecified | Viral suppression | OR: 0.59 (0.20–1.79) | NA |
| Kumar (2014) | Violence or forced sex | Past 12 months | 960 (25%) | Unspecified | HIV prevalence | OR: 1.74 (1.42–2.14); aOR: 1.76 (1.43–2.16) | District level intercept |
| Leddy (2018) | Physical or sexual GBV | Past 6 months | 197 (40%) | Unspecified | HIV prevalence | OR: 1.65 (0.95–2.86) | NA |
| MacLin (2023) | SW-related violence and harassment | Past 6 months | 18 (18%) | Unspecified | ART adherence | OR: 3.12 (0.36–25.00); aOR: 2.44 (0.27–20.00) | NA |
| MacLin (2023) | SW-related violence and harassment | Past 6 months | 18 (18%) | Unspecified | ART adherence | OR: 0.91 (0.29–2.88); aOR: 0.82 (0.26–2.64) | NA |
| MacLin (2023) | SW-related violence and harassment | Past 6 months | 18 (18%) | Unspecified | Current ART | OR: 1.25 (0.30–5.27); aOR: 1.27 (0.30–5.48) | NA |
| MacLin (2023) | SW-related violence and harassment | Past 6 months | 18 (18%) | Unspecified | Viral suppression | OR: 1.43 (0.43–4.76); aOR: 1.56 (0.46–5.23) | NA |
| Mendoza (2017) | Violence | Past 6 months | 33 (12%) | Intimate partner | ART adherence | OR: 0.27 (0.12–0.64); aOR: 0.19 (0.07–0.52) | Age, civil status, education, number of children, current residence, type of sex work venue, years in sex work, average price per date, alcohol use, and drug use |
| Mendoza (2017) | Violence | Past 6 months | 49 (18%) | Sexual partner | ART adherence | OR: 0.29 (0.14–0.60); aOR: 0.27 (0.11–0.63) | Age, civil status, education, number of children, current residence, type of sex work venue, years in sex work, average price per date, alcohol use, and drug use |
| Mendoza (2017) | Violence | Past 6 months | 23 (8%) | Client | ART adherence | OR: 0.37 (0.13–1.09); aOR: 0.47 (0.14–1.52) | Age, civil status, education, number of children, current residence, type of sex work venue, years in sex work, average price per date, alcohol use, and drug use |
| Mendoza (2017) | Violence | Past 6 months | 33 (12%) | Intimate partner | ART coverage | OR: 0.48 (0.21–1.11); aOR: 0.45 (0.17–1.18) | Age, civil status, education, number of children, current residence, type of sex work venue, years in sex work, average price per date, alcohol use, and drug use |
| Mendoza (2017) | Violence | Past 6 months | 49 (18%) | Sexual partner | ART coverage | OR: 0.31 (0.15–0.65); aOR: 0.31 (0.14–0.71) | Age, civil status, education, number of children, current residence, type of sex work venue, years in sex work, average price per date, alcohol use, and drug use |
| Mendoza (2017) | Violence | Past 6 months | 23 (8%) | Client | ART coverage | OR: 0.18 (0.05–0.67); aOR: 0.18 (0.05–0.67) | Age, civil status, education, number of children, current residence, type of sex work venue, years in sex work, average price per date, alcohol use, and drug use |
| Mendoza (2017) | Violence | Past 6 months | 33 (12%) | Intimate partner | ART coverage | OR: 0.83 (0.35–1.96); aOR: 0.94 (0.38–2.38) | Age, civil status, education, number of children, current residence, type of sex work venue, years in sex work, average price per date, alcohol use, and drug use |
| Mendoza (2017) | Violence | Past 6 months | 49 (18%) | Sexual partner | ART coverage | OR: 0.80 (0.39–1.67); aOR: 0.90 (0.41–2.00) | Age, civil status, education, number of children, current residence, type of sex work venue, years in sex work, average price per date, alcohol use, and drug use |
| Mendoza (2017) | Violence | Past 6 months | 23 (8%) | Client | ART coverage | OR: 0.47 (0.19–1.18); aOR: 0.47 (0.18–1.27) | Age, civil status, education, number of children, current residence, type of sex work venue, years in sex work, average price per date, alcohol use, and drug use |
| Mendoza (2017) | Violence | Past 6 months | 33 (12%) | Intimate partner | Current ART | OR: 0.25 (0.08–0.80); aOR: 0.25 (0.06–1.00) | Age, civil status, education, number of children, current residence, type of sex work venue, years in sex work, average price per date, alcohol use, and drug use |
| Mendoza (2017) | Violence | Past 6 months | 49 (18%) | Sexual partner | Current ART | OR: 0.32 (0.11–0.93); aOR: 0.41 (0.12–1.41) | Age, civil status, education, number of children, current residence, type of sex work venue, years in sex work, average price per date, alcohol use, and drug use |
| Mendoza (2017) | Violence | Past 6 months | 23 (8%) | Client | Current ART | OR: 0.86 (0.11–6.97); aOR: 0.40 (0.04–3.90) | Age, civil status, education, number of children, current residence, type of sex work venue, years in sex work, average price per date, alcohol use, and drug use |
| Mountain (2018) | Forced sex, physical violence and police assault or arrest | Ever | 44 (11%) | Unspecified | HIV prevalence | OR: 2.51 (1.11–5.64); aOR: 2.65 (1.19–5.90) | Age, age at entry into sex work |
| Mountain (2018) | Forced sex, physical violence and police assault or arrest | Ever | 44 (11%) | Unspecified | HIV testing | OR: 0.56 (0.37–0.84); aOR: 0.56 (0.37–0.85) | Age, age at entry into sex work |
| Mutagoma (2017) | Violence/harassment | Ever | 905 (51%) | Unspecified | HIV prevalence | OR: 1.00 (0.80–1.20) | NA |
| Nabayinda (2023) | Intimate-partner violence | Past 3 months | 248 (46%) | Intimate partner | HIV prevalence | OR: 0.96 (0.68–1.35) | NA |
| Oldenburg (2018) | Physical or sexual intimate partner violence | Past 12 months | 142 (61%) | Intimate partner | ART initiation | aOR: 0.40 (0.22–0.72) | Age, literacy, income, primary partner, site, study arm, education, mobile phone ownership, age at sexual debut, time since HIV diagnosis, age at initiation of sex work, number of clients on an average night |
| Ouma (2021) | Gender-based violence | Ever | 183 (61%) | Client | HIV prevalence | OR: 1.72 (1.07–2.79) | NR |
| Pando (2013) | Three violent experiences (vs. none) | Ever | 151 (11%) | Unspecified | HIV prevalence | OR: 5.50 (0.50–60.40); aOR: 1.20 (0.30–4.20) | Nationality (Argentinean vs. other), formal education level (primary or less vs. high school or more), age and place of work (street vs. other). |
| Pando (2013) | Two violent experiences (vs. none) | Ever | 330 (21%) | Unspecified | HIV prevalence | OR: 5.90 (0.70–52.20); aOR: 1.30 (0.50–3.40) | Nationality (Argentinean vs. other), formal education level (primary or less vs. high school or more), age and place of work (street vs. other). |
| Pando (2013) | One violent experience (vs. none) | Ever | 607 (32%) | Unspecified | HIV prevalence | OR: 3.80 (0.40–33.80); aOR: 0.80 (0.30–2.30) | Nationality (Argentinean vs. other), formal education level (primary or less vs. high school or more), age and place of work (street vs. other). |
| Pando (2013) | Four or more violent experiences (vs. none) | Ever | 56 (6%) | Unspecified | HIV prevalence | OR: 14.10 (1.50–133.00); aOR: 2.90 (0.90–9.20) | Nationality (Argentinean vs. other), formal education level (primary or less vs. high school or more), age and place of work (street vs. other). |
| Prangnell (2018) | Physical, sexual, or verbal violence | Past 6 months | 32 (12%) | Client | HIV prevalence | OR: 0.62 (0.27–1.46) | NA |
| Ramesh (2012) | Physically beaten or forced to have sex | Past 12 months | 352 (17%) | Unspecified | HIV prevalence | OR: 1.72 (1.31–2.26); aOR: 1.58 (1.20–2.09) | Age, marital status, literacy, alcohol consumption, duration of sex work, primary place of entertaining clients, client volume per week, having a regular male partner and experience of sexual violence in past year/mobility for sex work in past year. |
| Tokar (2013) | Personal experience of violence | NA | NR | Police | HIV prevalence | OR: 1.40 (1.00–1.80) | NA |
| Tokar (2013) | Personal experience of violence | NA | NR | Intimate partner | HIV prevalence | OR: 1.80 (1.30–2.60) | NA |
| Tokar (2013) | Personal experience of violence | NA | NR | Unspecified | HIV prevalence | OR: 1.80 (1.50–2.10) | NA |
| Wilson (2016) | Intimate-partner violence | Past 12 months | 45 (23%) | Intimate partner | ART adherence | OR: 0.51 (0.18–1.45); aOR: 0.63 (0.21–1.92) | NA |
| Wilson (2016) | Intimate-partner violence | Past 12 months | 45 (23%) | Intimate partner | ART adherence | OR: 1.18 (0.91–1.52); aOR: 1.19 (0.88–1.64) | NA |
| Wilson (2016) | Intimate-partner violence | Past 12 months | 45 (23%) | Intimate partner | Viral suppression | OR: 4.42 (1.36–14.38); aOR: 4.76 (1.19–20.00) | NA |
| Wilson (2016) | Intimate-partner violence | Past 12 months | 52 (15%) | Intimate partner | Current ART | OR: 0.86 (0.58–1.53) | NA |
| Xie (2023) | Violence | Ever | 65 (14%) | Client | HIV testing | OR: 2.83 (1.65–4.84) | NA |
| Zalla (2019) | Intimate-partner violence | Past 12 months | 305 (32%) | Intimate partner | HIV prevalence | OR: 3.72 (0.36–38.21) | NA |
| Zhang (2012) | Any type of violence | Ever | 422 (45%) | Client | HIV testing | OR: 1.05 (0.81–1.36); aOR: 0.98 (0.75–1.28) | Inconsistent condom use, inconsistent condom use in the last three sex acts, and intention of inconsistent condom use, history of STI, HIV testing, and drug use |
| Zhang (2012) | Any type of violence | Ever | 430 (58%) | Intimate partner | HIV testing | OR: 0.80 (0.59–1.07); aOR: 0.80 (0.59–1.09) | Inconsistent condom use, inconsistent condom use in the last three sex acts, and intention of inconsistent condom use, history of STI, HIV testing, and drug use |

# Appendix O. Estimates of the association between other forms of violence and HIV outcomes

Table 13. Estimates of the association between other forms of violence and HIV outcomes

| **Author (year)** | **Violence definition** | **Violence time frame** | **Exposed, n (%)** | **Perpetrator** | **HIV outcome** | **Effect size (95% CI)** | **Variables adjusted for** |
| --- | --- | --- | --- | --- | --- | --- | --- |
| Alaei (2021) | Experience of violence, stigma or discrimination | Past 12 months | 1317 (55%) | Unspecified | HIV prevalence | OR: 0.61 (0.37–1.00) | NA |
| Decker (2012) | Presented with more clients than agreed | Ever | 63 (44%) | Unspecified | HIV prevalence | OR: 0.95 (0.20–4.41) | NA |
| Goldenberg (2016) | Abducted into the Lord's Resistance Army | Past 6 months | 129 (32%) | Army | HIV prevalence | OR: 1.61 (1.04–2.49); aOR: 1.62 (1.00–2.63) | Educational attainment: none or incomplete primary school, age at first sex work, and incarceration |
| Jennings Mayo-Wilson (2023) | Reported economic abuse (high vs. low) | Ever | 175 (32%) | Unspecified | ART initiation | OR: 0.26 (0.03–2.52); aOR: 0.21 (0.02–2.22) | Age, education, partnership status |
| Jennings Mayo-Wilson (2023) | Reported economic abuse (medium vs. low) | Ever | 178 (33%) | Unspecified | ART initiation | OR: 0.37 (0.03–4.20); aOR: 0.44 (0.03–5.78) | Age, education, partnership status |
| Jennings Mayo-Wilson (2023) | Reported economic abuse (high vs. low) | Ever | 175 (32%) | Unspecified | HIV testing | OR: 1.34 (0.61–2.93); aOR: 1.29 (0.58–2.90) | Age, education, partnership status |
| Jennings Mayo-Wilson (2023) | Reported economic abuse (medium vs. low) | Ever | 178 (33%) | Unspecified | HIV testing | OR: 1.66 (0.73–3.75); aOR: 1.74 (0.76–4.01) | Age, education, partnership status |
| MacLin (2023) | SW-related police harassment | Past 6 months | 26 (12%) | Police | ART adherence | OR: 0.50 (0.21–1.18); aOR: 0.46 (0.19–1.14) | NA |
| MacLin (2023) | SW-related police harassment | Past 6 months | 26 (12%) | Police | ART adherence | OR: 0.78 (0.33–1.83); aOR: 0.71 (0.29–1.72) | NA |
| MacLin (2023) | SW-related police harassment | Past 6 months | 37 (37%) | Police | ART adherence | OR: 1.18 (0.34–4.08); aOR: 1.39 (0.38–3.85) | NA |
| MacLin (2023) | SW-related police harassment | Past 6 months | 37 (37%) | Police | ART adherence | OR: 2.66 (1.08–6.51); aOR: 2.78 (1.12–6.88) | NA |
| MacLin (2023) | SW-related police harassment | Past 6 months | 26 (12%) | Police | Current ART | OR: 0.98 (0.11–8.33); aOR: 0.99 (0.11–8.59) | NA |
| MacLin (2023) | SW-related police harassment | Past 6 months | 37 (37%) | Police | Current ART | OR: 2.06 (0.58–7.74); aOR: 2.05 (0.58–7.31) | NA |
| MacLin (2023) | SW-related police harassment | Past 6 months | 26 (12%) | Police | Viral suppression | OR: 0.85 (0.33–2.15); aOR: 0.82 (0.32–2.15) | NA |
| MacLin (2023) | SW-related police harassment | Past 6 months | 37 (37%) | Police | Viral suppression | OR: 0.81 (0.33–1.98); aOR: 0.79 (0.32–1.95) | NA |
| Mukherjee (2022) | Extortion & Discrimination (vs. low victimisation) | Past 3 months | 39 (15%) | Police | HIV prevalence | OR: 0.67 (0.27–1.66); aOR: 0.66 (0.26–1.68) | Age, educational attainment, and marital status |
| Mukherjee (2022) | Poly-Victimization (vs. low victimisation) | Past 3 months | 86 (34%) | Police | HIV prevalence | OR: 1.26 (0.68–2.32); aOR: 1.26 (0.68–2.37) | Age, educational attainment, and marital status |
| Sileo (2018) | Any emotional violence | Ever | 77 (67%) | Unspecified | HIV prevalence | OR: 2.86 (0.95–8.64) | NA |
| Sileo (2018) | Any emotional intimate partner violence | Past 12 months | 67 (58%) | Intimate partner | HIV prevalence | OR: 1.81 (0.70–4.65) | NA |
| Tounkara (2014) | Psychological violence | Past month | 326 (34%) | Unspecified | HIV prevalence | OR: 1.51 (1.10–2.08); aOR: 1.41 (1.08–1.85) | Experienced violence, sex work typology, duration of sex work, literacy, number of clients, regular partners, gonorrhea infection, antecedent for STI, condom use |
| West (2022) | Material violence, verbal and emotional harassment | Past 6 months | 279 (48%) | Police | HIV testing | OR: 2.42 (1.68–3.49) | NA |
| Zhang (2012) | Belittled, humiliated or threatened | Ever | 372 (40%) | Client | HIV testing | OR: 1.12 (0.87–1.46) | NA |
| Zhang (2012) | Belittled, humiliated or threatened | Ever | 411 (56%) | Intimate partner | HIV testing | OR: 0.77 (0.58–1.03) | NA |

# Appendix P. Study quality scores of included studies

Table 14. Study quality scores of included studies

| **Study** | **Domain 1: Sample representativeness** | **Domain 2: Temporality** | **Domain Three: Control for confounding** | **Total score /3** |
| --- | --- | --- | --- | --- |
| Abdella (2022) (1) | Random sampling | Cross-sectional | Adjusted | 2 |
| Aho (2013) (2) | Not random sampling | Cross-sectional | Unadjusted | 0 |
| Alaei (2021) (3) | Random sampling | Cross-sectional | Unadjusted | 1 |
| Alary (2014) (4) | Random sampling | Cross-sectional | Adjusted | 2 |
| Alemu (2022) (5) | Random sampling | Cross-sectional | Adjusted | 2 |
| Amogne (2019) (6) | Random sampling | Cross-sectional | Unadjusted | 1 |
| Arimide (2022) (7) | Random sampling | Cross-sectional | Adjusted | 2 |
| Arumugam (2022) (8) | Random sampling | Cross-sectional | Unadjusted | 1 |
| Ayamah (2023) (9) | Random sampling | Cross-sectional | Unadjusted | 1 |
| Beattie (2010) (10) | Random sampling | Cross-sectional | Adjusted | 2 |
| Beattie (2015) (11) | Random sampling | Cross-sectional | Adjusted | 2 |
| Beksinska (2018) (12) | Random sampling | Cross-sectional | Adjusted | 2 |
| Berger (2018) (13) | Random sampling | Cross-sectional | Unadjusted | 1 |
| Bhardwaj (2023) (14) | Not random sampling | Cross-sectional | Adjusted | 1 |
| Bowring (2019) (15) | Random sampling | Cross-sectional | Unadjusted | 1 |
| Braunstein (2011) (16) | Not random sampling | Cross-sectional | Adjusted | 1 |
| Budhwani (2017) (17) | Not random sampling | Cross-sectional | Unadjusted | 0 |
| Budhwani (2021) (18) | Not random sampling | Cross-sectional | Adjusted | 1 |
| Bugssa (2015) (19) | Random sampling | Cross-sectional | Adjusted | 2 |
| Chabata (2019) (20) | Random sampling | Cross-sectional | Unadjusted | 1 |
| Chirombo (2025) (21) | Random sampling | Cross-sectional | Unadjusted | 1 |
| Coetzee (2017) (22) | Random sampling | Cross-sectional | Unadjusted | 1 |
| Comins (2024) (23) | Not random sampling | Cross-sectional | Adjusted | 1 |
| Damacena (2011) (24) | Random sampling | Cross-sectional | Unadjusted | 1 |
| Davey (2020) (25) | Random sampling | Cross-sectional | Unadjusted | 1 |
| Decker (2012) (26) | Random sampling | Cross-sectional | Unadjusted | 1 |
| Decker (2014) (27) | Random sampling | Cross-sectional | Adjusted | 2 |
| Decker (2016) (28) | Random sampling | Cross-sectional | Adjusted | 2 |
| Doshi (2018) (29) | Random sampling | Cross-sectional | Unadjusted | 1 |
| Duff (2016) (30) | Random sampling | Longitudinal | Unadjusted | 2 |
| Emmanuel (2021) (31) | Random sampling | Cross-sectional | Unadjusted | 1 |
| Faini (2022) (32) | Random sampling | Cross-sectional | Unadjusted | 1 |
| Goldenberg (2013) (33) | Random sampling | Cross-sectional | Unadjusted | 1 |
| Goldenberg (2016a) (34) | Random sampling | Cross-sectional | Unadjusted | 1 |
| Goldenberg (2016b) (35) | Random sampling | Longitudinal | Unadjusted | 2 |
| Goldenberg (2019) (36) | Random sampling | Cross-sectional | Unadjusted | 1 |
| Guure (2023) (37) | Random sampling | Cross-sectional | Adjusted | 2 |
| Hendrickson (2018) (38) | Random sampling | Cross-sectional | Adjusted | 2 |
| Hensen (2019) (39) | Random sampling | Cross-sectional | Adjusted | 2 |
| Hladik (2017) (40) | Random sampling | Cross-sectional | Unadjusted | 1 |
| Iakunchykova (2017) (41) | Random sampling | Cross-sectional | Adjusted | 2 |
| Izadi (2023) (42) | Random sampling | Cross-sectional | Unadjusted | 1 |
| Jennings Mayo-Wilson (2023) (43) | Random sampling | Cross-sectional | Adjusted | 2 |
| Johnston (2025) (45) | Random sampling | Cross-sectional | Adjusted | 2 |
| Jones (2023) (44) | Not random sampling | Longitudinal | Adjusted | 2 |
| Kakisingi (2020) (46) | Not random sampling | Cross-sectional | Adjusted | 1 |
| Kassanjee (2022) (47) | Not random sampling | Cross-sectional | Unadjusted | 0 |
| Kelly-Hanku (2020) (48) | Random sampling | Cross-sectional | Adjusted | 2 |
| Kerrigan (2017) (49) | Random sampling | Cross-sectional | Unadjusted | 1 |
| Kumar (2014) (50) | Random sampling | Cross-sectional | Adjusted | 2 |
| Leddy (2018) (51) | Random sampling | Cross-sectional | Unadjusted | 1 |
| Lima (2017) (52) | Random sampling | Cross-sectional | Unadjusted | 1 |
| Luiz (2025) (53) | Random sampling | Cross-sectional | Unadjusted | 1 |
| Lyons (2017) (54) | Random sampling | Cross-sectional | Unadjusted | 1 |
| Lyons (2020) (55) | Random sampling | Cross-sectional | Adjusted | 2 |
| MacLin (2023) (56) | Not random sampling | Cross-sectional | Adjusted | 1 |
| Mendoza (2017) (57) | Not random sampling | Cross-sectional | Adjusted | 1 |
| Mizinduko (2020) (58) | Random sampling | Cross-sectional | Adjusted | 2 |
| Mountain (2018) (59) | Random sampling | Cross-sectional | Adjusted | 2 |
| Mukherjee (2022) (60) | Not random sampling | Cross-sectional | Adjusted | 1 |
| Mukuku (2024) (61) | Random sampling | Cross-sectional | Adjusted | 2 |
| Mulholland (2022) (62) | Random sampling | Cross-sectional | Unadjusted | 1 |
| Mutagoma (2017) (63) | Random sampling | Cross-sectional | Unadjusted | 1 |
| Mutagoma (2019) (64) | Random sampling | Cross-sectional | Unadjusted | 1 |
| Nabayinda (2023) (65) | Random sampling | Cross-sectional | Unadjusted | 1 |
| Namale (2019) (66) | Not random sampling | Cross-sectional | Unadjusted | 0 |
| Oldenburg (2018) (67) | Random sampling | Cross-sectional | Adjusted | 2 |
| Ouma (2021) (68) | Not random sampling | Cross-sectional | Adjusted | 1 |
| Pando (2013) (69) | Not random sampling | Cross-sectional | Adjusted | 1 |
| Prangnell (2018) (70) | Not random sampling | Cross-sectional | Unadjusted | 0 |
| Ramesh (2012) (71) | Random sampling | Cross-sectional | Adjusted | 2 |
| Rice (2022) (72) | Random sampling | Cross-sectional | Unadjusted | 1 |
| Schwartz (2016) (73) | Random sampling | Cross-sectional | Adjusted | 2 |
| Sherman (2019) (74) | Random sampling | Cross-sectional | Unadjusted | 1 |
| Shokoohi (2017) (75) | Not random sampling | Cross-sectional | Adjusted | 1 |
| Shrestha (2017) (76) | Random sampling | Cross-sectional | Adjusted | 2 |
| Sileo (2018) (77) | Random sampling | Cross-sectional | Unadjusted | 1 |
| Strathdee (2011) (78) | Random sampling | Cross-sectional | Unadjusted | 1 |
| Szwarcwald (2017) (79) | Random sampling | Cross-sectional | Unadjusted | 1 |
| Tokar (2013) (80) | Random sampling | Cross-sectional | Unadjusted | 1 |
| Tounkara (2014) (81) | Random sampling | Cross-sectional | Adjusted | 2 |
| Tuot (2020) (82) | Random sampling | Cross-sectional | Unadjusted | 1 |
| Ulibarri (2011) (83) | Random sampling | Cross-sectional | Adjusted | 2 |
| Vandenhoudt (2013) (84) | Random sampling | Cross-sectional | Adjusted | 2 |
| Vu (2017) (85) | Random sampling | Cross-sectional | Unadjusted | 1 |
| Wand (2015) (86) | Random sampling | Cross-sectional | Adjusted | 2 |
| Wang (2021) (87) | Random sampling | Cross-sectional | Unadjusted | 1 |
| West (2022) (88) | Not random sampling | Cross-sectional | Unadjusted | 0 |
| Wilson (2016a) (89) | Not random sampling | Longitudinal | Adjusted | 2 |
| Wilson (2016b) (90) | Not random sampling | Cross-sectional | Unadjusted | 0 |
| Wirth (2013) (91) | Not random sampling | Cross-sectional | Adjusted | 1 |
| Xie (2023) (92) | Not random sampling | Cross-sectional | Unadjusted | 0 |
| Zalla (2019) (93) | Random sampling | Cross-sectional | Unadjusted | 1 |
| Zhang (2012) (94) | Not random sampling | Cross-sectional | Adjusted | 1 |

# Appendix Q. Forest plots of all forms of violence estimates and prevalent HIV infection

Figure 1. Pooled unadjusted estimates of recent exposure to all forms of violence and prevalent HIV infection among female sex workers

Figure 2. Pooled unadjusted estimates of lifetime exposure to all forms of violence and prevalent HIV infection among female sex workers

Figure 3. Pooled adjusted estimates of recent exposure to all forms of violence and prevalent HIV infection among female sex workers

Figure 4. Pooled adjusted estimates of lifetime exposure to all forms of violence and prevalent HIV infection among female sex workers

Figure 5. Pooled combined unadjusted and adjusted estimates of recent exposure to all forms of violence and prevalent HIV infection among female sex workers

Figure 6. Pooled combined unadjusted and adjusted estimates of lifetime exposure to all forms of violence and prevalent HIV infection among female sex workers

# Appendix R. Forest plots of physical violence estimates and prevalent HIV infection

Figure 7. Pooled unadjusted estimates of recent exposure to physical violence and prevalent HIV infection among female sex workers

Figure 8. Pooled unadjusted estimates of lifetime exposure to physical violence and prevalent HIV infection among female sex workers

Figure 9. Pooled adjusted estimates of recent exposure to physical violence and prevalent HIV infection among female sex workers

Figure 10. Pooled adjusted estimates of lifetime exposure to physical violence and prevalent HIV infection among female sex workers

Figure 11. Pooled combined unadjusted and adjusted estimates of recent exposure to physical violence and prevalent HIV infection among female sex workers

Figure 12. Pooled combined unadjusted and adjusted estimates of lifetime exposure to physical violence and prevalent HIV infection among female sex workers

# Appendix S. Forest plots of sexual violence estimates and prevalent HIV infection

Figure 13. Pooled unadjusted estimates of recent exposure to sexual violence and prevalent HIV infection among female sex workers

Figure 14. Pooled unadjusted estimates of lifetime exposure to sexual violence and prevalent HIV infection among female sex workers

Figure 15. Pooled adjusted estimates of recent exposure to sexual violence and prevalent HIV infection among female sex workers

Figure 16. Pooled adjusted estimates of lifetime exposure to sexual violence and prevalent HIV infection among female sex workers

Figure 17. Pooled combined unadjusted and adjusted estimates of recent exposure to sexual violence and prevalent HIV infection among female sex workers

Figure 18. Pooled combined unadjusted and adjusted estimates of lifetime exposure to sexual violence and prevalent HIV infection among female sex workers

# Appendix T. Forest plots of mixed violence estimates and prevalent HIV infection

Figure 19. Pooled unadjusted estimates of recent exposure to mixed violence and prevalent HIV infection among female sex workers

Figure 20. Pooled unadjusted estimates of lifetime exposure to mixed violence and prevalent HIV infection among female sex workers

Figure 21. Pooled adjusted estimates of recent exposure to mixed violence and prevalent HIV infection among female sex workers

Figure 22. Pooled adjusted estimates of lifetime exposure to mixed violence and prevalent HIV infection among female sex workers

Figure 23. Pooled combined unadjusted and adjusted estimates of recent exposure to mixed violence and prevalent HIV infection among female sex workers

Figure 24. Pooled combined unadjusted and adjusted estimates of lifetime exposure to mixed violence and prevalent HIV infection among female sex workers

# Appendix U. Forest plots of other forms of violence estimates and prevalent HIV infection

Figure 25. Pooled unadjusted estimates of recent exposure to other forms of violence and prevalent HIV infection among female sex workers

Figure 26. Pooled adjusted estimates of recent exposure to other forms of violence and prevalent HIV infection among female sex workers

Figure 27. Pooled combined unadjusted and adjusted estimates of recent exposure to other forms of violence and prevalent HIV infection among female sex workers

# Appendix V. Forest plots of violence and HIV testing

Figure 28. Pooled unadjusted estimates of recent exposure to all forms of violence and HIV testing among female sex workers

Figure 29. Pooled unadjusted estimates of lifetime exposure to all forms of violence and HIV testing among female sex workers

Figure 30. Pooled adjusted estimates of recent exposure to all forms of violence and HIV testing among female sex workers

Figure 31. Pooled adjusted estimates of lifetime exposure to all forms of violence and HIV testing among female sex workers

Figure 32. Pooled combined unadjusted and adjusted estimates of recent exposure to all forms of violence and HIV testing among female sex workers

Figure 33. Pooled combined unadjusted and adjusted estimates of lifetime exposure to all forms of violence and HIV testing among female sex workers

# Appendix W. Forest plots of violence and ART outcomes

Figure 34. Pooled unadjusted estimates of recent exposure to all forms of violence and ART use among female sex workers

Figure 35. Pooled unadjusted estimates of lifetime exposure to all forms of violence and ART use among female sex workers

Figure 36. Pooled adjusted estimates of recent exposure to all forms of violence and ART use among female sex workers

Figure 37. Pooled adjusted estimates of lifetime exposure to all forms of violence and ART use among female sex workers

Figure 38. Pooled combined unadjusted and adjusted estimates of recent exposure to all forms of violence and ART use among female sex workers

Figure 39. Pooled combined unadjusted and adjusted estimates of lifetime exposure to all forms of violence and ART use among female sex workers

Figure 40. Pooled unadjusted estimates of recent exposure to all forms of violence and ART adherence among female sex workers

Figure 41. Pooled adjusted estimates of recent exposure to all forms of violence and ART adherence among female sex workers

Figure 42. Pooled combined unadjusted and adjusted estimates of recent exposure to all forms of violence and ART adherence among female sex workers

# Appendix X. Forest plots of violence and HIV viral suppression

Figure 43. Pooled unadjusted estimates of recent exposure to all forms of violence and HIV viral suppression among female sex workers

Figure 44. Pooled unadjusted estimates of lifetime exposure to all forms of violence and HIV viral suppression among female sex workers

Figure 45. Pooled adjusted estimates of lifetime exposure to all forms of violence and HIV viral suppression among female sex workers

Figure 46. Pooled combined unadjusted and adjusted estimates of recent exposure to all forms of violence and HIV viral suppression among female sex workers

Figure 47. Pooled combined unadjusted and adjusted estimates of lifetime exposure to all forms of violence and HIV viral suppression among female sex workers

# Appendix Y. Subgroup analyses

Figure 48. Subgroup analyses of recent exposure to all forms of violence and HIV infection among female sex workers

Figure 49. Subgroup analyses of lifetime exposure to all forms of violence and HIV infection among female sex workers

# Appendix Z. Publication asymmetry

For prevalent HIV infection, we observed asymmetry in the funnel plot of the associations with recent experiences of any type of violence, which was confirmed by the Egger’s test (P=0.001). When stratified by violence type, this asymmetry was most evident for recent sexual (P<0.001) or physical violence (P=0.015). There was no evidence of asymmetry for associations between lifetime experiences of violence and prevalent HIV infection (P=0.260). We did not detect asymmetry for estimates of the associations between lifetime violence and HIV testing (P=0.780), and there were fewer than 10 estimates for recent violence. We found evidence of asymmetry for associations between recent violence and ART use from funnel plots and Egger’s tests (P=0.013). There were fewer than 10 estimates for recent and lifetime violence and HIV incidence, ART adherence and HIV viral suppression, so we could not reliably assess for asymmetry.

Figure 50. Funnel plots for recent and lifetime exposure to all forms of violence and prevalent HIV infection

Figure 51. Funnel plots for recent and lifetime experiences of physical violence and prevalent HIV infection

Figure 52. Funnel plots for recent and lifetime experiences of sexual violence and prevalent HIV infection

Figure 53. Funnel plots for recent and lifetime experiences of physical and/or sexual violence and prevalent HIV infection

Figure 54. Funnel plots for recent and lifetime experiences of all forms of violence and HIV testing

Figure 55. Funnel plots for recent and lifetime experiences of all forms of violence and ART use

Figure 56. Funnel plots for recent experiences of all forms of violence and ART adherence

Figure 57. Funnel plots for recent and lifetime experiences of all forms of violence and HIV viral suppression

Figure 58. Recent and lifetime exposure to all forms of violence and prevalent HIV infection (Trim and Fill plots)

Figure 59. Recent and lifetime exposure to physical violence and prevalent HIV infection (Trim and Fill plots)

Figure 60. Recent and lifetime exposure to sexual violence and prevalent HIV infection (Trim and Fill plots)

Figure 61. Recent and lifetime exposure to mixed violence and prevalent HIV infection (Trim and Fill plots)

Table 15. Trim and fill analyses of experiences of violence and prevalent HIV infection

| **Violence Type** | **Exposure** | **Original pooled effect (95% CI)** | **Trim & Fill pooled effect (95% CI)** |
| --- | --- | --- | --- |
| All violence | Recent | 1.34 (1.14-1.58) | 1.23 (1.02-1.49) |
| Physical violence | Recent | 1.19 (0.99-1.42) | 1.12 (0.92-1.37) |
| Sexual violence | Recent | 1.55 (1.24-1.94) | 1.36 (1.03-1.79) |
| Mixed violence | Recent | 1.26 (1.06-1.50) | 1.29 (1.08-1.54) |
| All violence | Ever | 1.31 (1.19-1.44) | 1.25 (1.13-1.38) |
| Physical violence | Ever | 1.29 (1.15-1.44) | 1.28 (1.15-1.42) |
| Sexual violence | Ever | 1.40 (1.21-1.63) | 1.33 (1.17-1.51) |
| Mixed violence | Ever | 1.29 (1.13-1.48) | 1.37 (1.20-1.56) |

# Appendix AA. Sensitivity analyses of RHO

Table 16. Sensitivity analyses of all forms of violence and HIV outcomes for RHO=0.4, RHO=0.6 and RHO=0.8

| **Outcome** | **Timeframe** | **RHO = 0.4, OR (95% CI)** | **RHO = 0.6, OR (95% CI)** | **RHO = 0.8, OR (95% CI)** |
| --- | --- | --- | --- | --- |
| Prevalent HIV infection | Recent | 1.34 (1.17–1.52) | 1.33 (1.17–1.51) | 1.32 (1.16–1.49) |
| Prevalent HIV infection | Lifetime | 1.35 (1.24–1.47) | 1.36 (1.24–1.49) | 1.38 (1.22–1.55) |
| HIV testing | Recent | 1.12 (0.86–1.46) | 1.11 (0.86–1.45) | 1.11 (0.86–1.43) |
| HIV testing | Lifetime | 1.07 (0.83–1.37) | 1.06 (0.83–1.37) | 1.06 (0.82–1.37) |
| ART use | Recent | 0.76 (0.63–0.93) | 0.78 (0.64–0.94) | 0.74 (0.57–0.97) |
| ART use | Lifetime | 0.71 (0.35–1.42) | 0.71 (0.35–1.45) | 0.72 (0.35–1.47) |
| ART adherence | Recent | 0.65 (0.33–1.30) | 0.65 (0.33–1.29) | 0.65 (0.32–1.29) |
| HIV viral suppression | Recent | 0.89 (0.67–1.17) | 0.88 (0.67–1.17) | 0.90 (0.63–1.29) |
| HIV viral suppression | Lifetime | 0.88 (0.80–0.97) | 0.88 (0.79–0.98) | 0.87 (0.77–0.99) |

# Appendix BB. Sensitivity analysis of studies where 100% were FSWs

Figure 62. Pooled combined unadjusted and adjusted estimates of recent exposure to mixed violence and prevalent HIV infection among female sex workers excluding study with less than 100% FSWs

# Reference list

1.	Abdella S, Demissie M, Worku A, Dheresa M, Berhane Y. Ao - Abdella S, <https://orcid.org> O. HIV prevalence and associated factors among female sex workers in Ethiopia, east Africa: A cross-sectional study using a respondent-driven sampling technique. EClinicalMedicine. 2022;51:101540.

2.	Aho J, Koushik A, Coutlee F, Diakite SL, Rashed S. Prevalence of HIV, human papillomavirus type 16 and herpes simplex virus type 2 among female sex workers in Guinea and associated factors. International journal of STD & AIDS. 2014;25(4):280-8.

3.	Alaei A, Bromberg DJ, Tate MM, Karimov S, Saidi D, Alaei K. Ao - Alaei A, et al. HIV and associated factors among female sex workers in Tajikistan: Analysis from a national bio-behavioral survey. International Journal of STD and AIDS. 2021;32(8):678-86.

4.	Alary M, Banandur P, Rajaram SP, Thamattoor UK, Mainkar MK, Paranjape R, et al. Increased HIV prevention program coverage and decline in HIV prevalence among female sex workers in south India. Sex Transm Dis. 2014;41(6):380-7.

5.	Alemu GH, Gidebo DD, Ali MM. Magnitude of HIV Infection and Associated Factors among Female Sex Workers at Hawassa, Ethiopia. Ethiop j health sci. 2022;32(2):261-8.

6.	Amogne MD, Balcha TT, Agardh A. Prevalence and correlates of physical violence and rape among female sex workers in Ethiopia: a cross-sectional study with respondent-driven sampling from 11 major towns. BMJ open. 2019;9(7):e028247.

7.	Arimide DA, Amogne MD, Kebede Y, Balcha TT, Adugna F, Ramos A, et al. High Level of HIV Drug Resistance and Virologic Nonsuppression Among Female Sex Workers in Ethiopia: A Nationwide Cross-Sectional Study. Journal of acquired immune deficiency syndromes (1999). 2022;89(5):566-74.

8.	Arumugam E, Aridoss S, David JK, Jaganathasamy N, Balasubramanian G, Natesan M, et al. Injecting drug use & HIV prevalence among female sex workers: Evidence from the National Integrated Biological & Behavioural Surveillance, India. Indian J Med Res. 2022;155(3):413-22.

9.	Ayamah P, Aheto JMK, Atuahene KS, Annang DA, Nartey DT, Amuasi SA, et al. Multiple indicators of violence against female sex workers and its associated factors in Ghana: Evidence from the 2015 integrated bio-behavioral surveillance survey cross-sectional study. Health Science Reports. 2023;6(5):e1243.

10.	Beattie TSH, Bhattacharjee P, Ramesh BM, Gurnani V, Anthony J, Isac S, et al. Violence against female sex workers in Karnataka state, south India: impact on health, and reductions in violence following an intervention program. BMC public health. 2010;10(100968562):476.

11.	Beattie TS, Bhattacharjee P, Isac S, Mohan HL, Simic-Lawson M, Ramesh BM, et al. Declines in violence and police arrest among female sex workers in Karnataka state, south India, following a comprehensive HIV prevention programme. Journal of the International AIDS Society. 2015;18(1):20079.

12.	Beksinska A, Prakash R, Isac S, Mohan HL, Platt L, Blanchard J, et al. Violence experience by perpetrator and associations with HIV/STI risk and infection: a cross-sectional study among female sex workers in Karnataka, south India. BMJ open. 2018;8(9):e021389.

13.	Berger BO, Grosso A, Adams D, Ketende S, Sithole B, Mabuza XS, et al. The Prevalence and Correlates of Physical and Sexual Violence Affecting Female Sex Workers in Swaziland. Journal of interpersonal violence. 2018;33(17):2745-66.

14.	Bhardwaj A, Comins CA, Guddera V, McIngana M, Young K, Phetlhu R, et al. Prevalence of depression, syndemic factors and their impact on viral suppression among female sex workers living with HIV in eThekwini, South Africa. BMC Women's Health. 2023;23(1):232.

15.	Bowring AL, Ketende S, Billong SC, Mfochive Njindam I, Rao A, Decker MR, et al. Characterizing Sociostructural Associations with New HIV Diagnoses among Female Sex Workers in Cameroon. Journal of Acquired Immune Deficiency Syndromes. 2019;80(3):E64-E73.

16.	Braunstein SL, Ingabire CM, Geubbels E, Vyankandondera J, Umulisa MM, Gahiro E, et al. High burden of prevalent and recently acquired HIV among female sex workers and female HIV voluntary testing center clients in Kigali, Rwanda. PLoS ONE. 2011;6(9):e24321.

17.	Budhwani H, Newton S, Hearld KR, Levermore K. Condom use and HIV status: A study of sex workers engaged with the Jamaican AIDS support for life (JASL). West Indian Medical Journal. 2017;66(1):132-6.

18.	Budhwani H, Hearld KR, Butame SA, Naar S, Tapia L, Paulino-Ramírez R. Transgender women in Dominican Republic: HIV, stigma, substances, and sex work. AIDS Patient Care and STDs. 2021;35(12):488-94.

19.	Bugssa G, Dessalegn B, Dimtsu B, Berhane Y. Prevalence and factors associated with HIV and hepatitis B virus infections among female commercial sex workers in mekelle, Ethiopia: Cross sectional study. Int J Pharm Sci Res. 2015;6(1):135-46.

20.	Chabata ST, Hensen B, Chiyaka T, Mushati P, Mtetwa S, Hanisch D, et al. Changes Over Time in HIV Prevalence and Sexual Behaviour Among Young Female Sex-Workers in 14 Sites in Zimbabwe, 2013-2016. AIDS and behavior. 2019;23(6):1494-507.

21.	Chirombo J, Lora WS, Sakala D, Shahmanesh M, Ali S, Chabata ST, et al. An exploratory study using respondent-driven sampling to map HIV risk across sex work locations in Blantyre, Malawi. JAIDS Journal of Acquired Immune Deficiency Syndromes. 2022:10.1097.

22.	Coetzee J, Jewkes R, Gray GE. Cross-sectional study of female sex workers in Soweto, South Africa: Factors associated with HIV infection. PLoS ONE. 2017;12(10):e0184775.

23.	Comins CA, Baral S, Mcingana M, Shipp L, Phetlhu DR, Young K, et al. ART coverage and viral suppression among female sex workers living with HIV in eThekwini, South Africa: Baseline findings from the Siyaphambili study. PLOS Global Public Health. 2024;4(5):e0002783.

24.	Damacena GN, Szwarcwald CL, De Souza Junior PRB, Dourado I. Risk factors associated with HIV prevalence among female sex workers in 10 Brazilian Cities. Journal of Acquired Immune Deficiency Syndromes. 2011;57:S144-S52.

25.	Davey C, Dirawo J, Hargreaves JR, Cowan FM. Exploring the Association Between Mobility and Access to HIV Services Among Female Sex Workers in Zimbabwe. AIDS and behavior. 2020;24(3):746-61.

26.	Decker MR, Wirtz AL, Baral SD, Peryshkina A, Mogilnyi V, Weber RA, et al. Injection drug use, sexual risk, violence and STI/HIV among Moscow female sex workers. Sexually Transmitted Infections. 2012;88(4):278-83.

27.	Decker MR, Wirtz AL, Moguilnyi V, Peryshkina A, Ostrovskaya M, Nikita M, et al. Female sex workers in three cities in Russia: HIV prevalence, risk factors and experience with targeted HIV prevention. AIDS and behavior. 2014;18(3):562-72.

28.	Decker MR, Lyons C, Billong SC, Njindam IM, Grosso A, Nunez GT, et al. Gender-based violence against female sex workers in Cameroon: Prevalence and associations with sexual HIV risk and access to health services and justice. Sexually Transmitted Infections. 2016;92(8):599-604.

29.	Doshi RH, Sande E, Ogwal M, Kiyingi H, McIntyre A, Kusiima J, et al. Progress toward UNAIDS 90-90-90 targets: A respondent-driven survey among female sex workers in Kampala, Uganda. PloS one. 2018;13(9):e0201352.

30.	Duff P, Goldenberg S, Deering K, Montaner J, Nguyen P, Dobrer S, et al. Barriers to Viral Suppression among Female Sex Workers: Role of Structural and Intimate Partner Dynamics. Journal of Acquired Immune Deficiency Syndromes. 2016;73(1):83-90.

31.	Emmanuel F, Achakzai BK, Reza T. Prevalence and factors associated with HIV epidemic among female sex workers in Pakistan: Results of the fifth round of integrated biological and behavioural surveillance. Sexually Transmitted Infections. 2021;97(6):446-51.

32.	Faini D, Msafiri F, Munseri P, Bakari M, Lyamuya E, Sandstrom E, et al. The Prevalence, Incidence, and Risk Factors for HIV Among Female Sex Workers-A Cohort Being Prepared for a Phase IIb HIV Vaccine Trial in Dar es Salaam, Tanzania. Journal of Acquired Immune Deficiency Syndromes. 2022;91(5):439-48.

33.	Goldenberg SM, Rangel G, Staines H, Vera A, Lozada R, Nguyen L, et al. Individual, interpersonal, and social-structural correlates of involuntary sex exchange among female sex workers in Two Mexico–US Border Cities. JAIDS Journal of Acquired Immune Deficiency Syndromes. 2013;63(5):639-46.

34.	Goldenberg SM, Muzaaya G, Akello M, Nguyen P, Birungi J, Shannon K. War-Related Abduction and History of Incarceration Linked to High Burden of HIV among Female Sex Workers in Conflict-Affected Northern Uganda. Journal of Acquired Immune Deficiency Syndromes. 2016;73(1):109-16.

35.	Goldenberg SM, Montaner J, Duff P, Nguyen P, Dobrer S, Guillemi S, et al. Structural Barriers to Antiretroviral Therapy Among Sex Workers Living with HIV: Findings of a Longitudinal Study in Vancouver, Canada. AIDS and behavior. 2016;20(5):977-86.

36.	Goldenberg SM, Muzaaya G, Akello M, Braschel M, Birungi J, Shannon K. High burden of previously undiagnosed HIV infections and gaps in HIV care cascade for conflict-affected female sex workers in northern Uganda. International journal of STD & AIDS. 2019;30(3):275-83.

37.	Guure C, Dery S, Afagbedzi S, Maya E, da-Costa Vroom FB, Torpey K. Correlates of prevalent HIV infection among adolescents, young adults, and older adult female sex workers in Ghana: Analysis of data from the Ghana biobehavioral survey. PloS one. 2023;18(11):e0292686.

38.	Hendrickson ZM, Leddy AM, Galai N, Mbwambo JK, Likindikoki S, Kerrigan DL. Work-related mobility and experiences of gender-based violence among female sex workers in Iringa, Tanzania: a cross-sectional analysis of baseline data from Project Shikamana. BMJ open. 2018;8(9):e022621.

39.	Hensen B, Chabata ST, Floyd S, Chiyaka T, Mushati P, Busza J, et al. HIV risk among young women who sell sex by whether they identify as sex workers: analysis of respondent-driven sampling surveys, Zimbabwe, 2017. Journal of the International AIDS Society. 2019;22(12):e25410.

40.	Hladik W, Baughman AL, Serwadda D, Tappero JW, Kwezi R, Nakato ND, et al. Burden and characteristics of HIV infection among female sex workers in Kampala, Uganda - a respondent-driven sampling survey. BMC public health. 2017;17(1):565.

41.	Iakunchykova OP, Burlaka V. Correlates of HIV and Inconsistent Condom Use Among Female Sex Workers in Ukraine. AIDS and behavior. 2017;21(8):2306-15.

42.	Izadi N, Gouya MM, Akbarpour S, Zareie B, Moradi Y, Kazerooni PA, et al. HIV prevalence and associated factors among female sex workers in Iran: a bio-behavioral survey in 2020. AIDS and behavior. 2023;27(3):909-18.

43.	Jennings Mayo-Wilson L, Yen BJ, Nabunya P, Bahar OS, Wright BN, Kiyingi J, et al. Economic Abuse and Care-seeking Practices for HIV and Financial Support Services in Women Employed by Sex Work: A Cross-Sectional Baseline Assessment of a Clinical Trial Cohort in Uganda. Journal of interpersonal violence. 2023;38(1):NP1920-NP49.

44.	Jones HS, Hensen B, Musemburi S, Chinyanganya L, Takaruza A, Chabata ST, et al. Temporal trends in, and risk factors for, HIV seroconversion among female sex workers accessing Zimbabwe's national sex worker programme, 2009-19: a retrospective cohort analysis of routinely collected HIV testing data. The lancet HIV. 2023;10(7):e442-e52.

45.	Johnston L, Phatak G, El Rhilani H, El Kettani A, Khoudri I, McLaughlin KR. HIV and Syphilis Prevalence, Trends and Factors Affecting HIV Testing Among Female Sex Workers in Morocco. AIDS and Behavior. 2025;29(7):2158-67.

46.	Kakisingi C, Muteba M, Mukuku O, Kyabu V, Ngwej K, Kajimb P, et al. Prevalence and characteristics of HIV infection among female sex workers in Lubumbashi, Democratic Republic of Congo. Pan Afr Med J. 2020;36(101517926):280.

47.	Kassanjee R, Welte A, Otwombe K, Jaffer M, Milovanovic M, Hlongwane K, et al. HIV incidence estimation among female sex workers in South Africa: a multiple methods analysis of cross-sectional survey data. The Lancet HIV. 2022;9(11):e781-e90.

48.	Kelly-Hanku A, Weikum D, Badman SG, Willie B, Boli-Neo R, Kupul M, et al. Factors associated with HIV and syphilis infection among female sex workers in three cities in Papua New Guinea: Findings from Kauntim mi tu, a biobehavioral survey. Sexual Health. 2020;17(4):311-20.

49.	Kerrigan D, Mbwambo J, Likindikoki S, Beckham S, Mwampashi A, Shembilu C, et al. Project Shikamana: Baseline findings from a community empowerment-based combination HIV prevention trial among female sex workers in iringa, tanzania. Journal of Acquired Immune Deficiency Syndromes. 2017;74:S60-S8.

50.	Kumar GA, Dandona R, Alary M, Dandona L. Predictors of HIV prevalence among street-based female sex workers in Andhra Pradesh state of India: A district-level analysis. BMC Women's Health. 2014;14(1):65.

51.	Leddy AM, Underwood C, Decker MR, Mbwambo J, Likindikoki S, Galai N, et al. Adapting the Risk Environment Framework to Understand Substance Use, Gender-Based Violence, and HIV Risk Behaviors Among Female Sex Workers in Tanzania. AIDS and behavior. 2018;22(10):3296-306.

52.	Lima FSdS, Merchán-Hamann E, Urdaneta M, Damacena GN, Szwarcwald CL. Factors associated with violence against female sex workers in ten Brazilian cities. Cadernos de saude publica. 2017;33:e00157815.

53.	Luiz N, Muleia R, Abecasis A, Banze A, Langa D, Semá Baltazar C. Unveiling triple vulnerability among Mozambican female sex workers—Stigma, physical violence and sexual violence. Plos one. 2025;20(2):e0312550.

54.	Lyons CE, Grosso A, Drame FM, Ketende S, Diouf D, Ba I, et al. Physical and sexual violence affecting female sex workers in Abidjan, cote d'ivoire: Prevalence, and the relationship with the work environment, HIV, and access to health services. Journal of Acquired Immune Deficiency Syndromes. 2017;75(1):9-17.

55.	Lyons CE, Schwartz SR, Murray SM, Shannon K, Diouf D, Mothopeng T, et al. The role of sex work laws and stigmas in increasing HIV risks among sex workers. Nature Communications. 2020;11(1):773.

56.	MacLin BJ, Wang Y, Rodriguez-Diaz C, Donastorg Y, Perez M, Gomez H, et al. Comparing typologies of violence exposure and associations with syndemic health outcomes among cisgender and transgender female sex workers living with HIV in the Dominican Republic. PLoS ONE. 2023;18(9):e0291314.

57.	Mendoza C, Barrington C, Donastorg Y, Perez M, Fleming PJ, Decker MR, et al. Violence from a sexual partner is significantly associated with poor HIV care and treatment outcomes among female sex workers in the Dominican Republic. Journal of Acquired Immune Deficiency Syndromes. 2017;74(3):273-8.

58.	Mizinduko MM, Moen K, Likindikoki S, Mwijage A, Leyna GH, Makyao N, et al. HIV prevalence and associated risk factors among female sex workers in Dar es Salaam, Tanzania: tracking the epidemic. International Journal of STD and AIDS. 2020;31(10):950-7.

59.	Mountain E, Pickles M, Bhattacharjee P, Musyoki H, Mishra S, Cheuk E, et al. Experience of violence is associated with HIV prevention uptake and HIV infection among young women who self-identify as sex workers in Mombasa, Kenya. AIDS Research and Human Retroviruses. 2018;34:234.

60.	Mukherjee TI, Pala AN, Terlikbayeva A, Davis A, Howard AA, McCrimmon T, et al. Social and structural determinants of health associated with police violence victimization: A latent class analysis of female sex workers who use drugs in Kazakhstan. International Journal of Drug Policy. 2022;106:103750.

61.	Mukuku O, Kiakuvue YN, Numbi GY, Ruhindiza BM, Kakisingi C, Mwamba CM, et al. Assessing high-risk sexual practices associated with human immunodeficiency virus infection among young female sex workers in Lubumbashi, Democratic Republic of the Congo: a cross-sectional study. AIDS research and therapy. 2024;21(1):16.

62.	Mulholland GE, Markiewicz M, Arimi P, Ssengooba F, Weir S, Edwards JK. HIV prevalence and the HIV treatment Cascade among female sex Workers in Cross-Border Areas in East Africa. AIDS and Behavior. 2022;26(2):556-68.

63.	Mutagoma M, Samuel MS, Kayitesi C, Gasasira AR, Chitou B, Boer K, et al. High HIV prevalence and associated risk factors among female sex workers in Rwanda. International Journal of STD and AIDS. 2017;28(11):1082-9.

64.	Mutagoma M, Nyirazinyoye L, Sebuhoro D, Riedel DJ, Ntaganira J. Sexual and physical violence and associated factors among female sex workers in Rwanda: a cross-sectional survey. International journal of STD & AIDS. 2019;30(3):241-8.

65.	Nabayinda J, Namirembe R, Kizito S, Nsubuga E, Nabunya P, Sensoy Bahar O, et al. Correlates of Intimate Partner Violence Among Young Women Engaged in Sex Work in Southern Uganda. Journal of interpersonal violence. 2023;38(19):10749-70.

66.	Namale G, Kamacooko O, Bagiire D, Mayanja Y, Abaasa A, Kilembe W, et al. Sustained virological response and drug resistance among female sex workers living with HIV on antiretroviral therapy in Kampala, Uganda: A cross-sectional study. Sexually Transmitted Infections. 2019;95(6):405-11.

67.	Oldenburg CE, Ortblad KF, Chanda MM, Mwale M, Chongo S, Kanchele C, et al. Intimate partner violence and antiretroviral therapy initiation among female sex workers newly diagnosed with HIV in Zambia: A prospective study. Journal of Acquired Immune Deficiency Syndromes. 2018;79(4):435-9.

68.	Ouma S, Ndejjo R, Abbo C, Tumwesigye NM. Client-perpetrated gender-based violence among female sex workers in conflict-affected Northern Uganda: A cross-sectional study. BMJ Open. 2021;11(9):e046894.

69.	Pando MA, Coloccini RS, Reynaga E, Rodriguez Fermepin M, Gallo Vaulet L, Kochel TJ, et al. Violence as a Barrier for HIV Prevention among Female Sex Workers in Argentina. PLoS ONE. 2013;8(1):e54147.

70.	Prangnell A, Shannon K, Nosova E, DeBeck K, Milloy M, Kerr T, et al. Workplace violence among female sex workers who use drugs in Vancouver, Canada: Does client targeted policing increase safety? Journal of public health policy. 2018;39(1):86.

71.	Ramesh S, Ganju D, Mahapatra B, Mishra RM, Saggurti N. Relationship between mobility, violence and HIV/STI among female sex workers in Andhra Pradesh, India. BMC public health. 2012;12:764.

72.	Rice B, MacHingura F, Maringwa G, Magutshwa S, Kujeke T, Jamali G, et al. Brief Report: Adolescent Girls Who Sell Sex in Zimbabwe: HIV Risk, Behaviours, and Service Engagement. Journal of Acquired Immune Deficiency Syndromes. 2022;90(3):263-9.

73.	Schwartz S, Lambert A, Phaswana-Mafuya N, Kose Z, McIngana M, Holland C, et al. Engagement in the HIV care cascade and barriers to antiretroviral therapy uptake among female sex workers in Port Elizabeth, South Africa: Findings from a respondent-driven sampling study. Sexually Transmitted Infections. 2017;93(4):290-6.

74.	Sherman SG, Park JN, Galai N, Allen ST, Huettner SS, Silberzahn BE, et al. Drivers of HIV Infection among Cisgender and Transgender Female Sex Worker Populations in Baltimore City: Results from the SAPPHIRE Study. Journal of Acquired Immune Deficiency Syndromes. 2019;80(5):513-21.

75.	Shokoohi M, Noori A, Karamouzian M, Sharifi H, Khajehkazemi R, Fahimfar N, et al. Remaining gap in HIV testing uptake among female sex workers in Iran. AIDS and Behavior. 2017;21(8):2401-11.

76.	Shrestha R, Philip S, Shewade HD, Rawal B, Deuba K. Why don’t key populations access HIV testing and counselling centres in Nepal? Findings based on national surveillance survey. BMJ open. 2017;7(12):e017408.

77.	Sileo KM, Kintu M, Kiene SM. The intersection of intimate partner violence and HIV risk among women engaging in transactional sex in Ugandan fishing villages. AIDS Care - Psychological and Socio-Medical Aspects of AIDS/HIV. 2018;30(4):444-52.

78.	Strathdee SA, Lozada R, Martinez G, Vera A, Rusch M, Nguyen L, et al. Social and structural factors associated with HIV infection among female sex workers who inject drugs in the Mexico-US border region. PLoS ONE. 2011;6(4):e19048.

79.	Szwarcwald CL, Damacena GN, De Souza-Junior PRB, Guimaraes MDC, De Almeida WDS, De Souza Ferreira AP, et al. Factors associated with HIV infection among female sex workers in Brazil. Medicine (United States). 2018;97(1):S54-S61.

80.	Tokar A, Shulga L. Personal experience of violence as potential risk factor of HIV/STI Among FSWs in Ukraine. Sexually Transmitted Infections. 2013;89.

81.	Tounkara FK, Diabate S, Guedou FA, Ahoussinou C, Kintin F, Zannou DM, et al. Violence, condom breakage, and HIV infection among female sex workers in Benin, West Africa. Sex Transm Dis. 2014;41(5):312-8.

82.	Tuot S, Teo AKJ, Chhoun P, Mun P, Prem K, Yi S. Risk factors of HIV infection among female entertainment workers in Cambodia: Findings of a national survey. PloS one. 2020;15(12):e0244357.

83.	Ulibarri MD, Strathdee SA, Ulloa EC, Lozada R, Fraga MA, Magis-Rodriguez C, et al. Injection drug use as a mediator between client-perpetrated abuse and HIV status among female sex workers in two Mexico-US border cities. AIDS and behavior. 2011;15(1):179-85.

84.	Vandenhoudt HM, Langat L, Menten J, Odongo F, Oswago S, Luttah G, et al. Prevalence of HIV and other sexually transmitted infections among female sex workers in Kisumu, Western Kenya, 1997 and 2008. PloS one. 2013;8(1):e54953.

85.	Vu L, Misra K. High burden of HIV, syphilis and HSV-2 and factors associated with HIV infection among female sex Workers in Tanzania: implications for early treatment of HIV and pre-exposure prophylaxis (PrEP). AIDS and Behavior. 2018;22(4):1113-21.

86.	Wand H, Siba P. Prevalence and correlates of HIV infection among sex workers in Papua New Guinea: first results from the Papua New Guinea and Australia sexual health improvement project (PASHIP). AIDS and Behavior. 2015;19(12):2194-203.

87.	Wang M, Lim SH, Gibson BA, Azwa I, Guadamuz TE, Altice FL, et al. Correlates of newly diagnosed HIV infection among cisgender women sex workers and transgender women sex workers in Greater Kuala Lumpur, Malaysia. International journal of STD & AIDS. 2021;32(7):609-19.

88.	West BS, Henry BF, Agah N, Vera A, Beletsky L, Rangel MG, et al. Typologies and correlates of police violence against female sex workers who inject drugs at the México–United States border: Limits of de jure decriminalization in advancing health and human rights. Journal of interpersonal violence. 2022;37(11-12):NP8297-NP324.

89.	Wilson KS, Wanje G, Yuhas K, Simoni JM, Masese L, Vander Stoep A, et al. A prospective study of intimate partner violence as a risk factor for detectable plasma viral load in HIV-positive women engaged in transactional sex in Mombasa, Kenya. AIDS and behavior. 2016;20:2065-77.

90.	Wilson KS, Deya R, Masese L, Simoni JM, Stoep AV, Shafi J, et al. Prevalence and correlates of intimate partner violence in HIV-positive women engaged in transactional sex in Mombasa, Kenya. International journal of STD & AIDS. 2016;27(13):1194-203.

91.	Wirth KE, Tchetgen Tchetgen EJ, Silverman JG, Murray MB. How does sex trafficking increase the risk of HIV Infection? An observational study from Southern India. Am J Epidemiol. 2013;177(3):232-41.

92.	Xie Y, Xiong M, Qi Z, Shen J, Xu X, Luo X, et al. Client-perpetrated violence experience among female sex worker in Guangdong, South China: results from a cross-sectional study. AIDS and Behavior. 2023;27(3):806-15.

93.	Zalla LC, Herce ME, Edwards JK, Michel J, Weir SS. The burden of HIV among female sex workers, men who have sex with men and transgender women in Haiti: results from the 2016 Priorities for Local AIDS Control Efforts (PLACE) study. Journal of the International AIDS Society. 2019;22(7):e25281.

94.	Zhang C, Li X, Hong Y, Chen Y, Liu W, Zhou Y. Partner violence and HIV risk among female sex workers in China. AIDS and behavior. 2012;16(4):1020-30.
