# Supplementary material for: Impact of violence on HIV outcomes among female sex workers: A global systematic review and meta-analysis
Source: PLoS Med. 2026 Oct 21;23(10):e1004977. doi: 10.1371/journal.pmed.1004977 (PMC13712678; doi:10.1371/journal.pmed.1004977)
Supplement: Supplementary file 2 [file pmed.1004977.s004.docx]

***PLOS Medicine*:** “Impact of violence on HIV outcomes among female sex workers: A global systematic review and meta-analysis.”

Dear Editors,

We would like to thank the reviewers for their detailed and insightful comments on our manuscript. Our responses to each comment are outlined below, and we have made changes to the manuscript where appropriate. Line numbers refer to the tracked version of the document.

# Editor

1. Please note that we require the inclusion of an updated PRISMA 2025 checklist. Please also include sections rather than page numbers in the completed checklist.

Response: We used the 2020 PRISMA checklist as this is the current standard as per their website (<https://www.prisma-statement.org>). We have updated the PRISMA checklist to include sections rather than page numbers.

2. Editor: Please clarify the rationale for the time period of the search.

Response: We restricted our search to 2010 onwards to avoid including studies done in a period when the HIV epidemiology and care landscape was different. Prior to 2010, widespread ART access had not yet occurred in many LMICs, meaning studies from this period would reflect different treatment access and outcomes that are not comparable to the current context. To clarify this, we have added the following text to the methods:

“We included studies published after January 1^st^, 2010 to coincide with the widespread expansion and availability of ART in many LMICs.^1^” Lines 120–122, Methods

3. Editor: Please indicate how articles that were not in English were handled, as your Methods indicate that the search was not restricted by language.

Response: In previous systematic reviews, our group has used Google Translate to screen articles not published in English. However, we did not identify any eligible abstracts or full-text manuscripts that were not in English in this review.

4. Editor: Please provide more details in the Methods on the assessments of bias and study quality.

Response: Because of the nature of the studies eligible for our review, we felt that some of the domains of the Newcastle-Ottawa scale were not useful for distinguishing between the studies. For example, the Newcastle-Ottawa scale provides separate scoring systems for cross-sectional and cohort studies, but applied independently, these do not distinguish between designs: a cross-sectional and a cohort study of otherwise equal rigour would score the same, despite the stronger inference afforded by longitudinal data. We wanted to capture this difference using a dedicated score on study design and specifically, temporality between exposure and outcome. In addition, ascertaining violence exposure is challenging, and no included study used a secure record (e.g., surgical record). Therefore, all studies would be penalised for this domain. Similarly, our review included multiple outcomes that varied in how they could be ascertained. For HIV status and viral suppression, we excluded studies relying on self-report and included only those with confirmatory test results; as a result, all included studies would have received full marks on this domain, making it uninformative for distinguishing study quality. Conversely, for HIV testing and ART use, self-report is the norm in this literature, so requiring laboratory or clinical confirmation would have penalised nearly all included studies. Consequently, we felt that the representativeness, temporality of exposure and outcome, and adjustment for confounding were the most relevant domains for our review, as they directly address the major methodological variations between studies that influenced study quality.

We have updated the description of the study quality assessment with the following text:

“Study quality was assessed for each exposure-outcome estimate pair across three domains; study representativeness (with a score given for using representativeness recruitment approaches, including RDS, time-location or venue-based sampling, or recruitment through multiple, diverse sources), study design (longitudinal vs. cross-sectional) and whether estimates were adjusted or not (adjusted vs. unadjusted). These three domains were selected because they captured the primary sources of methodological variation across included studies. Other Newcastle-Ottawa scale domains, such as ascertainment of exposure, were not used because they would have penalised all studies equally, given that violence exposure cannot be verified against a secure record.” Lines 212–220, Methods

We would also like to draw attention to Appendix 7, in which we provide a detailed description of the study quality assessment.

5. Editor: Please include as a last sentence of the Methods and Findings section of the Abstract a statement on the limitations of the study methodology.

Response: We have added the following sentence to the abstract which lists the main limitations of our study:

“Our study had limitations, notably the cross-sectional design of most included studies, the heterogeneity in pooled estimates, a small number of studies for some outcomes, and the difficulties of measuring experiences of violence.” Lines 46–47, Abstract

6. Editor: Please note that we expect searches to be updated to within 6 months of the time of submission.

Response: We have updated the search to June 9^th,^ 2026.

7. Editor: Please include a point-by-point rebuttal to all reviewer and editor concerns upon resubmission.

Response: We have included this below.

# Reviewer One

The methods employ a variety very specific but not clearly justified choices. It is unclear what effect these have on the results.

**Comments:**

1. Reviewer: The arbitrary date of January 1st, 2010, or later, for inclusion of articles.

Response: We restricted our search to 2010 onwards to avoid including studies done in a period when the HIV epidemiology and care landscape was different. Prior to 2010, widespread ART access had not yet occurred in many LMICs, meaning studies from this period would reflect different treatment access and outcomes that are not comparable to the current context. To clarify this, we have added the following text to the methods:

“We included studies published after January 1^st^, 2010 to coincide with the widespread expansion and availability of ART in many LMICs.^1^” Lines 120–122, Methods

2. Reviewer: An unusual inclusion criterion: "could be used to estimate associations between any form of violence and one or more selected HIV outcomes among FSWs," which appears broad and difficult to verify.

Response: We apologise for the confusing phrasing of this criterion. To clarify, this inclusion criteria refers to studies in which enough information is available within the manuscript or its supplementary material to derive a measure of association, whether it was presented or not. For example, if there was a 2x2 contingency table with HIV prevalence (negative, positive) as columns, and sexual violence (no, yes) as rows, we could calculate an odds ratio and its corresponding 95% CI, even if one wasn’t presented. We have clarified this criteria with the following sentence:

“Eligible study designs included those that presented associations between any form of violence and one or more selected HIV outcomes among FSWs, or alternatively studies with sufficient information to calculate an association (i.e. a 2x2 contingency table), namely observational studies (and specifically, case-control studies, cohort studies, cross-sectional studies), and intervention studies including randomised controlled studies.” Lines 122–126, Methods

3. Reviewer: A rule that 70% of the sample had to include female sex workers.

Response: We used the 70% threshold so that we could include studies in which female sex workers were not the whole sample but still comprised most of the population under investigation, and so maintained a degree or representativeness. Notably, there was only one included study in which less than 100% of the sample were female sex workers (75%). We conducted a sensitivity analysis excluding this study, and found no change in our overall conclusions. This clarifying information has been added to the methods:

“So that we could include studies in which female sex workers were not the whole sample but still comprised most of the population under investigation, we included studies with a rule that greater than 70% of the included sample needed to have recently/ever engaged in sex work.” Lines 126–129, Methods

“We also conducted a sensitivity analysis in which we excluded studies where less than 100% of the sample were FSWs.” Lines 196–197, Methods

And the findings from the sensitivity analysis have been added to the results:

“Only one study had less than 100% of the sample being FSWs (75%), with this study reporting an association between mixed violence and prevalent HIV infection. We did not find any differences in our conclusions in a sensitivity analysis excluding this study (Appendix 29).” Lines 398–401, Results

4. Reviewer: A rule that studies had to have at least 40 participants.

Response: Our decision to only include studies with at least 40 participants was based on several publications which highlight that there are several concerns around (1) the reliability of estimated measures of association, (2) a higher possibility of selection bias in smaller studies more often rely on convenience samples (3) the risk that underpowered studies produce unstable or unreliable effect estimates, and (4) methodological quality.^2,3^ However, we recognise that this decision may have excluded studies for our ART and viral suppression outcomes, of which we included a smaller number of studies. We have therefore added the following text to the methods:

“Studies were excluded if they had a sample size below 40, to address concerns that there is a higher possibility of selection bias in smaller studies, and to prevent underpowered studies from skewing the results.^2,3^” Lines 143–145, Methods

And we have acknowledged this as a limitation of our review:

“Fifth, our decision to exclude studies with sample sizes less than 40 may have excluded studies that reported on associations with rarer outcomes, such as ART use and HIV viral suppression.” Lines 507–509, Results

5. Reviewer: The inclusion criteria about ability to "estimate associations" appears very broad and subjective. I wonder if there is a way that this could be articulated that is  more transparent and objective.

Response: Please refer to our answer to Comment 2.

6. Reviewer: The time frames for reports of violence and reports of set works do not necessarily align. It's possible that sex work may have occurred long before violence in the case of these.

Response: We thank the reviewer for this comment, and agree that there are challenges ascertaining the temporal order of violence exposures and sex work, particularly given the majority of included studies are cross-sectional. However, in our data extraction, we extracted the recall period used to define sex work, and note that only three of 94 included studies included a lifetime definition, two did not report a time frame, and the remaining 89 studies reported recent exposure (time frames ranging from current to past 12 months). Therefore, in most studies sex work is unlikely to have occurred long before the violence exposure. Even so, for measures of lifetime exposure to violence, we cannot rule out the possibility that the violence event had occurred before becoming a sex worker (unless this was explicitly defined as violence related to sex work). We have added additional subgroup analyses comparing recent and lifetime exposure to sex work, which resulted in no changes to our conclusions for both recent and lifetime exposures to violence.

To address this comment, the following text has been added to the results:

“Most studies (n=89; 95%) included FSWs who had recently engaged in sex work (time frames ranging from current to past 12 months), with two studies not reporting a time frame, and three studies including women with lifetime engagement in sex work.” Lines 248–251, Results

We have also added the subgroup analyses of recent vs. lifetime engagement in sex work to the supplements (Appendix 26).

7. Reviewer: The results say that three-quarters of the studies were considered representative. It's unclear how they judge the representativeness of the populations in the studies.

Response: We provide a full outline of the study quality domains in Appendix 7. As per Appendix 7, we write: “This domain assessed the extent to which the study sample was likely to approximate the target population. One point was assigned if the study used recruitment approaches intended to enhance representativeness among sex workers, including RDS, time-location or venue-based sampling, or recruitment through multiple, diverse sources (e.g., brothels or street outreach). Probability-based sampling, while rarely used, was also considered indicative of high representativeness. To receive a point, studies could not be restricted to narrowly defined subgroups (e.g., specific age groups, gender identities, or sexual orientations), unless such restrictions were intrinsic to the study population (e.g., all women in studies of FSW). Studies that did not meet these criteria were assigned zero points.”

We have added the following information to the main manuscript to briefly describe how we assessed representativeness:

“Study quality was assessed for each exposure-outcome estimate pair across three domains; study representativeness (with a score given for using representativeness recruitment approaches, including RDS, time-location or venue-based sampling, or recruitment through multiple, diverse sources), study design (longitudinal vs. cross-sectional) and whether estimates were adjusted or not (adjusted vs. unadjusted). These three domains were selected because they captured the primary sources of methodological variation across included studies. Other Newcastle-Ottawa scale domains, such as ascertainment of exposure, were not used because they would have penalised all studies equally, given that violence exposure cannot be verified against a secure record.” Lines 212–220, Methods

8. Reviewer: Overall, the methods appear to be solid and appropriate for the analysis. They used random effects. They assessed for heterogeneity as well as a publication bias. However, the results of these were not reassuring. There appears to be evidence of publication bias from the funnel plots, I am not sure if the funnel plots were given in the paper. In addition, in many cases, the heterogeneity is quite high.

Response: We thank the Reviewer for the positive feedback on the overall methodological approach. The funnel plots for our manuscript are provided in the supplementary material which we refer to in the main text and now comment on in more detail. We agree with the reviewer that the heterogeneity was high for some pooled effects, and that there was also evidence of funnel plot asymmetry for some pooled associations, warranting some caution in their interpretation. This has been previously addressed as a limitation as below, and we have now conducted an sensitivity analysis to account for funnel plot asymmetry by undertaking a trim and fill analysis for the pooled associations with HIV prevalence, which had most evidence of funnel plot asymmetry. Notably, the associations remained similar after the trim and fill analyses.

“Second, we found evidence of funnel plot asymmetry for the associations between recent experiences of violence and prevalent HIV infection and ART use, suggesting the possibility of publication bias. This asymmetry may be partially explained by our title and abstract screening strategy, as studies which did not detect associations between violence and HIV outcomes are less likely to report them in their abstract. However, funnel plot asymmetry may be explained by other factors, such as differences in study quality or design, heterogeneity in measurement methods, or statistical methods.^4^ Nonetheless, the results from the Egger’s tests suggest that our findings may be subject to publication bias.” Lines 484–491, Limitations

We describe this trim and fill sensitivity analysis in the methods:

“We also used trim-and-fill analyses to adjust for potential publication bias by imputing missing studies based on observed funnel plot asymmetry and recalculating the pooled effect estimates.^5^” Lines 200–202, Methods

We also updated the results with:

“In supplementary analyses where we used the trim-and-fill method to adjust for potential publication bias, we found minimal changes to the pooled effects (Appendix 27).” Lines 387–389, Results

As the Reviewer points out, we also observed substantial statistical heterogeneity across many meta-analyses. To better understand some of the underlying sources, we used subgroup analyses to assess heterogeneity for several factors, including WHO regions, perpetrators of violence, year of publication, recruitment method, representative of sample and whether studies adjusted for confounders, which are all reported in the supplementary material. As we report in the paper, we did not find any meaningful evidence for the source of heterogeneity, and the heterogeneity in the estimates is, at least in part, likely due to the measurement of violence as an exposure. Although we conducted subgroup analyses by several characteristics of the violence measure, we could stratify by only one dimension at a time (eg violence type, or recall period), whereas the underlying exposure varied simultaneously in type (sexual, physical, emotional), perpetrator (partner, client, police, or any), recall period, and the specific acts assessed. Studies within the same subgroup therefore often measured different exposures (e.g., recent sexual violence from a partner versus lifetime physical violence from any perpetrator), which we could not fully disentangle.

9. Reviewer: I feel that these issues should be discussed with more nuance in the discussion section. And investigated by reporting the effects by the subgroups or examining if these are explained by meta regression.

Response: Please refer to our answer to Comment 8.

10. Reviewer: That there's particular need for nuance around the association between prevalent HIV and violence, the cross-sectional nature of that association makes it difficult to infer the directionality of the association or the possibility that it's due to unmeasured confounding.

Response: We thank the reviewer for this comment and agree that the cross-sectional design of included studies means our findings may reflect a degree of reverse causality especially in the association between violence and HIV prevalence. Additionally, it may be that HIV prevalence is a marker of some other behaviour, such as street based sex work, that is related to experiencing more violence. We have therefore added the following sentences to the discussion:

“Further, our findings may also reflect the bi-directional relationship between HIV infection and experiences of violence, with disclosure of HIV status associated with substantially higher odds of intimate partner violence.^6,7^ Conversely, HIV may be a marker of other risk behaviours that are associated with heightened violence, such as street based sex work. For FSWs living with HIV, experiences of both sex work and HIV-related stigma further increases their vulnerability to violent experiences.^8^ Indeed, men who report sex with FSWs have been found to be more misogynistic, patriarchal and violent.^9^” Lines 436–443, Discussion

We have also added the following to the limitations:

“Ninth, many of the associations in our pooled analyses were unadjusted and subject to confounding, and some of the adjusted estimates may have unmeasured confounding, due to inadequate adjustment for relevant confounders.” Lines 529–531, Discussion

# Reviewer Two

This manuscript presents a comprehensive and timely systematic review and meta-analysis examining the association between violence and HIV outcomes among female sex workers (FSWs).  The use of multilevel random-effects models and robust variance estimation is methodologically appropriate given the dependence among effect sizes. Overall, the manuscript is well-written and addresses an important structural determinant of HIV risk. This is a strong and policy-relevant manuscript that advances understanding of structural determinants of HIV among FSWs. The authors may consider the following suggestions for improvement.

We thank the reviewer for their thoughtful and constructive comments.

Comments:

1. Reviewer: While the authors acknowledge that most included studies are cross-sectional, the interpretation of findings occasionally implies causal relationships (e.g., violence "increases" HIV risk). The language should be further tempered throughout the manuscript to emphasize associations rather than causal effects.

Response: We thank the reviewer for this comment and agree with the importance of tempering our language to align with the cross-sectional nature of included studies. For example, we have added the following text to describe how the association between violence and HIV may be bi-directional, rather than framing it as causal:

“Further, our findings may also reflect the bi-directional relationship between HIV infection and experiences of violence, with disclosure of HIV status associated with substantially higher odds of intimate partner violence.^6,7^ Conversely, HIV may be a marker of other risk behaviours that are associated with heightened violence, such as street based sex work. For FSWs living with HIV, experiences of both sex work and HIV-related stigma further increases their vulnerability to violent experiences.^8^ Indeed, men who report sex with FSWs have been found to be more misogynistic, patriarchal and violent.^9^” Lines 436–443, Discussion

2. Reviewer: There is substantial heterogeneity in how violence and HIV outcomes are defined and measured across studies. Although the authors grouped violence types, further discussion is needed on how this variability may bias pooled estimates and limit comparability, particularly for "mixed" and "other" violence categories.

Response: We agree with the reviewer that there was heterogeneity in the measurement of violence types between studies, particularly in the mixed and other categories. For this reason, we also presented findings separately when there are enough studies for a specific type of exposure, and did not find substantial differences in the pooled effects between physical, sexual, mixed and other forms of violence. We also discussed in the limitations section the issue of heterogeneities in the measures of violence exposures as follows:

“Third, there was heterogeneity in the measurement and definition of violence and some HIV outcomes across studies, which affected the comparability and synthesis of results. Whilst we conducted pooled analyses stratified by violence type, there were still variations in the definitions of violence within each strata. An important limitation of how violence is often measured in quantitative studies is the difficulty of measuring the nature and intensity of violent experiences, and so one person’s minor violence may be another person’s moderate violence. Consequently, our analysis risks homogenising minor and severe experiences of violence. Further, most studies did not account for other factors that may influence the relationship between violence and HIV outcomes, such as the perpetrator of violence, the type or frequency of violent experiences, and the timeframe over which violence occurred.” Lines 494–504, Limitations

3. Reviewer: The analysis of HIV incidence, ART adherence, and viral suppression is based on a small number of studies. The manuscript would benefit from a clearer emphasis on the limited statistical power and cautious interpretation of these findings, especially where null results are reported.

Response: We agree with the reviewer, and have added the following sentences to our limitations acknowledging the small number of studies looking at HIV incidence, ART adherence and viral suppression, and how the null findings may be related to this lower statistical power:

“Sixth, we only identified a small number of studies reporting on some outcomes, including ART use, ART adherence, HIV incidence, and HIV viral suppression, and some of these studies had relatively small sample sizes. Consequently, our pooled associations for these outcomes should be interpreted with caution, because we had lower statistical power.” Lines 512–516, Limitations

4. The authors note potential publication asymmetry. However, the search strategy (requiring violence to appear in titles/abstracts) may systematically exclude null findings. This should be more explicitly framed as a potential source of selection bias and discussed in terms of its impact on pooled estimates.

Response: In our original submission, we acknowledged this restriction to the title and abstract screening process as a limitation with the following text: “First, for inclusion in this review, our search terms required a study’s title and/or abstract or keywords and MESH terms to mention violence (or related terms) and our specific HIV outcomes among FSWs. Consequently, we would not have identified any study where the association between violence and one of our HIV outcomes was only included in the main text of the study.”. This limitation is common for systematic reviews, and as described in the Cochrane handbook for Systematic Reviews of Interventions, time restraints require the review team to balance the thoroughness of the search with efficiency in the use of time and funds.^10^

We also conducted a trim and fill analysis to address the issue of asymmetry in the funnel plots, describe in the methods:

“We also used trim-and-fill analyses to adjust for potential publication bias by imputing missing studies based on observed funnel plot asymmetry and recalculating the pooled effect estimates.^5^” Lines 200–202, Methods

We also updated the results with:

“In supplementary analyses where we used the trim-and-fill method to adjust for potential publication bias, we found minimal changes to the pooled effects (Appendix 27).” Lines 387–389, Results

5. Briefly explain the data extraction/review software (Rayyan AI) in the method section.

Response: We have provided the following text to the methods to describe Rayyan AI:

“Rayyan AI is a browser-based systematic review software, which allows users to highlight keywords for inclusion and exclusion, and streamlines the screening process by automatically flagging double-screened studies with conflicting decisions. Rayyan also has artificial intelligence capabilities which were not used for our review.” Lines 154–158, Methods

6. Why did authors exclude studies with sample sizes less than 40? How is the cut-off determined? More rationales should be provided.

Response: This comment aligns with another reviewer, and so we have added our response below:

Our decision to only include studies with at least 40 participants was based on several publications which highlight that there are several concerns around (1) the reliability of estimated measures of association, (2) a higher possibility of selection bias in smaller studies more often rely on convenience samples (3) the risk that underpowered studies produce unstable or unreliable effect estimates, and (4) methodological quality.^2,3^ However, we recognise that this decision may have excluded studies for our ART and viral suppression outcomes, of which we included a smaller number of studies. We have therefore added the following text to the methods:

“Studies were excluded if they had a sample size below 40, to address concerns that there is a higher possibility of selection bias in smaller studies, and to prevent underpowered studies from skewing the results.^2,3^” Lines 143–145, Methods

And we have acknowledged this as a limitation of our review:

“Fifth, our decision to exclude studies with sample sizes less than 40 may have excluded studies that reported on associations with rarer outcomes, such as ART use and HIV viral suppression.” Lines 510–512, Results

7. Clarify the definition and interpretation of "other violence," which appears highly heterogeneous and may obscure meaningful distinctions.

Response: When screened studies reported on a form of violence that did not constitute physical or sexual violence, we categorised these exposures as “other violence”. For example, if a study reported an association between “Psychological violence” and HIV prevalence, we categorised this exposure as “Other violence”. These forms of violence were included in our review because they reflect coercive and abusive behaviour, aligning with the aims of our review.

We describe the “other violence” exposure in our original submitted manuscript as: “Few studies reported on other forms of violence, and these measures were heterogeneous (e.g., emotional, psychological), therefore, they were grouped into a single “other violence” category.”. This classification was further described in Appendix 8, however we did not reference this appendix in our original submission, so this has now been amended. We have also updated our methods to provide more clarity and detail on how this exposure was classified:

“Few studies reported on other forms of violence, and these measures were heterogeneous (e.g., emotional abuse, economic abuse, threatened, psychological abuse), therefore, they were grouped into a single “other violence” category.” Lines 164–166, Methods

We have also added the following limitation:

“Seventh, our classification of “other forms of violence” included a number of heterogenous measures such as emotional abuse, economic abuse, threatened and psychological abuse, and therefore, the corresponding pooled estimate should be interpreted with caution.” Lines 517–519, Limitations

8. Provide more detail on quality assessment scoring and how it influenced interpretation (currently described briefly).

Response: Because of the nature of the studies eligible for our review, we felt that some of the domains of the Newcastle-Ottawa scale were not applicable. For example, ascertaining violence exposure is challenging, and no included study used a validated tool. Therefore, all studies would be penalised for this domain. Similarly, we excluded studies with self-reported HIV status and viral suppression, and only included those with confirmatory test results, thus all studies for these outcomes would receive full marks. For HIV testing and ART use, basing our assessment of the outcome on laboratory testing and clinical evidence would have penalised all included studies, given the challenges assessing these outcomes. Consequently, we felt that the representativeness, temporality of exposure and outcome, and adjustment for confounding were the most relevant domains for our review, as they directly address the major methodological variations between studies that influenced study quality.

We have updated the description of the quality assessment with the following text:

“Study quality was assessed for each exposure-outcome estimate pair across three domains; study representativeness (with a score given for using representativeness recruitment approaches, including RDS, time-location or venue-based sampling, or recruitment through multiple, diverse sources), study design (longitudinal vs. cross-sectional) and whether estimates were adjusted or not (adjusted vs. unadjusted). These three domains were selected because they captured the primary sources of methodological variation across included studies. Other Newcastle-Ottawa scale domains, such as ascertainment of exposure, were not used because they would have penalised all studies equally, given that violence exposure cannot be verified against a secure record.” Lines 212–220, Methods

We would also like to draw attention to Appendix 7, in which we provide a detailed description of the study quality assessment.

9. Consider adding a summary table of key covariates adjusted for in included studies.

Response: We have added this information to Appendices 12–15. We refer to these appendices, and the appendix of study characteristics in the results:

“Characteristics of studies and associations between violence and HIV outcomes are included in Appendices 11–15.” Lines 254–255, Results

10. Strengthen the policy/implementation implications by linking findings more explicitly to actionable intervention strategies.

Response: In response to this comment and a comment from another reviewer, we now have two paragraph on interventions for violence-reduction interventions:

“A key challenge for designing and implementing interventions that reduce violence among FSWs is the limited evidence base.^11^ However, there are interventions that have been demonstrated to be effective in reducing violence. For instance, community empowerment and peer group membership has been shown to reduce experiences of violence among FSWs^12-16^, increase their capacity to exercise autonomy and self-protection^13,15,16^, and potentially reduce HIV incidence among FSWs.^17,18^ Interventions focusing on social and structural contexts such as public health messaging, policy advocacy and education for law enforcement have also been shown to reduce experiences of violence among FSWs.^19-22^ This evidence suggests that structural interventions should combine advocacy, community mobilisation and police education to reduce violence among FSWs. Continued investment in comprehensive, community-driven interventions is therefore required to address the complex factors contributing to violence among FSWs, which should then improve their HIV outcomes and promote their health and human rights.

While these interventions can reduce violence experienced by FSWs, they largely mitigate risk within existing social conditions rather than addressing the structural systems that produce violence. That is, FSWs continue to work and live within patriarchal and misogynistic environments where violence is shaped by broader social, institutional, cultural, and political domains.^25^ Consequently, there is growing recognition that sustainable reductions in violence require interventions that promote systemic change alongside targeted programmes for FSWs.^26^ Evaluating these broader structural interventions is challenging, particularly in low-income settings,^27^ although evidence from violence-reduction interventions among women and girls demonstrates the potential of interventions which seek to transform gender norms and unequal power relations. These include community mobilisation initiatives, school- and community-based programmes that challenge gender norms and attitudes that condone violence^28-30^, and economic empowerment interventions, such as microfinance^31^ and vocational training.^32^ Multisectoral programmes that engage multiple stakeholders appear to be the most effective, as they simultaneously address gender inequality, women's economic dependence, and other structural drivers of violence.^27^ While such interventions have rarely been implemented specifically among FSWs, they highlight the importance of embedding FSW-focused interventions within wider efforts to transform the social and institutional conditions that sustain violence.” Lines 457–485, Discussion

# Reviewer Three

This manuscript represents a robust attempt to categorise and summarise the evidence for associations between violence and HIV outcomes in FSWs. In general, the authors should be applauded for their work and straightforward presentation of the results. Most comments are minor, and I believe that with some revisions, the manuscript should be suitable for publication. I thank the authors for their work and look forward to citing this helpful study in my own research.

We thank the reviewer for their kind comments.

**Abstract**

1. Reviewer: The study lays out a coherent rationale and approach. It also raises a few questions that I assume will be answered in the course of the paper. It would be helpful to know whether the 37 countries were all LMICs and whether analyses by type of violence (physical, sexual, etc.) were completed.

Response: We have added the following text about country-level income and violence types:

“We included 91 studies with 221 estimates, comprising 179,727 FSWs in 37 countries. Half of the included studies were in low- and middle-income countries (n=47, 50%), and one-fifth were in low-income countries (n=18; 19%). We found higher odds of prevalent HIV infection among FSWs with recent (pooled odds ratio (pOR):1.33; 95%CI:1.17–1.51; I^2^:64%; n=73 estimates) and lifetime (pOR:1.36; 95%CI:1.24–1.49; I^2^:38%; n=67) experiences of violence. Recently experiencing violence was associated with reduced odds of ART use (pOR:0.78; 95%CI:0.64–0.94; I^2^:8%; n=17). Lifetime exposure to violence was associated with reduced odds of viral suppression (pOR:0.88; 95%CI:0.79–0.98; I^2^:20%; n=6). Similar pooled effects were found when stratifying violence by type (physical violence, sexual violence, mixed violence, other violence). There was no evidence of associations between violence and HIV incidence, HIV testing and ART adherence.” Lines 33–45, Abstract

**Introduction**

2. Reviewer: LMICs stands for "low- and middle-income" not "lower- "

Response: This has been updated.

3. Reviewer: Please elaborate on the inequities that exist in the HIV cascade among FSWs.

Response: We have added the following text to the introduction summarising these inequities:

“Furthermore, evidence suggests that in many settings inequities exist in the HIV cascade of care among FSWs, including suboptimal antiretroviral therapy (ART) coverage and HIV viral suppression.^33-35^” Lines 80–82, Introduction

4. Reviewer: Overall, the Introduction suggest feels a bit stunted. The rationale for the study is well described, but if word limits allow, I elaborating throughout.

Response: We have expanded on the introduction with the following updated text:

“Studies have described how violence can contribute directly to HIV transmission, as well as influence HIV risk behaviours such as condomless sex and substance use.^36-38^ Modelling has also shown the potential impact of violence reduction interventions on averting HIV infections.^39^ Recent evidence suggests that violence is highly prevalent among FSWs, with estimates from a systematic review suggesting that one in four sex workers experience physical and/or sexual violence every year.^40^” Lines 88–93, Introduction

**Methods**

5. Reviewer: Why did the authors choose these three databases (and not more or others?)

Response: We selected PsycINFO, Embase and Medline as they have a comprehensive coverage of published journal articles on medicine and biomedical sciences, as we have done in previous systematic reviews.^41-44^ Whilst searching additional databases may have found some additional studies not indexed in these databases, these searches may have required us to screen a substantial number of irrelevant papers. In response to this comment, we have acknowledged this by adding the following limitation:

“Eighth, in this review, we searched PsycINFO, Embase, and MEDLINE, which provide broad coverage of biomedical and health-related literature. However, restricting the search to these three databases may have resulted in the omission of relevant studies indexed in other databases. However, expanding the search strategy would likely have substantially increased the volume of non-relevant records requiring screening. Nonetheless, some potentially relevant evidence may have been missed.” Lines 523–529, Limitations

6. Reviewer: Why did the authors choose to start their search in 2010? Some rationale for this choice is needed

Response: We restricted our search to 2010 onwards to avoid including studies done in a period when the HIV epidemiology and care landscape was different. Prior to 2010, widespread ART access had not yet occurred in many LMICs, meaning studies from this period would reflect different treatment access and outcomes that are not comparable to the current context. To clarify this, we have added the following text to the methods:

“We included studies published after January 1^st^, 2010 to coincide with the widespread expansion and availability of ART in many LMICs” Lines 120–122, Methods

7. Reviewer: A requirement that 70% of a sample needed to "recently/ever engaged in sex work" is a broad inclusion criterion- some rationale for this rule is needed

Response: This reviewer comment aligns with a previous comment, and so we have repeated our response below:

We used the 70% threshold so that we could include studies in which female sex workers were not the whole sample but still comprised most of the population under investigation, whilst still maintaining representativeness. Notably, there was only one included study in which less than 100% of the sample were female sex workers (75%). We therefore conducted a sensitivity analysis excluding this study, and found no change in our overall conclusions. This clarifying information has been added to the methods:

“So that we could include studies in which female sex workers were not the whole sample but still comprised most of the population under investigation, we included studies with a rule that greater than 70% of the included sample needed to have recently/ever engaged in sex work.” Lines 126–129, Methods

“We also conducted a sensitivity analysis in which we excluded studies where less than 100% of the sample were FSWs.” Lines 196–197, Methods

And the findings from the sensitivity analysis have been added to the results:

“Only one study had less than 100% of the sample being FSWs (75%), with this study reporting an association between mixed violence and prevalent HIV infection. We did not find any differences in our conclusions in a sensitivity analysis excluding this study (Appendix 29).” Lines 398–401, Results

8. Reviewer: Similarly, why N=40 for the sample size rule? These are all defensible, but the authors need to provide such a defense

Response: This comment aligns with another reviewer, and so we have added our response below:

Our decision to only include studies with at least 40 participants was based on several publications which highlight that there are several concerns around (1) the reliability of estimated measures of association, (2) a higher possibility of selection bias in smaller studies more often rely on convenience samples (3) the risk that underpowered studies produce unstable or unreliable effect estimates, and (4) methodological quality.^2,3^ However, we recognise that this decision may have excluded studies for our ART and viral suppression outcomes, of which we included a smaller number of studies. We have therefore added the following text to the methods:

“Studies were excluded if they had a sample size below 40, to address concerns that there is a higher possibility of selection bias in smaller studies, and to prevent underpowered studies from skewing the results.^2,3^” Lines 143–145, Methods

And we have acknowledged this as a limitation of our review:

“Fifth, our decision to exclude studies with sample sizes less than 40 may have excluded studies that reported on associations with rarer outcomes, such as ART use and HIV viral suppression.” Lines 510–512, Results

9. Reviewer: More detail is needed regarding the use of AI-enabled screening and full-text review. Most importantly, was this supervised by the authors and checked for accuracy?

Response: We apologise for the confusion, as the name of the systematic review software we used contains “AI”, which is misleading. Rayyan AI added AI screening and data extraction capabilities after we began using the software, hence the name change from “Rayyan” to “Rayyan AI”. To clarify, we did not use AI in the screening and data extraction of our systematic review. The following text has been added to the methods:

“Rayyan AI is a browser-based systematic review software, which allows users to highlight keywords for inclusion and exclusion, and streamlines the screening process by automatically flagging double-screened studies with conflicting decisions. Rayyan also has artificial intelligence capabilities which were not used for our review.” Lines 154–158, Methods

10. One of the biggest problems in global research in violence against women is the heterogenous nature of its measurement. How did authors deal with this? Which measures were used to assess violence? Were behavior or incident-based scales (beaten, kicked, etc.) assessed separately to global scales (e.g., "have you experienced violence")? More information on the measurement of violence and how authors analyzed the exposure is needed.

Response: We thank the reviewer for this comment and agree that the heterogeneity in the measurements of violence is a challenge when conducting reviews such as ours. To deal with this, we first categorised violence into multiple categories (physical, sexual, physical and/or sexual, other). Next, we classified violence exposures into whether they were recent (within the past year) or lifetime (greater than one year ago). For the example that you provided, “beaten, kicked, etc” would be categorised as physical violence and “have you experienced violence” would be categorised as physical and/or sexual. However, we recognise that these categorisations do not reflect the intensity of violence, which as the reviewer has pointed out, is a limitation both of our study and of the literature. It would be ideal to be able to conduct subgroup analyses depending on the intensity of violence, but most studies did not contain enough information for this to possible. Instead, we have added the following limitation:

“Third, there was heterogeneity in the measurement and definition of violence and some HIV outcomes across studies, which affected the comparability and synthesis of results. Whilst we conducted pooled analyses stratified by violence type, there were still variations in the definitions of violence within each strata. An important limitation of how violence is often measured in quantitative studies is the difficulty of measuring the nature and intensity of violent experiences, and so one person’s minor violence may be another person’s moderate violence. Consequently, our analysis risks homogenising minor and severe experiences of violence. Further, most studies did not account for other factors that may influence the relationship between violence and HIV outcomes, such as the perpetrator of violence, the type or frequency of violent experiences, and the timeframe over which violence occurred.” Lines 494–504, Limitations

11. Some reassurance that including both unadjusted and adjusted estimates together would be appreciated- I am not sure if this is common practice, but if so, a citation referencing it would be helpful

Response: We chose this approach based on guidance from Chapter 24 of the Cochrane Handbook for Systematic Reviews of Interventions on “Including non-randomized studies on intervention effects”, in which they recommend “If both unadjusted and adjusted intervention effects are reported, then adjusted effects should be preferred.”. We have now updated the manuscript to include a citation to these guidelines. Further, we included separate analyses of unadjusted and adjusted estimates in our sub-group/sensitivity analyses in the supplementary material, in which our overall findings remained unchanged.

12. The assessment of study quality appears quite rudimentary. Why did authors choose this method instead of one of the many comprehensive study quality assessments available?

Response: Because of the nature of the studies eligible for our review, we felt that some of the domains of the Newcastle-Ottawa scale were not applicable. For example, ascertaining violence exposure is challenging, and no included study used a validated tool. Therefore, all studies would be penalised for this domain. Similarly, we excluded studies with self-reported HIV status and viral suppression, and only included those with confirmatory test results, thus all studies for these outcomes would receive full marks. For HIV testing and ART use, basing our assessment of the outcome on laboratory testing and clinical evidence would have penalised all included studies, given the challenges assessing these outcomes. Consequently, we felt that the representativeness, temporality of exposure and outcome, and adjustment for confounding were the most relevant domains for our review, as they directly address the major methodological variations between studies that influenced study quality.

We have updated the description of the quality assessment with the following text:

“Study quality was assessed for each exposure-outcome estimate pair across three domains; study representativeness (with a score given for using representativeness recruitment approaches, including RDS, time-location or venue-based sampling, or recruitment through multiple, diverse sources), study design (longitudinal vs. cross-sectional) and whether estimates were adjusted or not (adjusted vs. unadjusted). These three domains were selected because they captured the primary sources of methodological variation across included studies. Other Newcastle-Ottawa scale domains, such as ascertainment of exposure, were not used because they would have penalised all studies equally, given that violence exposure cannot be verified against a secure record.” Lines 212–220, Methods

We would also like to draw attention to Appendix 7, in which we provide a detailed description of the study quality assessment.

**Results**

13. What does UPSOV stand for? I'm not sure I saw the initial definition.

Response: UPSOV was an acronym that we removed because we felt it was difficult for readers to interpret, and was unintentionally left in the submitted draft. It has now been removed, and we thank the reviewer for their attention to detail.

14. In general, the results are presented cleanly and straightforwardly.

Response: We thank the reviewer for this comment.

**Discussion**

15. The authors focus on the nature of forced sex and its role in HIV outcomes in those exposed to sexual violence. While mental health is mentioned, there are other indirect pathways through which sexual violence and HIV are related (e.g., post-traumatic stress). Unpacking these a bit more would be helpful

Response: We have added an additional reference that provides evidence for the association between PTSD and depression in the paragraph that discusses the role of mediators in the pathway from sexual violence to HIV outcomes:

“Experiences of violence are also associated with worse mental health outcomes such as depression and PTSD^45,46^, and injecting drug use^47,48^, which in turn are associated with HIV risk behaviours such as reduced condom use and having more sexual partners.^40^” Lines 430–433, Discussion

16. The authors may also want to include more discussion of primary prevention methods of reducing violence and its connection to HIV transmission- community mobilization is one, but working with men and boys to alter social norms around sex work, women's autonomy, etc. can also be helpful. Admittedly, the evidence for this is thin, but some interventions (e.g., Project H) have shown promise. Overall, a more holistic discussion of intervention approaches would be appreciated

Response: In response to this comment and a comment from another reviewer, we now have two paragraph on interventions for violence-reduction interventions:

“A key challenge for designing and implementing interventions that reduce violence among FSWs is the limited evidence base.^11^ However, there are interventions that have been demonstrated to be effective in reducing violence. For instance, community empowerment and peer group membership has been shown to reduce experiences of violence among FSWs^12-16^, increase their capacity to exercise autonomy and self-protection^13,15,16^, and potentially reduce HIV incidence among FSWs.^17,18^ Interventions focusing on social and structural contexts such as public health messaging, policy advocacy and education for law enforcement have also been shown to reduce experiences of violence among FSWs.^19-22^ In contrast, fewer interventions have focused on reducing behaviours that are associated with a higher likelihood of experiencing violence, although two studies have found evidence that interventions to reduce substance use can be effective at reducing violence.^23,24^ This evidence suggests that structural interventions should combine advocacy, community mobilisation and police education to reduce violence among FSWs. Continued investment in comprehensive, community-driven interventions is therefore required to address the complex factors contributing to violence among FSWs, which should then improve their HIV outcomes and promote their health and human rights.

While these interventions can reduce violence experienced by FSWs, they largely mitigate risk within existing social conditions rather than addressing the patriarchal, institutional, cultural, and political systems that produce it.^66^ Consequently, there is growing recognition that sustainable reductions in violence require interventions that promote systemic change alongside targeted programmes for FSWs.^67^ Evaluating such structural interventions is challenging, particularly in low-income settings,^68^ although evidence from violence-reduction interventions among women and girls demonstrates the potential of interventions which seek to transform gender norms and unequal power relations. These include community mobilisation initiatives, school- and community-based programmes that challenge gender norms and attitudes that condone violence^69-71^, and economic empowerment interventions, such as microfinance^72^ and vocational training.^73^ Multisectoral programmes that engage multiple stakeholders appear to be the most effective, as they simultaneously address gender inequality, women's economic dependence, and other structural drivers of violence.^68^ Although rarely implemented specifically among FSWs, they highlight the importance of embedding FSW-focused interventions within wider efforts to transform the social and institutional conditions that sustain violence.” Lines 457–485

# References

1.	Joint United Nations Programme on HIV/AIDS (UNAIDS). 90-90-90 An ambitious treatment target to help end the AIDS epidemic. Geneva; 2014.

2.	Afshari A, Wetterslev J. When may systematic reviews and meta-analyses be considered reliable? European Journal of Anaesthesiology| EJA. 2015;32(2):85-7.

3.	Turner RM, Bird SM, Higgins JP. The impact of study size on meta-analyses: examination of underpowered studies in Cochrane reviews. PLoS One. 2013;8(3):e59202.

4.	Sterne JAC, Egger M, Smith GD. Investigating and dealing with publication and other biases in meta-analysis. BMJ. 2001;323(7304):101-5.

5.	Shi L, Lin L. The trim-and-fill method for publication bias: practical guidelines and recommendations based on a large database of meta-analyses. Medicine (Baltimore). 2019;98(23):e15987.

6.	Barreto D, Deering K, Krüsi A, Ranville F, Safford H, Pooyak S, et al. HIV disclosure without consent linked to increased violence against women living with HIV in a Canadian setting. AIDS Care. 2024;36(1):98-106.

7.	Ojeaburu SO, Dorward J, Violette LR, Gibbs A, Shozi H, Sookrajh Y, et al. Intimate Partner Violence and HIV Outcomes Among Women Living with HIV in Durban, South Africa. AIDS and Behavior. 2024;28(7):2247-57.

8.	Rock A, McNaughton Reyes HL, Go V, Maman S, Perez M, Donastorg Y, et al. Relationships Between Stigma and Intimate Partner Violence Among Female Sex Workers Living With HIV: Social and Economic Exclusion. Violence Against Women. 2023;29(11):1971-97.

9.	Jewkes R, Morrell R, Sikweyiya Y, Dunkle K, Penn-Kekana L. Men, Prostitution and the Provider Role: Understanding the Intersections of Economic Exchange, Sex, Crime and Violence in South Africa. PLOS ONE. 2012;7(7):e40821.

10.	Lefebvre C, Glanville J, Briscoe S, Littlewood A, Marshall C, Metzendorf MI, et al. Searching for and selecting studies. Cochrane handbook for systematic reviews of interventions. 2019:67-107.

11.	Decker MR, Lyons C, Guan K, Mosenge V, Fouda G, Levitt D, et al. A Systematic Review of Gender-Based Violence Prevention and Response Interventions for HIV Key Populations: Female Sex Workers, Men Who Have Sex With Men, and People Who Inject Drugs. Trauma, Violence, & Abuse. 2022;23(2):676-94.

12.	Onoja AJ, Sanni FO, Shaibu J, Onoja S, Oguche D, Adamu I, et al. Baseline and postintervention assessment of sexual violence and condom use among female sex workers in a semiurban African community. Asian Journal of Social Health and Behavior. 2020;3(3):124-9.

13.	Beattie TS, Mohan HL, Bhattacharjee P, Chandrashekar S, Isac S, Wheeler T, et al. Community mobilization and empowerment of female sex workers in Karnataka State, South India: associations with HIV and sexually transmitted infection risk. American journal of public health. 2014;104(8):1516-25.

14.	Beattie TS, Bhattacharjee P, Isac S, Mohan HL, Simic-Lawson M, Ramesh BM, et al. Declines in violence and police arrest among female sex workers in Karnataka state, south India, following a comprehensive HIV prevention programme. Journal of the International AIDS Society. 2015;18(1):20079.

15.	Bhattacharjee P, Prakash R, Pillai P, Isac S, Haranahalli M, Blanchard A, et al. Understanding the role of peer group membership in reducing HIV-related risk and vulnerability among female sex workers in Karnataka, India. AIDS care. 2013;25(sup1):S46-S54.

16.	Punyam S, Pullikalu RS, Mishra RM, Sandri P, Mutupuru BP, Kokku SB, et al. Community advocacy groups as a means to address the social environment of female sex workers: a case study in Andhra Pradesh, India. J Epidemiol Community Health. 2012;66(Suppl 2):ii87-ii94.

17.	Kerrigan D, Kennedy CE, Morgan-Thomas R, Reza-Paul S, Mwangi P, Win KT, et al. A community empowerment approach to the HIV response among sex workers: Effectiveness, challenges, and considerations for implementation and scale-up. The Lancet. 2015;385(9963):172 EP - 85.

18.	Kerrigan D, Mbwambo J, Likindikoki S, Beckham S, Mwampashi A, Shembilu C, et al. Project Shikamana: Baseline findings from a community empowerment-based combination HIV prevention trial among female sex workers in iringa, tanzania. Journal of Acquired Immune Deficiency Syndromes. 2017;74:S60-S8.

19.	Beattie TSH, Bhattacharjee P, Ramesh BM, Gurnani V, Anthony J, Isac S, et al. Violence against female sex workers in Karnataka state, south India: impact on health, and reductions in violence following an intervention program. BMC public health. 2010;10(100968562):476.

20.	Reza-Paul S, Lorway R, O'Brien N, Lazarus L, Jain J, Bhagya M, et al. Sex worker-led structural interventions in India: a case study on addressing violence in HIV prevention through the Ashodaya Samithi collective in Mysore. Indian J Med Res. 2012;135(1):98-106.

21.	Bhattacharjee P, Morales GJ, Kilonzo TM, Dayton RL, Musundi RT, Mbole JM, et al. Can a national government implement a violence prevention and response strategy for key populations in a criminalized setting? A case study from Kenya. Journal of the international AIDS society. 2018;21:e25122.

22.	Dasgupta S. Violence in commercial sex work: A case study on the impact of violence among commercial female sex workers in India and strategies to combat violence. Violence against women. 2021;27(15-16):3056-73.

23.	Wechsberg WM, Zule WA, Luseno WK, Kline TL, Browne FA, Novak SP, et al. Effectiveness of an adapted evidence-based woman-focused intervention for sex workers and non-sex workers: the Women's Health CoOp in South Africa. Journal of Drug Issues. 2011;41(2):233-52.

24.	Parcesepe AM, Kelly L, Martin SL, Green S, Sinkele W, Suchindran C, et al. The impact of an alcohol harm reduction intervention on interpersonal violence and engagement in sex work among female sex workers in Mombasa, Kenya: Results from a randomized controlled trial. Drug and alcohol dependence. 2016;161:21-8.

25.	UN Women. RESPECT Women: Preventing violence against women. 2019.

26.	Michau L, Horn J, Bank A, Dutt M, Zimmerman C. Prevention of violence against women and girls: lessons from practice. The Lancet. 2015;385(9978):1672-84.

27.	Ellsberg M, Arango DJ, Morton M, Gennari F, Kiplesund S, Contreras M, et al. Prevention of violence against women and girls: what does the evidence say? The Lancet. 2015;385(9977):1555-66.

28.	Gupta AK, Santhya KG. Promoting Gender Egalitarian Norms and Practices Among Boys in Rural India: The Relative Effect of Intervening in Early and Late Adolescence. Journal of Adolescent Health. 2020;66(2):157-65.

29.	Vaillant J, Koussoubé E, Roth D, Pierotti R, Hossain M, Falb KL. Engaging men to transform inequitable gender attitudes and prevent intimate partner violence: a cluster randomised controlled trial in North and South Kivu, Democratic Republic of Congo. BMJ glob health. 2020;5(5):e002223.

30.	Keller J, Mboya BO, Sinclair J, Githua OW, Mulinge M, Bergholz L, et al. A 6-week school curriculum improves boys’ attitudes and behaviors related to gender-based violence in Kenya. Journal of interpersonal violence. 2017;32(4):535-57.

31.	Kim JC, Watts CH, Hargreaves JR, Ndhlovu LX, Phetla G, Morison LA, et al. Understanding the impact of a microfinance-based intervention on women’s empowerment and the reduction of intimate partner violence in South Africa. American journal of public health. 2007;97(10):1794-802.

32.	Bandiera O, Buehren N, Burgess R, Goldstein M, Gulesci S, Rasul I, et al. Empowering adolescent girls: evidence from a randomized control trial in Uganda: World Bank Washington, DC; 2012.

33.	Joint United Nations Programme on HIV/AIDS (UNAIDS). UNAIDS DATA 2023. Geneva; 2023.

34.	Atuhaire L, Adetokunboh O, Shumba C, Nyasulu PS. Effect of community-based interventions targeting female sex workers along the HIV care cascade in sub-Saharan Africa: a systematic review and meta-analysis. Systematic Reviews. 2021;10(1):137.

35.	Mountain E, Pickles M, Mishra S, Vickerman P, Alary M, Boily MC. The HIV care cascade and antiretroviral therapy in female sex workers: implications for HIV prevention. Expert Rev Anti Infect Ther. 2014;12(10):1203-19.

36.	Rhodes T, Simić M, Baroš S, Platt L, Žikić B. Police violence and sexual risk among female and transvestite sex workers in Serbia: qualitative study. BMJ. 2008;337:a811.

37.	Decker MR, Crago AL, Chu SKH, Sherman SG, Seshu MS, Buthelezi K, et al. Human rights violations against sex workers: Burden and effect on HIV. The Lancet. 2015;385(9963):186 EP - 99.

38.	Leddy AM, Underwood C, Decker MR, Mbwambo J, Likindikoki S, Galai N, et al. Adapting the Risk Environment Framework to Understand Substance Use, Gender-Based Violence, and HIV Risk Behaviors Among Female Sex Workers in Tanzania. AIDS and behavior. 2018;22(10):3296-306.

39.	Shannon K, Strathdee SA, Goldenberg SM, Duff P, Mwangi P, Rusakova M, et al. Global epidemiology of HIV among female sex workers: influence of structural determinants. The Lancet. 2015;385(9962):55-71.

40.	Deering KN, Amin A, Shoveller J, Nesbitt A, Garcia-Moreno C, Duff P, et al. A systematic review of the correlates of violence against sex workers. American journal of public health. 2014;104(5):e42-e54.

41.	Arum C, Fraser H, Artenie AA, Bivegete S, Trickey A, Alary M, et al. Homelessness, unstable housing, and risk of HIV and hepatitis C virus acquisition among people who inject drugs: a systematic review and meta-analysis. Lancet Public Health. 2021;6(5):e309-e23.

42.	Stone J, Fraser H, Lim AG, Walker JG, Ward Z, MacGregor L, et al. Incarceration history and risk of HIV and hepatitis C virus acquisition among people who inject drugs: a systematic review and meta-analysis. The Lancet infectious diseases. 2018;18(12):1397-409.

43.	Artenie A, Stone J, Fraser H, Stewart D, Arum C, Lim AG, et al. Incidence of HIV and hepatitis C virus among people who inject drugs, and associations with age and sex or gender: a global systematic review and meta-analysis. The Lancet Gastroenterology & Hepatology. 2023.

44.	Artenie A, Trickey A, Looker KJ, Stone J, Lim AG, Fraser H, et al. Global, regional, and national estimates of hepatitis C virus (HCV) infection incidence among people who inject drugs and number of new annual HCV infections attributable to injecting drug use: a multi-stage analysis. The Lancet Gastroenterology & Hepatology. 2025;10(4):315-31.

45.	Beattie TS, Smilenova B, Krishnaratne S, Mazzuca A. Mental health problems among female sex workers in low- and middle-income countries: A systematic review and meta-analysis. PLOS Medicine. 2020;17(9):e1003297.

46.	Coetzee J, Buckley J, Otwombe K, Milovanovic M, Gray GE, Jewkes R. Depression and Post Traumatic Stress amongst female sex workers in Soweto, South Africa: A cross sectional, respondent driven sample. PLOS ONE. 2018;13(7):e0196759.

47.	Strathdee SA, West BS, Reed E, Moazan B, Azim T, Dolan K. Substance Use and HIV Among Female Sex Workers and Female Prisoners: Risk Environments and Implications for Prevention, Treatment, and Policies. JAIDS Journal of Acquired Immune Deficiency Syndromes. 2015;69.

48.	Ulibarri MD, Strathdee SA, Ulloa EC, Lozada R, Fraga MA, Magis-Rodriguez C, et al. Injection drug use as a mediator between client-perpetrated abuse and HIV status among female sex workers in two Mexico-US border cities. AIDS and behavior. 2011;15(1):179-85.
