# Supplementary material for: Impact of violence on HIV outcomes among female sex workers: A global systematic review and meta-analysis
Source: PLoS Med. 2026 Oct 21;23(10):e1004977. doi: 10.1371/journal.pmed.1004977 (PMC13712678; doi:10.1371/journal.pmed.1004977)
Supplement: Supplementary file 3 [file pmed.1004977.s005.docx]

***PLOS Medicine*:** “Impact of violence on HIV outcomes among female sex workers: A global systematic review and meta-analysis.”

Dear Editors,

We would like to thank the reviewers for their detailed and insightful comments on our manuscript. Our responses to each comment are outlined below, and we have made changes to the manuscript where appropriate. Line numbers refer to the tracked version of the document.

# Academic Editor

1. Editor: The abstract and manuscript text are inconsistent in terms of some of the numbers.

Abstract: search ended February 10, 2025, there were 91 studies, 221 estimates, 186,613 participants, and 39 countries.

Methods: search ended June 9, 2026

Results: reported 94 studies and 226 estimates

I think the inconsistency is just because the abstract was not updated in the revision, but it probably should be updated.

Response: The editor is correct that we did not correctly update the title and abstract, this has now been corrected.

2. Editor: Related to the above: in the revised Results the authors say that 90 studies met inclusion criteria, 5 were excluded as duplicates, then 9 identified through reference searching. But in the PRISMA diagram there were 85 studies eligible for review then 9 identified through reference searching.

Response: This information is correct, but the PRISMA diagram does not intuitively follow the reporting of results, hence the confusion. To clarify, 90 studies were eligible, but 5 of these reported duplicate estimates from the same cohort, and so were not eligible for the review, leaving us with 85 studies. The flow diagram has now been updated to align with the way it is presented in the results.

3. Editor: In the initial submission, the authors stated that UNAIDS "supported the conceptualisation of the project and reviewed the final draft before submission." But in the revised submission, the statement simply says that funders had "no role in study design, data collection and analysis, decision to publish, or preparation of the manuscript." The reason for the change is unclear to me, especially since several coauthors are UNAIDS staff.

Response: We changed the financial disclosure after receiving clarification from a PLOS Medicine Journal Assistant, as we were unsure on how this was meant to be reported. We have discussed this issue with the managing editor and have now updated it to:

“JD and SAK received funding from the GW4 BioMed MRC Doctoral Training Partnership. KAM and JA received funding from a Bristol Postgraduate Research Scholarship. JD, KAM, SAK, JS, AA and PV received funding from The Joint United Nations Programme on HIV/AIDS (UNAIDS) (grant code 2024/1464836). AT, JS, AA and PV received funding from the Wellcome Trust (grant code 226619/Z/22/Z). KS and VB are employed by the funders (UNAIDS), and supported the conceptualisation of the project and reviewed the final draft before submission. The funders had no other role in study design.”

# Requests from Editors:

1. Editor: Please ensure that the metadata aligns with the manuscript.

Response: We have made sure that the metadata aligns with the manuscript.

2. Editor: Please consider moving the funnel plots to the main manuscript.

Response: Given that there are 43 funnel plots included in the supplementary material, we have included the six funnel plots for recent and lifetime exposure to all forms of violence and prevalent HIV infection, and left the remaining 37 figures in the Appendices, although we can add the remaining funnel plots if the editors feel this is appropriate.

3. Editor: Please note that ranges or intervals should commas and not dashes.

Response: We have changed the commas to dashes.

4. Editor: Please note the requirement below for a DOI.

Response: We have included the following DOI to access the relevant files: https://doi.org/10.5281/zenodo.22809473.

5. Editor: Please note that we require the inclusion of an Author Summary.

Response: We have included an Author Summary.

# General editorial requests

1. Editor: At this stage, we ask that you include a short, non-technical Author Summary of your research to make findings accessible to a wide audience that includes both scientists and non-scientists. The Author Summary should immediately follow the Abstract in your revised manuscript. This text is subject to editorial change and should be distinct from the scientific abstract. Ideally each sub-heading should contain 2-3 single sentence, concise bullet points containing the most salient points from your study.

Response: We have included an Author Summary with sub-headings that contain single sentence bullet points.

2. Editor: In the final bullet point of the Author Summary, ‘What Do These Findings Mean?’, please include the main limitations of the study in non-technical language.

Response: We have added the following text describing the main limitations of our study:

“The main limitations of our study were the heterogeneity in pooled effects, and that most of the included studies were cross-sectional.”

3. Editor: Please ensure that the Introduction ends with a clear description of the study question or hypothesis.

Response: Our introduction ends with the following sentence:

“We therefore undertook a global systematic review and meta-analysis to characterise the effects of violence on specific HIV outcomes (HIV testing, ART use, ART adherence, viral suppression) among FSWs.” Lines 119–122, Introduction

4. Editor: Please ensure that all abbreviations are defined at first use throughout the text.

Response: We have done this.

5. Editor: Please confirm that all numbers presented in the abstract are present and identical to numbers presented in the main manuscript text [see relevant comments from the Academic Editor].

Response: We have updated the abstract to align with the figures and the main manuscript text.

6. Editor: Please include a statement regarding use or non-use of AI tools and technologies at the end of the Methods. Please specify the name(s) and version(s) of any tool(s) used and a description of their use, including how the AI-assisted or generated content was evaluated by authors, and a clear statement of which aspects of the study, article contents, data, or supporting files were affected or generated by AI tool usage. Please also include a statement specifying who retains responsibility for the final manuscript. See our guidelines for more information: <https://journals.plos.org/plosmedicine/s/ethical-publishing-practice#loc-artificial-intelligence-tools-and-technologies>.

Response: We have added the following text to the end of the methods section:

“Claude.ai was used to assist with debugging analytical code. No artificial intelligence tools were used to write and revise the manuscript.” Lines 238–239, Methods

7. Editor: Please remove the 'conclusions' subheading from the discussion. Please also remove any other subheadings from the discussion.

Response: We have done this.

8. Editor: Please report statistical information as follows to improve clarity for the reader ""22% (95% CI [13,28]; p</=)"".

Response: We have used this formatting for the 95% CIs.

9. Editor: Please separate upper and lower bounds with commas instead of hyphens as the latter can be confused with reporting of negative values.

Response: We have done this.

10. Editor: Please repeat statistical definitions (HR, CI etc.) for each set of parentheses.

Response: We have done this.

11. Editor: The funding statement should include: specific grant numbers, initials of authors who received each award, URLs to sponsors’ websites. Also, please state whether any sponsors or funders (other than the named authors) played any role in study design, data collection and analysis, the decision to publish, or preparation of the manuscript. If they had no role in the research, include this sentence: “The funders had no role in study design, data collection and analysis, decision to publish, or preparation of the manuscript.

Response: This aligns with a previous comment from the academic editor, so we have copied our response below:

We changed the financial disclosure after receiving clarification from a PLOS Medicine Journal Assistant, as we were unsure on how this was meant to be reported. We have discussed this issue with the managing editor and have now updated it to:

“JD and SAK received funding from the GW4 BioMed MRC Doctoral Training Partnership. KAM and JA received funding from a Bristol Postgraduate Research Scholarship. JD, KAM, SAK, JS, AA and PV received funding from The Joint United Nations Programme on HIV/AIDS (UNAIDS) (grant code 2024/1464836). AT, JS, AA and PV received funding from the Wellcome Trust (grant code 226619/Z/22/Z). KS and VB are employed by the funders (UNAIDS), and supported the conceptualisation of the project and reviewed the final draft before submission. The funders had no other role in study design.”

12. Editor: All authors must declare their relevant competing interests per the PLOS policy, which can be seen here: <https://journals.plos.org/plosmedicine/s/competing-interests> For authors with ties to industry, please indicate whether any of the interests has a financial stake in the results of the current study. Please ensure that all authors have completed the questionnaire they received by email.

Response: No authors have competing interests to declare.

13. Editor: PLOS defines the “minimal data set” to consist of the data set used to reach the conclusions drawn in the manuscript with related metadata and methods, and any additional data required to replicate the reported study findings in their entirety. Authors do not need to submit their entire data set, or the raw data collected during an investigation. Please submit the following data:

The values behind the means, standard deviations and other measures reported; The values used to build graphs; The points extracted from images for analysis."

"* The Data Availability Statement (DAS) requires revision. For each data source used in your study:

a) If the data are freely or publicly available, note this and state the location of the data: within the paper, in Supporting Information files, or in a public repository (include the DOI or accession number).

b) If the data are owned by a third party but freely available upon request, please note this and state the institutional owner of the data set and contact information for data requests (institutional web or email address). Note that a study author cannot be the contact for the data.

c) If the data are not freely available, please describe briefly the ethical, legal, or contractual restriction that prevents you from sharing it. Please also include an appropriate institutional contact (institutional web or email address) for inquiries (again, this cannot be a study author).

Response: The data and code are available to download and analyse via a DOI. This is described in the methods:

“The finalised data extraction and analysis code are publicly available at https://doi.org/10.5281/zenodo.22809473.”, Lines 239–240, Methods

14. Editor: For studies in which a novel model is central to the manuscript's findings, authors are responsible for providing the source code needed to replicate the study's findings in a repository (such as GitHub, SourceForge or Bitbucket) or a cloud computing service (such as Code Ocean). Please include a citable digital object identifier (DOI) or other persistent identifier of the code (such as through linkage with Zenodo). Protection of authors’ intellectual property will not be cause for exception. Please explain in the manuscript’s Data Availability Statement how readers can access the shared code.

Response: The data and code are available to download and analyse via the GitHub of the first author. This is described in the methods:

“The finalised data extraction and analysis code are publicly available at https://doi.org/10.5281/zenodo.22809473.”, Lines 239–240, Methods

15. Editor: Please include the statement on code availability in the data availability statement. Please see guidance here:https://journals.plos.org/plosmedicine/s/code-sharing-guidance#loc-data-availability-statement-examples

Response: The data and code are available to download and analyse via the GitHub of the first author. This is described in the methods:

“The finalised data extraction and analysis code are publicly available at https://doi.org/10.5281/zenodo.22809473.”, Lines 239–240, Methods

16. Editor: Please define all elements of box plots in the figure caption - center line, box limits and whiskers.

Response: Our submission did not include box plots.

17. Editor: Please provide titles and legends for all figures and tables (including those in Supporting Information files). Please define all acronyms used in each figure or table in its corresponding legend.

Response: We have done this.

18. Editor: Please include an Acknowledgments section in your manuscript. The Acknowledgements should not include funder information.

Response: We have added an Acknowledgements section with the following text:

“We would like to acknowledge support from The Joint United Nations Programme on HIV/AIDS (UNAIDS), the Wellcome Trust, and the GW4 BioMed MRC Doctoral Training Partnership.”

19. Editor: Please report your SR/MA according to the PRISMA guidelines provided at the EQUATOR site.

http://www.equator-network.org/reporting-guidelines/prisma/

Please provide the completed PRISMA checklist as Supporting Information.

When completing the checklist, please use section and paragraph numbers, rather than page numbers.

Please add the following statement, or similar, to the Methods: ""This study is reported as per the Preferred Reporting Items for Systematic Reviews and Meta-Analyses (PRISMA) guideline (S1 Checklist)."""

Response: We have done this and the following text appears in the methods of the manuscript:

“This systematic review and meta-analysis is reported according to the 2020 preferred reporting items for systematic reviews and meta-analyses (PRISMA) checklist^20^, following a registered study protocol PROSPERO (ID: CRD42024531539). The review was conducted in unison with another review, which aimed to estimate the effects of stigma and discrimination on the same HIV outcomes among FSWs. The completed PRISMA checklist is included in Appendix 2.” Lines 124–129, Methods

20. Editor: * Please provide the beginning and end dates of your search.

Response: We do this in the Abstract:

“We searched MEDLINE, Embase, and PsycINFO databases for studies published from January 1^st^, 2010 to June 9^th^, 2026 assessing the impact of violence on HIV outcomes among FSW, without restriction to language and study design.” Lines 31–32, Abstract

and in the Methods of the main manuscript:

“We searched MEDLINE, Embase, and PsycINFO for published studies from January 1^st^, 2010 to June 9^th^, 2026, without language restrictions.” Lines 131–132, Methods

21. Editor: * Please report your abstract according to PRISMA for abstracts, following the PLOS Medicine abstract structure (Background, Methods and Findings, Conclusions): <https://doi.org/10.1371/journal.pmed.1001419>

Response: We have done this.

# Reviewer 2

Overall, the authors did a great job on this revision. I only have a few comments.

Response: We thank the reviewer for their kind and for their considered review.

1. Reviewer: Despite the editor's request to use the 2025 framework, the authors still used the PRISMA 2020 framework. The authors may want to update the framework.

Response: We used the 2020 PRISMA checklist as this is the current standard as per their website (<https://www.prisma-statement.org>).

2. Reviewer: The authors stated that search was updated to June 9, 2026, but the abstract still said Feb 10, 2025. Please make these dates consistent.

Response: We thank the reviewer for noticing this mistake, and have updated the error in the abstract.

3. Reviewer: The authors have substantially improved this manuscript, but causal wording still remains. For instance, "experiencing violence may increase HIV transmission risk and worsen HIV treatment outcomes" is a casual inference. The authors may want to change such statements as "experiencing violence is associated with higher HIV prevalence and poorer treatment outcomes".

Response: We have changed the causal wording in this sentence to now be:

“Experiencing violence is associated with greater odds of prevalent HIV infections, and poorer HIV treatment outcomes among FSW.” Lines 56–58, Abstract

# Reviewer 3

I thank the authorship team for their thoughtful responses to my critiques. Their attention to detail and grace in receiving these comments should be applauded. There are a few small issues (e.g., stratum vs strata) that will be corrected in the next rounds of review, but I feel the paper is now ready for copyediting.

Response: We thank the reviewer for their kind comments and for their considered review.
