# Supplementary material for: Impact of violence on HIV outcomes among female sex workers: A global systematic review and meta-analysis
Source: PLoS Med. 2026 Oct 21;23(10):e1004977. doi: 10.1371/journal.pmed.1004977 (PMC13712678; doi:10.1371/journal.pmed.1004977)
Supplement: Peer review history

# Editor report: Decision Letter 0

**DOI:** 10.1371/journal.pmed.1004977.r001

**Authors:** Alison Farrell

20 Feb 2026

Dear Dr Dawe,

Thank you for submitting your manuscript entitled "Impact of violence on HIV outcomes among female sex workers: A global systematic review and meta-analysis" for consideration by PLOS Medicine.

Your manuscript has now been evaluated by the PLOS Medicine editorial staff, and I am writing to let you know that we would like to send your submission out for external peer review.

However, before we can send your manuscript to reviewers, we need you to complete your submission by providing the metadata that is required for full assessment. To this end, please login to Editorial Manager where you will find the paper in the 'Submissions Needing Revisions' folder on your homepage. Please click 'Revise Submission' from the Action Links and complete all additional questions in the submission questionnaire.

**Please note that we require at submission the inclusion of related manuscripts so that the editors can assess the degree of possible overlap. Therefore we ask that you please include the related review estimating the effects of stigma and discrimination on HIV outcomes among female sex workers.

For clinical studies, please upload a copy of your trial study protocol as a supporting information file. The study protocol should be the version submitted for approval to the institutional review board or ethics committee, should include any amendments to the study protocol, as well as the date of their approval by the institutional review or ethics committee. Please also detail any deviations from the study protocol in the Methods section of your manuscript. The editors will consider the protocol and study conduct prior to a final decision for external review.

Please re-submit your manuscript within two working days, i.e. by Feb 24 2026 11:59PM.

Login to Editorial Manager here: https://www.editorialmanager.com/pmedicine

Once your full submission is complete, your paper will undergo a series of checks in preparation for peer review. Once your manuscript has passed all checks it will be sent out for review.

Feel free to email us at plosmedicine@plos.org if you have any queries relating to your submission.

Kind regards,

Alison Farrell, Ph.D.

Senior Editor

PLOS Medicine

# Peer review: Decision Letter 1

**DOI:** 10.1371/journal.pmed.1004977.r002

**Authors:** Alison Farrell

5 Jun 2026

Dear Dr Dawe,

Many thanks for submitting your manuscript "Impact of violence on HIV outcomes among female sex workers: A global systematic review and meta-analysis" (PMEDICINE-D-26-00594R1) to PLOS Medicine. The paper has been reviewed by subject experts and a statistician; their comments are included below and can also be accessed here: [LINK]

As you will see, the reviewers find the study interesting and have made suggestions to improve the clarity and presentation. After discussing the paper with the editorial team and an academic editor with relevant expertise, I'm pleased to invite you to revise the paper in response to the reviewers' comments. We plan to send the revised paper to some or all of the original reviewers, and we cannot provide any guarantees at this stage regarding publication.

Please note that we require the inclusion of an updated PRISMA 2025 checklist. Please also include sections rather than page numbers in the completed checklist.

Please clarify the rationale for the time period of the search.

Please indicate how articles that were not in English were handled, as your Methods indicate that the search was not restricted by language.

Please provide more details in the Methods on the assessments of bias and study quality.

Please include as a last sentence of the Methods and Findings section of the Abstract a statement on the limitations of the study methodology.

Please note that we expect searches to be updated to within 6 months of the time of submission.

Please include a point-by-point rebuttal to all reviewer and editor concerns upon resubmission.

In addition to these revisions, you may need to complete some formatting changes, which you will receive in a follow up email. A member of our team will be in touch with a set of requests shortly. If you do not receive a separate email within a few days, please assume that checks have been completed, and no additional changes are required.

When you upload your revision, please include a point-by-point response that addresses all of the reviewer and editorial points, indicating the changes made in the manuscript and either an excerpt of the revised text or the location (eg: page and line number) where each change can be found. Please also be sure to check the general editorial comments at the end of this letter and include these in your point-by-point response. When you resubmit your paper, please include a clean version of the paper as the main article file and a version with changes tracked as a marked-up manuscript. It may also be helpful to check the guidelines for revised papers at http://journals.plos.org/plosmedicine/s/revising-your-manuscript for any that apply to your paper.

We ask that you submit your revision by Jun 26 2026 11:59PM. However, if this deadline is not feasible, please contact me by email, and we can discuss a suitable alternative.

Don't hesitate to contact me directly with any questions (afarrell@plos.org).

Best regards,

Alison

Alison Farrell, Ph.D.

Senior Editor

PLOS Medicine

afarrell@plos.org

-----------------------------------------------------------

Comments from the reviewers:

Reviewer #1: The methods employ a variety very specific but not clearly justified choices. It is unclear what effect these have on the results .

These include

- [ ] The arbitrary date of January 1st, 2010, or later, for inclusion of articles.

- [ ] An unusual inclusion criterion: "could be used to estimate associations between any form of violence and one ormore selected HIV outcomes among FSWs," which appears broad and difficult to verify.

- [ ] A rule that 70% of the sample had to include female sex workers.

- [ ] A rule that studies had to have at least 40 participants.

-

The inclusion criteria about ability to "estimate associations" appears very broad and subjective. I wonder if there is a way that this could be articulated that is more transparent and objective.

The time frames for reports of violence and reports of set works do not necessarily align. It's possible that sex work may have occurred long before violence in the case of these.

The results say that three-quarters of the studies were considered representative. It's unclear how they judge the representativeness of the populations in the studies.

Overall, the methods appear to be solid and appropriate for the analysis. They used random effects. They assessed for heterogeneity as well as a publication bias. However, the results of these were not reassuring. There appears to be evidence of publication bias from the funnel plots, I am not sure if the funnel plots were given in the paper. In addition, in many cases, the heterogeneity is quite high.

I feel that these issues should be discussed with more nuance in the discussion section. And investigated by reporting the effects by the subgroups or examining if these are explained by meta regression.

That there's particular need for nuance around the association between prevalent HIV and violence, the cross-sectional nature of that association makes it difficult to infer the directionality of the association or the possibility that it's due to unmeasured confounding.

Reviewer #2: Reviewer Comments

This manuscript presents a comprehensive and timely systematic review and meta-analysis examining the association between violence and HIV outcomes among female sex workers (FSWs). The use of multilevel random-effects models and robust variance estimation is methodologically appropriate given the dependence among effect sizes. Overall, the manuscript is well-written and addresses an important structural determinant of HIV risk. This is a strong and policy-relevant manuscript that advances understanding of structural determinants of HIV among FSWs. The authors may consider the following suggestions for improvement.

Major Comments

1. While the authors acknowledge that most included studies are cross-sectional, the interpretation of findings occasionally implies causal relationships (e.g., violence "increases" HIV risk). The language should be further tempered throughout the manuscript to emphasize associations rather than causal effects.

2. There is substantial heterogeneity in how violence and HIV outcomes are defined and measured across studies. Although the authors grouped violence types, further discussion is needed on how this variability may bias pooled estimates and limit comparability, particularly for "mixed" and "other" violence categories.

3. The analysis of HIV incidence, ART adherence, and viral suppression is based on a small number of studies. The manuscript would benefit from a clearer emphasis on the limited statistical power and cautious interpretation of these findings, especially where null results are reported.

4. The authors note potential publication asymmetry. However, the search strategy (requiring violence to appear in titles/abstracts) may systematically exclude null findings. This should be more explicitly framed as a potential source of selection bias and discussed in terms of its impact on pooled estimates.

Minor Comments

1.Briefly explain the data extraction/review software (Rayyan AI) in the method section.

2.Why did authors exclude studies with sample sizes less than 40? How is the cut-off determined? More rationales should be provided.

3.Clarify the definition and interpretation of "other violence," which appears highly heterogeneous and may obscure meaningful distinctions.

4.Provide more detail on quality assessment scoring and how it influenced interpretation (currently described briefly).

5.Consider adding a summary table of key covariates adjusted for in included studies.

6.Strengthen the policy/implementation implications by linking findings more explicitly to actionable intervention strategies.

Reviewer #3: Overall: This manuscript represents a robust attempt to categorise and summarise the evidence for associations between violence and HIV outcomes in FSWs. In general, the authors should be applauded for their work and straightforward presentation of the results. Most comments are minor, and I believe that with some revisions, the manuscript should be suitable for publication. I thank the authors for their work and look forward to citing this helpful study in my own research.

Abstract: The study lays out a coherent rationale and approach. It also raises a few questions that I assume will be answered in the course of the paper. It would be helpful to know whether the 37 countries were all LMICs and whether analyses by type of violence (physical, sexual, etc.) were completed.

Introduction:

-LMICs stands for "low- and middle-income" not "lower- "

-Please elaborate on the inequities that exist in the HIV cascade among FSWs.

-Overall, the Introduction feels a bit stunted. The rationale for the study is well described, but if word limits allow, I suggest elaborating throughout.

Methods

-Why did the authors choose these three databases (and not more or others?)

-Why did the authors choose to start their search in 2010? Some rationale for this choice is needed

-A requirement that 70% of a sample needed to "recently/ever engaged in sex work" is a broad inclusion criterion- some rationale for this rule is needed

-Similarly, why N=40 for the sample size rule? These are all defensible, but the authors need to provide such a defense

-More detail is needed regarding the use of AI-enabled screening and full-text review. Most importantly, was this supervised by the authors and checked for accuracy?

-One of the biggest problems in global research in violence against women is the heterogenous nature of its measurement. How did authors deal with this? Which measures were used to assess violence? Were behavior or incident-based scales (beaten, kicked, etc.) assessed separately to global scales (e.g., "have you experienced violence")? More information on the measurement of violence and how authors analyzed the exposure is needed.

-Some reassurance that including both unadjusted and adjusted estimates together would be appreciated- I am not sure if this is common practice, but if so, a citation referencing it would be helpful

-The assessment of study quality appears quite rudimentary. Why did authors choose this method instead of one of the many comprehensive study quality assessments available?

Results

-What does UPSOV stand for? I'm not sure I saw the initial definition.

-In general, the results are presented cleanly and straightforwardly.

Discussion

-The authors focus on the nature of forced sex and its role in HIV outcomes in those exposed to sexual violence. While mental health is mentioned, there are other indirect pathways through which sexual violence and HIV are related (e.g., post-traumatic stress). Unpacking these a bit more would be helpful

-The authors may also want to include more discussion of primary prevention methods of reducing violence and its connection to HIV transmission- community mobilization is one, but working with men and boys to alter social norms around sex work, women's autonomy, etc. can also be helpful. Admittedly, the evidence for this is thin, but some interventions (e.g., Project H) have shown promise. Overall, a more holistic discussion of intervention approaches would be appreciated

Any attachments provided with reviews can be seen via the following link: [LINK]

--------------------------------------------------------- ---

General editorial requests:

(Note: not all will apply to your paper, but please check each item carefully)

*We ask every co-author listed on the manuscript to fill in a contributing author statement, making sure to declare all competing interests. If any of the co-authors have not filled in the statement, we will remind them to do so when the paper is revised. If all statements are not completed in a timely fashion this could hold up the re-review process. If new competing interests are declared later in the revision process, this may also hold up the submission. Should there be a problem getting one of your co-authors to fill in a statement we will be in contact. Please do not add or remove authors without first discussing this with the handling editor. You can see our competing interests policy here: http://journals.plos.org/plosmedicine/s/competing-interests.

*Please upload any figures associated with your paper as individual TIF or EPS files with 300dpi resolution at resubmission; please read our figure guidelines for more information on our requirements: http://journals.plos.org/plosmedicine/s/figures. While revising your submission, we strongly recommend that you use PLOS's NAAS tool (https://ngplosjournals.pagemajik.ai/artanalysis) to test your figure files. NAAS can convert your figure files to the TIFF file type and meet basic requirements (such as print size, resolution), or provide you with a report on issues that do not meet our requirements and that NAAS cannot fix.

After uploading your figures to PLOS's NAAS tool - https://ngplosjournals.pagemajik.ai/artanalysis, NAAS will process the files provided and display the results in the "Uploaded Files" section of the page as the processing is complete.

If the uploaded figures meet our requirements (or NAAS is able to fix the files to meet our requirements), the figure will be marked as "fixed" above. If NAAS is unable to fix the files, a red "failed" label will appear above.

When NAAS has confirmed that the figure files meet our requirements, please download the file via the download option, and include these NAAS processed figure files when submitting your revised manuscript.

*Please ensure that the paper adheres to the PLOS Data Availability Policy (see http://journals.plos.org/plosmedicine/s/data-availability), which requires that all data underlying the study's findings be provided in a repository or as Supporting Information. For data residing with a third party, authors are required to provide instructions with contact information (web or email address) for obtaining the data. Please note that a study author cannot be the contact person for the data. PLOS journals do not allow statements supported by "data not shown" or "unpublished results." For such statements, authors must provide supporting data or cite public sources that include it.

*We expect all researchers with submissions to PLOS in which author-generated code underpins the findings in the manuscript to make all author-generated code available without restrictions upon publication of the work. In cases where code is central to the manuscript, we require the code to be made available as a condition of publication. Authors are responsible for ensuring that the code is reusable and well documented. Please make any custom code available, either as part of your data deposition or as a supplementary file. Please add a sentence to your data availability statement regarding any code used in the study, e.g. "The code used in the analysis is available from Github [URL] and archived in Zenodo [DOI link]" Please review our guidelines at https://journals.plos.org/plosmedicine/s/materials-software-and-code-sharing and ensure that your code is shared in a way that follows best practice and facilitates reproducibility and reuse. Because Github depositions can be readily changed or deleted, we encourage you to make a permanent DOI'd copy (e.g. in Zenodo) and provide the URL.

*Please clarify if any new code was generated for this manuscript. If new code was generated, please deposit it in a public repository and provide a DOI.

* Please disclose the use of any AI in drafting this manuscript or analyzing data (use, tool, version). If used, please include a statement on who verified the accuracy of the output and who retains responsibility for the final version.

*Please ensure that the study is reported according to the PRISMA 2025 guideline and include the completed PRISMA 2025 checklist as Supporting Information. When completing the checklist, please use section and paragraph numbers, rather than page numbers. Please add the following statement, or similar, to the Methods: "This study is reported as per PRISMA guideline (S1 Checklist)."

FORMATTING - GENERAL

*Abstract: Please structure your abstract using the PLOS Medicine headings (Background, Methods and Findings, Conclusions). Please combine the Methods and Findings sections into one section.

*At this stage, we ask that you include a short, non-technical Author Summary of your research to make findings accessible to a wide audience that includes both scientists and non-scientists. The Author Summary should immediately follow the Abstract in your revised manuscript. This text is subject to editorial change and should be distinct from the scientific abstract. Ideally each sub-heading should contain 2-3 single sentence, concise bullet points containing the most salient points from your study. In the final bullet point of 'What Do These Findings Mean?', please include the main limitations of the study in non-technical language. Please see our author guidelines for more information: https://journals.plos.org/plosmedicine/s/revising-your-manuscript#loc-author-summary.

*Please express the main results with 95% CIs as well as p values. When reporting p values please report as p<0.001 and where higher as the exact p value p=0.002, for example. Throughout, suggest reporting statistical information as follows to improve clarity for the reader "22% (95% CI [13%,28%]; p</=)". Please be sure to define all numerical values at first use.

*Please include page numbers and line numbers in the manuscript file. Use continuous line numbers (do not restart the numbering on each page).

*Please cite the reference numbers in square brackets. Citations should precede punctuation.

FIGURES AND TABLES

*Please provide titles and legends for all figures and tables (including those in Supporting Information files).

*Please define all abbreviations used in each figure/table (including those in Supporting Information files).

*Please consider avoiding the use of red and green in order to make your figure more accessible to those with color blindness.

SUPPLEMENTARY MATERIAL

*Please note that supplementary material will be posted as supplied by the authors. Therefore, please amend it according to the relevant comments outlined here.

*Please cite your Supporting Information as outlined here: https://journals.plos.org/plosmedicine/s/supporting-information

REFERENCES

*PLOS uses the numbered citation (citation-sequence) method and first six authors, et al.

*Please ensure that journal name abbreviations match those found in the National Center for Biotechnology Information (NCBI) databases (http://www.ncbi.nlm.nih.gov/nlmcatalog/journals), and are appropriately formatted and capitalised.

*Where website addresses are cited, please include the complete URL and specify the date of access (e.g. [accessed: 12/06/2023]).

*Please also see https://journals.plos.org/plosmedicine/s/submission-guidelines#loc-references for further details on reference formatting.

SYSTEMATIC REVIEWS & META-ANALYSES

*Please report your SR/MA according to the PRISMA guidelines provided at the EQUATOR site. http://www.equator-network.org/reporting-guidelines/prisma/. Please provide the completed PRISMA checklist as Supporting Information. When completing the checklist, please use section and paragraph numbers, rather than page numbers. Please add the following statement, or similar, to the Methods: "This study is reported as per the Preferred Reporting Items for Systematic Reviews and Meta-Analyses (PRISMA) guideline (S1 Checklist)."

*Abstract: Please report your abstract according to PRISMA for abstracts (https://doi.org/10.1371/journal.pmed.1001419) following the PLOS Medicine abstract structure (Background, Methods and Findings, Conclusions). Please ensure you provide dates of search, data sources, number of studies included, types of study designs included, eligibility criteria, and synthesis/appraisal methods.

*Please note that we expect searches to be updated to within 6 months of the time of submission.

# Author comment: Author response to Decision Letter 2

**DOI:** 10.1371/journal.pmed.1004977.r003

10 Jul 2026

Attachment Submitted filename: Reviewer comments_02072026.docx

# Peer review: Decision Letter 2

**DOI:** 10.1371/journal.pmed.1004977.r004

**Authors:** Alison Farrell

9 Sep 2026

Dear Dr. Dawe,

Thank you very much for re-submitting your manuscript "Impact of violence on HIV outcomes among female sex workers: A global systematic review and meta-analysis" (PMEDICINE-D-26-00594R2) for review by PLOS Medicine.

I have discussed the paper with my colleagues and the academic editor and it was also seen again by the 3 original reviewers. I am pleased to say that provided the remaining editorial and production issues are dealt with we are planning to accept the paper for publication in the journal.

Please ensure that you include a point-by-point rebuttal to all remaining reviewer and editor comments upon submission of your revised manuscript.

The remaining issues that need to be addressed are listed at the end of this email. Any accompanying reviewer attachments can be seen via the link below. Please take these into account before resubmitting your manuscript:

[LINK]

***Please note while forming your response, if your article is accepted, you may have the opportunity to make the peer review history publicly available. The record will include editor decision letters (with reviews) and your responses to reviewer comments. If eligible, we will contact you to opt in or out.***

In revising the manuscript for further consideration here, please ensure you address the specific points made by each reviewer and the editors. In your rebuttal letter you should indicate your response to the reviewers' and editors' comments and the changes you have made in the manuscript. Please submit a clean version of the paper as the main article file. A version with changes marked must also be uploaded as a marked up manuscript file.

Please also check the guidelines for revised papers at http://journals.plos.org/plosmedicine/s/revising-your-manuscript for any that apply to your paper. If you haven't already, we ask that you provide a short, non-technical Author Summary of your research to make findings accessible to a wide audience that includes both scientists and non-scientists. The Author Summary should immediately follow the Abstract in your revised manuscript. This text is subject to editorial change and should be distinct from the scientific abstract.

In addition to these revisions, you may need to complete some formatting changes, which you will receive in a follow up email. A member of our team will be in touch with a set of requests shortly. If you do not receive a separate email within a few days, please assume that checks have been completed, and no additional changes are required.

We expect to receive your revised manuscript within 1 week. Please email us (plosmedicine@plos.org) if you have any questions or concerns.

We ask every co-author listed on the manuscript to fill in a contributing author statement. If any of the co-authors have not filled in the statement, we will remind them to do so when the paper is revised. If all statements are not completed in a timely fashion this could hold up the re-review process. Should there be a problem getting one of your co-authors to fill in a statement we will be in contact. YOU MUST NOT ADD OR REMOVE AUTHORS UNLESS YOU HAVE ALERTED THE EDITOR HANDLING THE MANUSCRIPT TO THE CHANGE AND THEY SPECIFICALLY HAVE AGREED TO IT.

Please ensure that the paper adheres to the PLOS Data Availability Policy (see http://journals.plos.org/plosmedicine/s/data-availability), which requires that all data underlying the study's findings be provided in a repository or as Supporting Information. For data residing with a third party, authors are required to provide instructions with contact information for obtaining the data. PLOS journals do not allow statements supported by "data not shown" or "unpublished results." For such statements, authors must provide supporting data or cite public sources that include it.

To enhance the reproducibility of your results, we recommend that you deposit your laboratory protocols in protocols.io, where a protocol can be assigned its own identifier (DOI) such that it can be cited independently in the future. Additionally, PLOS ONE offers an option to publish peer-reviewed clinical study protocols. Read more information on sharing protocols at https://plos.org/protocols?utm_medium=editorial-email&utm_source=authorletters&utm_campaign=protocols

Please review your reference list to ensure that it is complete and correct. If you have cited papers that have been retracted, please include the rationale for doing so in the manuscript text, or remove these references and replace them with relevant current references. Any changes to the reference list should be mentioned in the rebuttal letter that accompanies your revised manuscript.

Please note, when your manuscript is accepted, an uncorrected proof of your manuscript will be published online ahead of the final version, unless you've already opted out via the online submission form. If, for any reason, you do not want an earlier version of your manuscript published online or are unsure if you have already indicated as such, please let the journal staff know immediately at plosmedicine@plos.org.

If you have any questions in the meantime, please contact me or the journal staff on plosmedicine@plos.org.

We look forward to receiving the revised manuscript by Sep 16 2026 11:59PM.

Sincerely,

Alison Farrell, Ph.D.

Senior Editor

PLOS Medicine

plosmedicine.org

------------------------------------------------------------

Requests from the Academic Editor:

1. The abstract and manuscript text are inconsistent in terms of some of the numbers.

Abstract: search ended February 10, 2025, there were 91 studies, 221 estimates, 186,613 participants, and 39 countries.

Methods: search ended June 9, 2026

Results: reported 94 studies and 226 estimates

I think the inconsistency is just because the abstract was not updated in the revision, but it probably shoudl be updated.

2. Related to the above: in the revised Results the authors say that 90 studies met inclusion criteria, 5 were excluded as duplicates, then 9 identified through reference searching. But in the PRISMA diagram there were 85 studies eligible for review then 9 identified through reference searching.

3. In the initial submission, the authors stated that UNAIDS "supported the conceptualisation of the project and reviewed the final draft before submission." But in the revised submission, the statement simply says that funders had "no role in study design, data collection and analysis, decision to publish, or preparation of the manuscript." The reason for the change is unclear to me, especially since several coauthors are UNAIDS staff.

Requests from Editors:

Please ensure that the metadata aligns with the manuscript.

Please consider moving the funnel plots to the main manuscript.

Please note that ranges or intervals should commas and not dashes.

Please note the requirement below for a DOI.

Please note that we require the inclusion of an Author Summary.

GENERAL EDITORIAL REQUESTS

"* At this stage, we ask that you include a short, non-technical Author Summary of your research to make findings accessible to a wide audience that includes both scientists and non-scientists. The Author Summary should immediately follow the Abstract in your revised manuscript. This text is subject to editorial change and should be distinct from the scientific abstract. Ideally each sub-heading should contain 2-3 single sentence, concise bullet points containing the most salient points from your study.

**In the final bullet point of the Author Summary, ‘What Do These Findings Mean?’, please include the main limitations of the study in non-technical language.

Please see our author guidelines for more information: https://journals.plos.org/plosmedicine/s/revising-your-manuscript#loc-author-summary."

* Please ensure that the Introduction ends with a clear description of the study question or hypothesis.

* Please ensure that all abbreviations are defined at first use throughout the text.

* Please confirm that all numbers presented in the abstract are present and identical to numbers presented in the main manuscript text [see relevant comments from the Academic Editor].

GENERAL

* Please include a statement regarding use or non-use of AI tools and technologies at the end of the Methods. Please specify the name(s) and version(s) of any tool(s) used and a description of their use, including how the AI-assisted or generated content was evaluated by authors, and a clear statement of which aspects of the study, article contents, data, or supporting files were affected or generated by AI tool usage. Please also include a statement specifying who retains responsibility for the final manuscript. See our guidelines for more information: https://journals.plos.org/plosmedicine/s/ethical-publishing-practice#loc-artificial-intelligence-tools-and-technologies.

* Please remove the 'conclusions' subheading from the discussion. Please also remove any other subheadings from the discussion.

"* Statistical reporting: Please revise throughout the manuscript, including tables and figures.

- Please report statistical information as follows to improve clarity for the reader ""22% (95% CI [13,28]; p</=)"".

- Please separate upper and lower bounds with commas instead of hyphens as the latter can be confused with reporting of negative values.

- Please repeat statistical definitions (HR, CI etc.) for each set of parentheses."

FUNDING STATEMENT

* The funding statement should include: specific grant numbers, initials of authors who received each award, URLs to sponsors’ websites. Also, please state whether any sponsors or funders (other than the named authors) played any role in study design, data collection and analysis, the decision to publish, or preparation of the manuscript. If they had no role in the research, include this sentence: “The funders had no role in study design, data collection and analysis, decision to publish, or preparation of the manuscript.”

COMPETING INTERESTS STATEMENT

* All authors must declare their relevant competing interests per the PLOS policy, which can be seen here: https://journals.plos.org/plosmedicine/s/competing-interests For authors with ties to industry, please indicate whether any of the interests has a financial stake in the results of the current study. Please ensure that all authors have completed the questionnaire they received by email.

DATA AVAILABILITY

* PLOS defines the “minimal data set” to consist of the data set used to reach the conclusions drawn in the manuscript with related metadata and methods, and any additional data required to replicate the reported study findings in their entirety. Authors do not need to submit their entire data set, or the raw data collected during an investigation. Please submit the following data:

The values behind the means, standard deviations and other measures reported;

The values used to build graphs;

The points extracted from images for analysis."

"* The Data Availability Statement (DAS) requires revision. For each data source used in your study:

a) If the data are freely or publicly available, note this and state the location of the data: within the paper, in Supporting Information files, or in a public repository (include the DOI or accession number).

b) If the data are owned by a third party but freely available upon request, please note this and state the institutional owner of the data set and contact information for data requests (institutional web or email address). Note that a study author cannot be the contact for the data.

c) If the data are not freely available, please describe briefly the ethical, legal, or contractual restriction that prevents you from sharing it. Please also include an appropriate institutional contact (institutional web or email address) for inquiries (again, this cannot be a study author).

* For studies in which a novel model is central to the manuscript's findings, authors are responsible for providing the source code needed to replicate the study's findings in a repository (such as GitHub, SourceForge or Bitbucket) or a cloud computing service (such as Code Ocean). Please include a citable digital object identifier (DOI) or other persistent identifier of the code (such as through linkage with Zenodo). Protection of authors’ intellectual property will not be cause for exception. Please explain in the manuscript’s Data Availability Statement how readers can access the shared code.

* Please include the statement on code availability in the data availability statement. Please see guidance here: https://journals.plos.org/plosmedicine/s/code-sharing-guidance#loc-data-availability-statement-examples

FIGURES

* Please define all elements of box plots in the figure caption - center line, box limits and whiskers.

* Please provide titles and legends for all figures and tables (including those in Supporting Information files). Please define all acronyms used in each figure or table in its corresponding legend.

ACKNOWLEDGMENTS

* Please include an Acknowledgments section in your manuscript. The Acknowledgements should not include funder information.

SRAs / MAs

"* Please report your SR/MA according to the PRISMA guidelines provided at the EQUATOR site.

http://www.equator-network.org/reporting-guidelines/prisma/

Please provide the completed PRISMA checklist as Supporting Information.

When completing the checklist, please use section and paragraph numbers, rather than page numbers.

Please add the following statement, or similar, to the Methods: ""This study is reported as per the Preferred Reporting Items for Systematic Reviews and Meta-Analyses (PRISMA) guideline (S1 Checklist)."""

* Please provide the beginning and end dates of your search.

* Please report your abstract according to PRISMA for abstracts, following the PLOS Medicine abstract structure (Background, Methods and Findings, Conclusions): https://doi.org/10.1371/journal.pmed.1001419

Comments from Reviewers:

Reviewer #2: Overall, the authors did a great job on this revision. I only have a few comments.

1. Despite the editor's request to use the 2025 framework, the authors still used the PRISMA 2020 framework. The authors may want to update the framework.

2. The authors stated that search was updated to June 9, 2026, but the abstract still said Feb 10, 2025. Please make these dates consistent.

3. The authors have substantially improved this manuscript, but causal wording still remains. For instance, "experiencing violence may increase HIV transmission risk and worsen HIV treatment outcomes" is a casual inference. The authors may want to change such statements as "experiencing violence is associated with higher HIV prevalence and poorer treatment outcomes".

Reviewer #3: I thank the authorship team for their thoughtful responses to my critiques. Their attention to detail and grace in receiving these comments should be applauded. There are a few small issues (e.g., stratum vs strata) that will be corrected in the next rounds of review, but I feel the paper is now ready for copyediting.

Any attachments provided with reviews can be seen via the following link:

[LINK]

# Author comment: Author response to Decision Letter 3

**DOI:** 10.1371/journal.pmed.1004977.r005

18 Sep 2026

Attachment Submitted filename: Editor comments.docx

# Editor report: Decision Letter 3

**DOI:** 10.1371/journal.pmed.1004977.r006

**Authors:** Alison Farrell

18 Sep 2026

Dear Dr Dawe,

On behalf of my colleagues and the Academic Editor, Alexander Tsai, I am pleased to inform you that we have agreed to publish your manuscript "Impact of violence on HIV outcomes among female sex workers: A global systematic review and meta-analysis" (PMEDICINE-D-26-00594R3) in PLOS Medicine.

Before your manuscript can be formally accepted you will need to complete some formatting changes, which you will receive in a follow up email. Please be aware that it may take several days for you to receive this email; during this time no action is required by you. Once you have received these formatting requests, please note that your manuscript will not be scheduled for publication until you have made the required changes.

Please also note that we ask you to switch the hyphens to commas in the 95% CIs in Figures 2 and 3.

Please also add the version of Claude.ai to the AI disclosure statement.

Line 250: revise 'experiences violence' to 'experiences of violence'

Line 450: 'injecting' should be 'injection'

Please add the DOI to the Data Availability Statement in the manuscript metadata, as this will appear online, below the Abstract for ease of access to data and code.

Please note that we have asked production to move the Author Summary to after the Abstract, within the manuscript file.

In the Author Summary, in "What did the researchers do and find?" please temper the claim of primacy in the first bullet point by adding 'to our knowledge' to read " We conducted, to our knowledge, the first..."

In the meantime, please log into Editorial Manager at http://www.editorialmanager.com/pmedicine/, click the "Update My Information" link at the top of the page, and update your user information to ensure an efficient production process.

PRESS

We frequently collaborate with press offices. If your institution or institutions have a press office, please notify them about your upcoming paper at this point, to enable them to help maximise its impact. If the press office is planning to promote your findings, we would be grateful if they could coordinate with medicinepress@plos.org. If you have not yet opted out of the early version process, we ask that you notify us immediately of any press plans so that we may do so on your behalf.

We also ask that you take this opportunity to read our Embargo Policy regarding the discussion, promotion and media coverage of work that is yet to be published by PLOS. As your manuscript is not yet published, it is bound by the conditions of our Embargo Policy. Please be aware that this policy is in place both to ensure that any press coverage of your article is fully substantiated and to provide a direct link between such coverage and the published work. For full details of our Embargo Policy, please visit http://www.plos.org/about/media-inquiries/embargo-policy/.

To enhance the reproducibility of your results, we recommend that you deposit your laboratory protocols in protocols.io, where a protocol can be assigned its own identifier (DOI) such that it can be cited independently in the future. Additionally, PLOS ONE offers an option to publish peer-reviewed clinical study protocols. Read more information on sharing protocols at https://plos.org/protocols?utm_medium=editorial-email&utm_source=authorletters&utm_campaign=protocols

Thank you again for submitting to PLOS Medicine. We look forward to publishing your paper.

Sincerely,

Alison Farrell, Ph.D.

Senior Editor

PLOS Medicine
